# Nutrition Interventions for Pediatric Obesity Prevention: An Umbrella Review of Systematic Reviews

**DOI:** 10.3390/nu15245097

**Published:** 2023-12-13

**Authors:** Mary Rozga, Deepa Handu

**Affiliations:** Academy of Nutrition and Dietetics, 120 S. Riverside Plaza, Suite 2190, Chicago, IL 60606-6995, USA; dhandu@eatright.org

**Keywords:** pediatrics, nutrition, obesity, dietitian, primary prevention, diet, exercise, systematic review, schools

## Abstract

Nutrition interventions to prevent pediatric obesity can help to establish healthy habits to improve current and future health. The objective of this umbrella review of systematic reviews (SRs) is to examine the impact of obesity prevention interventions with a nutrition component on body mass index measures, overweight/obesity prevalence, and cost-effectiveness in participants 2–17 years old. Grading of Recommendations Assessment, Development and Evaluation (GRADE) methods were used, and this umbrella review was registered on PROSPERO (CRD42023443033). Included SRs were required to search ≥2 databases and to assess the risk of bias (RoB) of primary studies, and they were published 2017–June 2023. Database searches identified 4776 articles, and 31 SRs were included. In all age groups combined, interventions with both nutrition and physical activity were effective and cost-effective in all settings combined, and in the community setting specifically. In children ≤5 years old, interventions in the home and family, community, and healthcare settings demonstrated some efficacy, whereas in children 6–12 years old, school interventions were most effective. Evidence with individuals 13–17 years was limited. The certainty of evidence was generally low due to RoB in included studies, inconsistency, and imprecision. Pediatric obesity prevention interventions with nutrition should be tailored to the developmental stage to ensure appropriateness and efficacy.

## 1. Introduction

Pediatric obesity can impact physical and psychological health and can lead to several adverse health conditions, including type 2 diabetes mellitus (T2DM), cardiovascular problems, and gastroesophageal reflux [1]. Accordingly, a Healthy People 2030 goal is to reduce the proportion of children and adolescents with obesity from its current rate of 19.7% [2]. The United States Preventive Task Force recommends that children and adolescents be screened for obesity and referred for or offered comprehensive behavioral interventions, which may include parent involvement and instruction on nutrition and physical activity [3]. The nutrition component of interventions is ideally led or designed by dietitians, who may provide nutrition counseling in child-based settings, develop and deliver theory-based nutrition education programs, and implement environmental and policy changes to improve access to healthy foods [4].

Interventions to prevent pediatric obesity can help to establish healthy habits to improve current and future health. Childhood and adolescence are stages of dynamic growth in which developmental skills, interests, and emotional maturity vary between peers and within individuals over time. Whereas younger children rely more on caregivers to learn how to eat and prepare food, older children become increasingly autonomous in choosing foods [5]. Thus, for interventions to be effective, they must accommodate diverse contexts and changing needs, including level of involvement in home life, in school, and with peers. 

A recent overview of systematic reviews (SRs), also called an umbrella review, found that interventions for pediatric obesity prevention for children 6–12 years old improved BMI measures [6]. Another overview of reviews from 2019 found no overall effect of prevention interventions on BMI measures in pediatric participants but did not discuss results by type of intervention or age [7]. These umbrella reviews included 3–5 SRs each, though a recent scoping review identified many current SRs addressing a wide range of nutrition interventions to prevent pediatric obesity [8]. A 2020 overview of reviews identified 13 SRs that generally demonstrated little to no effect of prevention interventions on BMI measures in adolescents [9]. In 2021, the Academy of Nutrition and Dietetics conducted an umbrella review on pediatric obesity prevention interventions with nutrition to inform a Position Paper for nutrition practitioners, funders, and policymakers working to prevent pediatric obesity [4]. However, the umbrella review was never published. To provide those working in the nutrition field with detailed evidence to assess and to inform practice, the current manuscript provides a comprehensive account of updated evidence on nutrition interventions to prevent pediatric obesity. 

Given the importance of facilitating healthy behaviors in children and adolescents and the uncertainty about which types of interventions are effective at which developmental stages, a comprehensive umbrella review is needed to inform practitioners, program funders, and policymakers working in the nutrition field about effective methods to prevent obesity throughout childhood and adolescence. The objective of this umbrella review is to examine the research question: In presumably healthy children and adolescents in the general population, what is the impact of pediatric obesity prevention interventions with nutrition on BMI measures, overweight and obesity incidence, and cost-effectiveness?

## 2. Methods

This umbrella review of SRs was designed using a social-ecological model [10], used methods described by the Cochrane Collaboration [11] and the Academy of Nutrition and Dietetics [12], and was reported according to the PRIOR checklist for overviews of SRs [13]. This umbrella review was registered on PROSPERO (CRD42023443033) [14]. 

### 2.1. Eligibility Criteria

Eligibility criteria are described in Table 1. Included SRs addressed all aspects of the Population-Intervention-Comparison-Outcome (PICO) question: In presumably healthy children and adolescents (2–17 years) what is the impact of pediatric obesity prevention interventions that include nutrition, compared to no intervention, on BMI measures, prevalence of overweight and obesity and cost-effectiveness? SRs were excluded if they targeted individuals with diagnosed diseases, including those with overweight or obesity. SRs were eligible if they searched at ≥2 databases, assessed the risk of bias (RoB) of included primary studies, and were published after January 2017 to ensure the inclusion of recent primary research. SRs conducting meta-analysis or grading the certainty of evidence (CoE) for outcomes of interest were prioritized. When SRs using gold-standard methods were not available, SRs without these methods were included [8].

### 2.2. Information Sources 

Authors utilized search terms from a supporting scoping review [8], which was designed by an information specialist for Medline (Ebsco, Ipswich, MA, USA), CINAHL (Ebsco), Cochrane Database of Systematic Reviews (Ebsco), and Food Science Source (Ebsco) databases. This search was updated by M.R. The full search plan can be found in Supplementary Tables S1–S5.

### 2.3. Study Selection

A sample of titles and abstracts were independently screened by M.R. and D.H. using Rayyan screening software (https://rayyan.ai/cite), and >80% agreement was achieved [15]. Therefore, the remaining titles and abstracts were screened by M.R., consulting with D.H. as needed. SRs included from the title and abstract screening were reviewed independently and in full by M.R. and D.H. Any disagreements between reviewers were resolved by consensus. 

### 2.4. Data Collection

Data were extracted from the SRs by M.R. and cross-checked by D.H. Data were extracted onto a study-characteristics table and included: bibliographic information; participant ages; number and study designs of articles included in the SR; description of the intervention of interest and comparison groups; setting (e.g., school, healthcare); reported outcomes of interest; if the SR conducted meta-analysis or graded CoE, and the tool used to assess the RoB in primary studies. Interventions were required to have a nutrition component. However, results from interventions that additionally included physical activity were prioritized when interpreting findings, as multi-component interventions may be more effective [16]. For each study, quantitative and narrative results were extracted for each outcome of interest in each age group and setting of interest. In addition, primary studies included for each SR were compared to primary studies included in other SRs examining interventions in the same settings and age groups. 

### 2.5. Quality Assessment

Included SRs were required to assess the RoB of included primary studies (Table 1). Additionally, each included SR was assessed for quality using the AMSTAR2 tool [17]. SR quality was determined independently by two reviewers and discrepancies were resolved through consensus. 

### 2.6. Synthesis of Results and Certainty Assessment

The inclusion process for this study was documented in a PRISMA flowchart [18]. Characteristics and results from each included SR were described in tables. If SRs included interventions from all settings or age groups, results were categorized as “all settings” or “all age groups combined” only unless sub-group analysis was provided for specific settings or age groups. SRs examining interventions in a specific setting (e.g., school) or age group (e.g., 6–12 years) were included in results for those categories only. The highest-quality SRs as determined by AMSTAR2 ratings and SRs that were the most comprehensive were used to inform conclusions. However, we were not able to conduct novel meta-analysis of included primary studies, as included SRs did not report sufficient data. 

If SRs reported CoE using the Grading of Recommendations Assessment, Development and Evaluation (GRADE) method, this grade was used to report CoE in this umbrella review. If the SRs used a different method or did not assess CoE, evidence was graded using the GRADE method [19], which was documented in summary of findings tables. CoE was graded as high, moderate, low, or very low [19]. Heterogeneity and sensitivity analyses conducted in the included SRs were used to inform conclusions. 

## 3. Results

**Figure 1:**
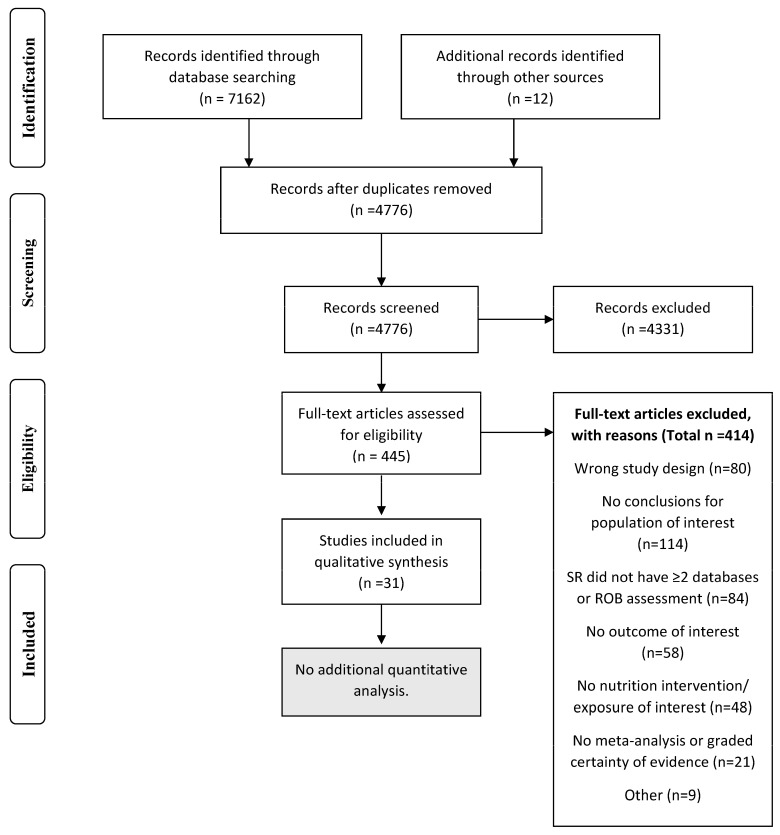
PRISMA flow chart demonstrating the study inclusion process for the umbrella review of systematic reviews on nutrition interventions to prevent pediatric obesity.

There were 4776 unique articles identified by the database searches, and authors reviewed the full text of 445 articles. A list of articles excluded during full-text review, including reasons for exclusion is available in Supplementary Table S6. Thirty-one SRs were included in this umbrella review [20,21,22,23,24,25,26,27,28,29,30,31,32,33,34,35,36,37,38,39,40,41,42,43,44,45,46,47,48,49,50]. SRs examined nutrition interventions for pediatric obesity prevention in all settings combined [20,22,30,33,34,43,44,47] or in the home and family [20,22,35], healthcare [22,37,49,50], school [20,22,23,25,27,28,29,36,38,40,41,42,46], or community settings [21,22], or examined the efficacy of food assistance programs [24,26,27,31,32,39] or electronic interventions [45,48]. The study selection process is described in Figure 1. Study characteristics are described in Table 2, and SR quality, as measured by the AMSTAR2 tool, can be found in Supplementary Table S7. Some SRs reported findings for more than one age group or settings category (Table 2). 

### 3.1. All Settings

Eight included SRs examined the impact of nutrition interventions for pediatric obesity prevention in all settings, including in all ages combined [20,34,43,47], or in those ≤5 years [22], 6–12 years [20,22,44], or 13–17 years [22,30,33]. The eight SRs analyzed data from a total of 142 primary studies, and 33 of these studies were represented in more than one SR.

#### 3.1.1. Age Groups Combined

Four SRs examined the effect of nutrition interventions for pediatric obesity prevention in combined age groups [20,34,43,47]. Results are described in Table 3 and CoE is described in Table 4. Salam et al., published in 2020, found that nutrition and physical activity interventions reduced BMI z-score [mean difference (MD) (95 % confidence interval (CI)):−0.12 (−0.18, −0.06)] and BMI [−0.41 kg/m^2^ (−0.06, −0.21)], and CoE was low due to high heterogeneity in results [43]. Long et al. 2021 agreed with these results but found smaller effect sizes [34]. In Abdel Rahman et al. 2018, there was no effect of the interventions to reduce sugar-sweetened beverage intake on BMI z-scores in three primary studies [20]. Specchia et al. 2018 reported that multi-component, multi-level, or multi-setting interventions reduced overweight and obesity prevalence [47]. In the supplementary materials, Salam et al. also reported effects on obesity prevalence, but there were errors in the analysis. Salam et al. 2020 [43] was the only SR to examine the cost-effectiveness of nutrition interventions to prevent obesity, and five out of six of the included primary studies demonstrated cost-effectiveness or cost savings. Salam et al. 2020 [43] and Specchia et al. 2018 [47] had a low quality score and the remaining SRs had critically low quality scores (Table S7). 

#### 3.1.2. Specific Age Groups

In SRs examining children ≤5 years, Brown et al. found moderate CoE that interventions including both diet and physical activity reduced BMI z-score [−0.07 (−0.14, −0.01)] and BMI [−0.11 kg/m^2^ (−0.21, 0.00)] compared to control groups [22]. Two SRs targeted children 6–12 years old [22,44]. Interventions with both diet and physical activity reduced BMI z-score [−0.05 (−0.10, −0.01)], but not BMI [−0.05 kg/m^2^ (−0.11, 0.01)] in 6–12-year-old children, and CoE was low (Table 3) [22]. Seral-Cortes et al. found that health programs targeting obesity prevention with nutrition and physical activity did not affect BMI z-score or BMI [44]. Brown had a high quality score and Seral-Cortes had a critically low quality score (Supplementary Table S7).

Three SRs examined the effect of nutrition interventions in all settings for adolescents 13–17 years old [22,30,33]. Brown et al. and Hayba et al. were high quality SRs and Kornet van der Aa was of moderate quality. Hayba et al. and Kornet van der Aa targeted adolescents from under-represented groups. Nutrition and physical activity interventions did not affect BMI z-score or BMI, and CoE was low [22,30]. Overweight and obesity prevalence decreased in the intervention group compared to the control group in one study [33] and CoE was very low (Table 3 and Table 4). 

Based on the current evidence, nutrition and physical activity interventions to prevent pediatric obesity may reduce BMI z-score and may be cost-effective for participants <18 years old. Interventions in all settings were most effective for individuals ≤12 years old. Obesity prevention programs that were multi-component, multi-level and/or within multiple settings may reduce overweight/obesity prevalence. 

### 3.2. Home and Family Setting

Three SRs examined the impact of interventions of interest in the home and family setting [20,22,35] in all ages combined [35] and for children ≤5 years old [22], 6–12 years old [20,22], and 13–18 years old [22]. In the three SRs, 12 primary studies were represented, and there was no overlap in primary studies between SRs. 

#### 3.2.1. Age Groups Combined

Morgan et al. 2020 reported no pooled effect of interventions with children as active participants and caregivers involved in at least one aspect of the intervention compared to interventions in which a caregiver was not involved on BMI [standardized mean difference (95%CI): 0.05 (−0.04, 0.15) I^2^ = 0%] or overweight/obesity prevalence [relative risk (95% CI): 1.02 (0.89, 1.17)] (Table 3) [35]. CoE was low (Supplementary Table S8). 

#### 3.2.2. Specific Age Groups

Brown et al. reported that interventions in the home and family setting improved BMI [MD (95% CI): −0.33 kg/m^2^ (−0.55, −0.10)] but not BMI z-score [−0.13 (−0.35, 0.09)] in children ≤5 years old [22]. In 6–12-year-old children, there was no effect on BMI z-score compared to the control group in one RCT [0.03 (−0.04, 0.10)] [22], and CoE was low. Brown et al. found no effect of nutrition and physical activity intervention in the home setting on BMI z-score in 13–18 years olds [0.06 (−0.13, 0.26)] [22], and CoE was very low (Table 3 and Table S8). 

Based on the current evidence, nutrition and physical activity interventions in the home setting or with a caregiver may be most effective for improving BMI measures in children ≤5 years old but may have little or no effect in individuals 6–18 years old compared to no intervention. 

### 3.3. Healthcare Setting

Four SRs examined pediatric obesity prevention interventions with nutrition in healthcare settings [22,37,49,50], and included SRs with all age groups combined [49] and for children ≤5 years old [22,37,50]. The four SRs included 12 total primary studies, and there was no overlap in primary studies between SRs. 

#### 3.3.1. Age Groups Combined

In an SR by Tissot et al. reported in 2021, primary care-led interventions in individuals 10–19 years old reduced BMI percentile or z-scores in four studies, there was no effect in four studies, and findings were unclear in the remaining study. No meta-analysis was conducted and CoE was very low due to RoB, inconsistency in findings between studies, and small sample sizes (Supplementary Table S9) [49]. 

#### 3.3.2. Specific Age Groups

Three SRs identified interventions provided for children ≤5 years old. BMI z-score improved in the intervention groups compared to control groups in 2–4-year-old Latino children [MD (95%CI): −0.24 (−0.46, −0.02)] [22]. BMI z-score improved in children when nurses trained childcare staff in obesity prevention compared to controls −0.14 (−0.26 to −0.02) [50]. In the final SR, there was no significant difference in children’s prevalence of overweight according to group assignment in children ≤5 years old [37]. 

Based on the current evidence, nutrition and physical activity interventions delivered in the healthcare setting may reduce BMI z-scores in children ≤5 years old, but the effect on overweight or obesity prevalence and effect in older children and adolescents is uncertain. 

### 3.4. School Setting

Thirteen SRs examined the impact of obesity prevention interventions with nutrition in the school setting in all age groups combined [20,23,27,28,29,38,40], children ≤5 years old [22], children 6–12 years old [22,25,36,41,42,46] and adolescents 13–18 years old [22]. No studies reported the cost-effectiveness of these interventions. There were 139 unique primary studies analyzed in the 13 SRs combined, and 36 primary studies were represented in more than one SR. 

#### 3.4.1. Age Groups Combined

Of the seven SRs that examined the impact of nutrition interventions in age groups combined [20,23,27,28,29,38,40], Nury et al. 2021 [38] had a high quality score, Gonclaves et al. 2021 and Durão et al. 2023 had a moderate quality score [28,29], and the remaining SRs had a critically low quality score (Table S7). Nury et al. 2021 demonstrated little to no effect of school nutrition interventions on BMI z-score [MD (95% CI): −0.09 (−0.18, 0.00)], BMI [0.03 kg/m^2^ (−0.10, 0.16)], or overweight and obesity prevalence [OR (95% CI): 1.19 (0.95, 1.49)] (Table 3). CoE was low due to RoB in included studies and imprecision [38]. Results were similar in SRs examining interventions influencing school food environment [29,52] and reducing sugar-sweetened beverage intake in schools [20]. However, three SRs with a critically low quality score found improvements in outcomes from school interventions (Table 3) [23,27,40].

#### 3.4.2. Specific Age Groups

Brown et al. described no difference in BMI z-score [MD (95%CI): −0.04 (−0.09, 0.01)] or BMI [−0.05 kg/m^2^ (−0.14, 0.05)] for children ≤5 years old participating in nutrition and physical activity interventions in the childcare/preschool setting compared to control groups [22]. Additionally, Brown et al. demonstrated no effect on BMI z-score [MD (95%CI): 0.00 (−0.06, 0.06)] or BMI [−0.02 kg/m^2^ (−0.10, 0.05)(I^2^ = 58%)] in 13–18-year-olds (Table 3), but heterogeneity of results was very high (Supplementary Table S10).

Six SRs examined nutrition interventions for obesity prevention in children 6–12 years old [22,25,36,41,42,46]. Brown et al. 2019 and Smit et al. 2023 had a high quality score [22,46], and the remaining SRs had a critically low quality score (Table S7). Smit et al. was more recent, but Brown et al. was more comprehensive and there were only two primary studies that overlapped between these SRs. Brown et al., published in 2019, demonstrated that interventions with both diet and physical activity reduced BMI z-score [−0.04 (−0.08, −0.01)], but there was no difference in BMI [−0.04 kg/m^2^ (−0.10, 0.02)] [22]. Smit et al. found no effect of primary school-based obesity prevention interventions on BMI z-score [MD (95% CI): −0.08 (−0.20, 0.05)], BMI [0.06 (−0.38, 0.50)], or overweight prevalence (studies not pooled) in children 6–12 years old. Smit rated all outcomes as having very low CoE (Table 3) [46]. 

Nally et al. 2021 demonstrated an improvement in BMI z-scores and BMI, respectively, in children 5–12 years old [36]. Rochira et al., published in 2020,examined school garden interventions in children 6–13 years old and found an improvement in BMI percentile, but no impact on BMI z-score or BMI [42]. Cerrato-Carretero et al. 2021 and Qi et al. 2021 also found a null impact on BMI z-scores [41] and BMI [25,41]. 

Based on the current evidence, in all age groups combined, nutrition and physical activity interventions in the school setting may not reduce BMI z-score, BMI, or overweight or obesity prevalence. Interventions in school settings likely reduce BMI z-score in children 6–12 years old. 

### 3.5. Community Setting

Two SRs examined the impact of obesity prevention interventions with nutrition in the community setting in all children combined [21], and in children ≤5 years old [22], 6–12 years old [21,22], and 13–17 years old [22]. One SR reported on the cost-effectiveness of community interventions in Australia [21]. Twenty-three primary studies were identified in these SRs, and there was no overlap between SRs. 

#### 3.5.1. All Age Groups

In the SR by Ananthapavan et al. published in 2018, community interventions decreased BMI z-score [MD (95% CI): −0.07 (−0.13, −0.01)] in Australia [21], and CoE was low (Table 4). Using results from included studies, authors analyzed cost-effectiveness and found that the mean incremental cost-effectiveness ratio (ICER) was AUD 8155 (AUD 237 to AUD 81,021) per health-adjusted life year, and there was a 95% probability of interventions being cost-effective at the defined threshold [21]. Evidence certainty was low (Supplementary Table S11).

#### 3.5.2. Specific Age Groups

Brown et al. found that diet and physical activity interventions had no effect on BMI z-score in children ≤5 years old, but reduced BMI by −0.59 kg/m^2^ (−0.94, −0.24) [22] (Table 3). 

Two SRs reported on intervention efficacy in children around 6–12 years old [21,22]. Brown et al. found no effect of community interventions, including nutrition and physical activity, on BMI z-score [−0.04 (−0.39, 0.31); I^2^ = 94%] or BMI kg/m^2^ [−0.08 (−0.29, 0.13); I^2^ = 25%] [22]. Ananthapavan et al. found an improvement in BMI z-score, but their total sample size was unclear. Ananthapavan et al. 2018 found no effect on BMI z-score in adolescents 12–18 years old [MD (95% CI): −0.02 (−0.07, 0.03)] [21]. Brown had a high quality score and Ananthapavan had a critically low quality score. CoE ranged from low to very low (Table 3 and Table S11). 

Based on the current evidence, pediatric obesity prevention interventions with nutrition in the community setting may improve BMI z-score and be cost-effective in all age groups combined. Nutrition and physical activity interventions in the community setting may decrease BMI in children ≤5 years old, but interventions were ineffective, or evidence was lacking for older children and adolescents. 

### 3.6. Federal Food Assistance Programs

Six SRs examined the impact of federal food assistance programs on obesity prevention. Programs assessed included the Supplemental Nutrition Program for Women, Infants and Children (WIC) [24], universal school meals [26,27], the Supplemental Nutrition Assistance Program (SNAP) [31], the Child and Adult Care Food Program (CACFP) [32], and food assistance programs in general [39]. Twenty-two primary studies were represented in total and only one study was cited in more than one SR. CoE is described in Supplementary Table S12.

#### 3.6.1. All Age Groups

Hudak et al., published in 2019, investigated the impact of SNAP in participants 2–18 years old. Of the studies that addressed selection bias, results were heterogeneous, including lower risk of overweight or obesity in participants who were boys or younger girls but increased risk for girls 5–18 years old or who were long-term participants. Results were not pooled in the meta-analysis [31]. 

Kenney et al. 2023 identified one study with 4050 participants ages 2–18 years old that reported that CACFP participation was not associated with the prevalence of overweight and obesity [32]. 

Both Cohen et al. (2021) and Dabravolskaj et al. (2020) examined the impact of universal school meals. Cohen et al. demonstrated mixed findings for the effect on BMI z-score, and no effect on BMI or obesity prevalence [26]. Dabravolskaj et al. agreed with these findings [27].

#### 3.6.2. Specific Age Groups

Caulfield et al. 2022 examined the impact of the revised 2009 WIC food package for children ≤5 years old and identified three studies with >16 million participants [24]. The authors concluded that the revised food package reduced overweight and obesity prevalence and rated CoE as low. 

Olstad et al., published in 2017, evaluated primary studies targeting government policies for disadvantaged populations. Primary studies investigating the SNAP program were excluded, and just one primary quasi-experimental study was identified that reported that the USDA Fresh Fruit and Vegetable Program decreased BMI z-score and BMI in elementary school students after four years [39]. 

Based on the current evidence, few SRs assessed the impact of federal food assistance programs on the prevention of pediatric obesity. The 2009 revised WIC package reduced obesity prevalence in young children, and a USDA program providing fresh fruits and vegetables outside of the school reduced BMI z-score and BMI in elementary-aged children. The impact of the SNAP program was mixed, depending on the sub-population examined. The CACFP program and universal school meals did not impact BMI outcomes or obesity prevalence.

### 3.7. Electronic Interventions

Two SRs examined the effects of electronic interventions with nutrition to prevent pediatric obesity [45,48]. There were eight primary studies represented in the SRs with no overlap in primary studies between SRs. Suleiman-Martos et al. 2021 identified five RCTs, three of which demonstrated an improvement of gamification on BMI z-scores and two of which found no effect. Results from two of these trials were pooled, and there was no effect on BMI z-scores [MD (95% CI): −0.05 (−0.21, 0.11)] [48]. Silva et al. 2022 found no effect of computer-based nutrition interventions compared to controls on BMI in 10–19-year-olds in three trials [−0.02 kg/m^2^ (−0.18, 0.14)] (Table 3, Supplementary Table S13) [45]. There may be no effect of electronic nutrition interventions on BMI or BMI z-score in children and adolescents. 

## 4. Discussion

**Figure 2:**
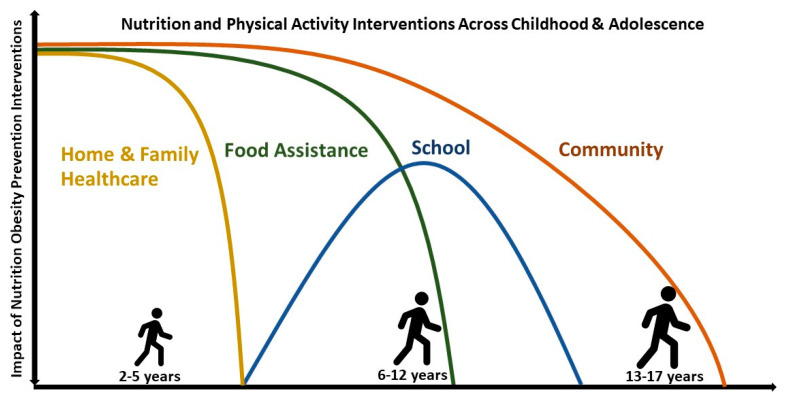
Impact of obesity pediatric prevention interventions with nutrition according to age and intervention setting. Nutrition and physical activity interventions in all settings and age groups combined are effective in improving BMI measures and overweight and obesity prevalence, as were interventions in all age groups combined in the community setting. Interventions in the home and family setting and in healthcare settings were beneficial for children ≤5 years old, school interventions were most effective for children 6–12 years old, and there is evidence of benefits from food assistance programs in children ≤12 years old. Beyond the impacts of interventions seen in all age groups combined, little is known about effective interventions for adolescents 13–17 years old.

This umbrella review analyzed 31 recent SRs examining the impact of pediatric obesity prevention interventions with a nutrition component. In SRs with pediatric individuals aged 2–17 years combined, interventions with both nutrition and physical activity were effective and cost-effective in all settings combined, and in the community setting specifically. In children ≤5 years old, interventions in the home and family, community, and healthcare settings all demonstrated some efficacy, as did the 2009 revised WIC package. In children 6–12 years old, interventions in schools were most effective and a federal food assistance program providing fresh fruits and vegetables also may be effective. Limited evidence in individuals 13–17 years old did not demonstrate efficacy for nutrition interventions aiming to prevent obesity (Figure 2). CoE was generally low due to RoB in included studies, inconsistency in results between studies, and imprecision in findings. 

A 2019 umbrella review of pediatric obesity prevention interventions demonstrated that mixed interventions were most effective in improving cardiovascular profile, but had little impact on BMI, though efficacy was not examined according to intervention type or participant age [7]. In another recent umbrella review, Denova-Gutierrez [6] identified five SRs targeting pediatric obesity prevention in children 6–12 years old and described that multi-component interventions, including nutrition, physical activity, and behavior change were most effective in preventing pediatric overweight and obesity [6]. However, the authors also described a gap in methodological quality in primary studies, which prevents the establishment of robust recommendations. This umbrella review supports and expands upon these findings by demonstrating the efficacy of obesity prevention interventions with at least a nutrition component in specific age groups and settings, thus providing a potential path for effective obesity prevention interventions throughout childhood and adolescence. 

The impacts of pediatric obesity interventions identified in this umbrella review were heterogeneous, which can be explained by the wide variety of interventions delivered and of methods used to implement interventions. Future SRs and umbrella reviews may focus on the efficacy of specific implementation strategies for nutrition interventions to provide robust guidance on the best methods for delivering interventions to children and adolescents in the general population. 

Among the target audiences for this umbrella review are nutrition program funders and policymakers, as these individuals are responsible for facilitating effective interventions to prevent pediatric obesity by using available resources most efficiently. However, there was very little evidence available about the cost-effectiveness of pediatric obesity prevention programs with nutrition to support policy decisions. After the search was performed for this umbrella review, Sultana et al. published a SR in 2023, examining the economic evidence for community-based interventions for pediatric obesity prevention [53]. Authors identified five studies conducting cost-utility analysis, three of which found interventions to be cost-effective. An additional primary study reported a cost-saving return-on-investment ratio [53]. These results were consistent with those found by Ananthapavan et al. in 2019, who reported that community interventions may be cost-effective [21]. Disease prevention may be difficult to prioritize in the context of what seem like more pressing health needs. More research investigating the cost-efficacy of pediatric obesity prevention programs is crucial for demonstrating that facilitating healthy lifestyle behaviors can prevent not only the suffering associated with obesity-related diseases but also the financial costs of treating these diseases. 

This umbrella review utilized a social-ecological model to examine the contexts in which interventions were most effective. However, a space that is not typically represented in the social-ecological framework is that of digital and electronic media, which extend throughout home, school, and community settings and are an integral part of food assistance programs. There were very few SRs examining the efficacy of electronic interventions, and neither of those included in this study found that electronic interventions were efficacious in preventing obesity. More research is needed to leverage the time children and adolescents are spending with electronic devices to contribute to healthy lifestyle behaviors. 

### 4.1. Strengths and Limitations 

Results from this umbrella review provide a comprehensive picture of the impact of pediatric obesity prevention programs with a nutrition component and demonstrate the need for a multi-faceted approach to establishing healthy habits that are dynamic according to needs and preferences throughout childhood and adolescence. This umbrella review utilized rigorous methods, including a systematic search, a screening process, and rigorous evidence synthesis. The wide breadth of research examined allows for the comparison of quantitative evidence from the highest-quality SRs available and can guide policymakers to support evidence-based programs. Finally, although it was not possible to perform novel meta-analyses due to the lack of information reported in many SRs, this umbrella review graded a CoE for each age group in each setting, which provides a common metric by which to compare evidence availability and efficacy. 

A limitation of this umbrella review was the gap in the underlying research, particularly on effective interventions for teenagers in the general population, which was also demonstrated in an overview of SRs by Flodgren et al. published 2020 [9], and in research on electronic and food assistance interventions. Another limitation was the limited outcomes examined in this umbrella review. Although focusing on BMI measures and overweight and obesity prevalence allowed for the inclusion of a broad evidence base and comparisons between SRs, the goal of obesity prevention programs is not to change a number on a scale or chart, but rather to improve the health and well-being of children and adolescents now and in their futures. Thus, future studies and SRs should aim to assess outcomes that are more client-centered such as the development of T2DM, quality of life, or academic achievements. 

### 4.2. Implications for Practice

Nutrition practitioners are a key target audience of this umbrella review. This review highlights that efforts to prevent pediatric obesity may be successful when delivered in the context of multi-component interventions that include nutrition. Although interventions ideally include interdisciplinary practitioners, nutrition practitioners can and should encourage the meeting of national physical activity recommendations [54] in addition to providing nutrition interventions. Practitioners should be aware that the efficacy of certain interventions varies greatly according to participant age. Thus, a life-course approach to obesity prevention should be implemented to consistently deliver the most effective interventions and the most optimal developmental stage to ensure long-term benefits into adulthood. Nutrition practitioners, funders, and policymakers should advocate for increased access to effective prevention services for children across developmental stages and settings [4]. 

## 5. Conclusions

Multi-component pediatric obesity prevention interventions with a nutrition component may improve BMI outcomes and overweight and obesity prevalence and may be cost-effective. Interventions in different settings may have varying efficacy for different age groups, and obesity prevention approaches should be tailored to developmental stages to ensure appropriateness and efficacy. Although interventions in the home, healthcare, and community settings have proven efficacious for young children and school interventions have proven efficacious for elementary-aged children, it is unclear which type of interventions may be effective for teens. Program funders and policymakers can facilitate investment in a life-course approach to disease prevention by advocating for the implementation of interventions tailored to developmental stages and research investigating effective methods in adolescents. 

## Figures and Tables

**Table 1:** Eligibility Criteria for Umbrella Review of Systematic Reviews Examining the Effect of Nutrition Interventions to Prevent Pediatric Obesity.

	Inclusion Criteria	Exclusion Criteria
Peer-Review Status	Peer-reviewed	Non-peer-reviewed articles
Population	Humans Children and adolescents (aged 2 to 17 years)	Animal studies Children aged < 2 years or adults ≥ 18 years old
Setting	Any settings, including those in the home and family, healthcare, school, and community settings.	Inpatient or acute care, inpatient rehab.
Health Status	Presumably healthy (no major co-morbidities) or part of the “general” population (e.g., school setting).	Studies targeting participants with any condition or disease that limits application to the general population, including but not limited to individuals with ADD/ADHD; asthma; autism; celiac disease/IBD/IBS; critical illness; eating disorders or disordered eating; food allergies; pregnancy; CKD, ESRD and/or renal dialysis; cancer, current or survived; heart failure; spinal cord injury; cachexia; liver disease; past surgery, including bariatric surgery; current respiratory therapy; type 1 diabetes mellitus.
Interventions/ Exposures	Nutrition interventions, with or without physical activity or other components. Programs such as food assistance programs or school nutrition programs.	SRs examining observational exposures other than programs or policies implemented in the settings indicated. SRs not requiring a nutrition component.
Comparators	Includes either comparison to a control group or pre–post measurements for longitudinal cohort studies for programs.	No comparison (e.g., prevalence of nutrition outcomes only). SRs include primarily cross-sectional studies with no stratification for longitudinal studies.
Study Design Preferences	SRs and meta-analysis SRs must search at least two databases and assess risk of bias/quality for each included study. SRs must conduct meta-analysis and/or grade the certainty of evidence. SRs not conducting meta-analysis or grading certainty of evidence will only be included if no other SRs are available for the specific setting and outcome examined.	Narrative reviews, commentary/letters to the editor; primary studies; guidelines not based on a systematic review. SRs that describe a population only (e.g., prevalence of malnutrition) or are based on cross-sectional studies only. Umbrella reviews (SRs of SRs). SRs not reporting meta-analysis or certainty of evidence when higher-quality evidence is available.
Outcome	BMI measures, prevalence, and incidence of overweight or obesity, cost-effectiveness.	Outcomes not specified in inclusion criteria.
Year Range	January 2017–8 June 2023	Prior to January 2017 or later than the search date of 8 June 2023
Language	Limited to articles in English.	Articles not published in English.
Location	Includes studies from countries with developed economies.	Does not include countries with developed economies.

ADD—attention deficit disorder; ADHD—attention deficit hyperactivity disorder; BMI—body mass index; CKD—chronic kidney disease; ESRD—end stage renal disease; IBD—irritable bowel disease; IBS—irritable bowel syndrome; SRs—systematic reviews.

**Table 2:** Study characteristics of included systematic reviews examining the impact of nutrition interventions for pediatric obesity prevention.

Systematic Review	Population	Study Designs Included	Intervention	Setting or Context	Outcomes of Interest Reported	Outcomes with Meta-Analysis	Outcomes with Certainty of Evidence	RoB Tool	Overall Confidence in Results from AMSTAR2
Abdel Rahman et al., 2018 [20]	Aged 4–16 years	RCTs	Educational or behavioral interventions to reduce sugar-sweetened beverage intake	School, Home and Family	BMI z-score, Obesity Prevalence	BMI z-score	None	Cochrane Risk of Bias tool	Critically Low
Ananthapavan et al., 2018 [21]	Aged 5–18 years	RCTs, Cluster-RCTs, quasi-experimental, cohort	Community-based obesity prevention interventions	Community	BMI z-score, Cost-effectiveness	BMI z-score	None	Effective Public Health Practice Project Quality Assessment	Critically Low
Brown et al., 2019 [22]	Aged 0–5, 6–12, and 13–18 years	RCTs	Educational, health promotion, psychological, family, behavioral therapy, counseling, management strategies	Community, school, home, healthcare	BMI z-score, BMI	BMI z-score, BMI	BMI z-score, BMI	Risk of Bias tool (Cochrane)	High
Buchanan et al., 2023 [23]	Aged, 5–18 years	Controlled trials, quasi-experimental with a comparison group, time–series, before –after study, or post-only studies with a comparison group	Programs or policies aimed at school meals, or fruit/vegetable snack programs with physical activity/education	School	BMI z-score, Overweight/Obesity Prevalence	BMI z-score, Overweight/Obesity Prevalence	None	Community Preventive Services	Critically Low
Caulfield et al., 2022 [24]	Aged <5 years	Quantitative and qualitative studies	WIC participation	Food Assistance	Obesity prevalence	None	Obesity Prevalence	Effective Public Health Practice Project	Moderate
Cerrato-Carretero et al., 2021 [25]	Aged 6–12 years	RCTs	Dietary interventions or education combined with physical activity	School	BMI	BMI	None	Risk of Bias tool (Cochrane)	Critically Low
Cohen et al., 2021 [26]	Aged 5–18 years	Quantitative research articles	Universal school meals	Food Assistance Programs, School	BMI	None	None	Newcastle-Ottawa Scale	Low
Dabravolskaj et al., 2020 [27]	Aged 4–18 years	RCTs, cohort, and quasi-experimental	School-based	School	BMI z-score, BMI, Overweight/Obesity Prevalence	BMI z-score, BMI, Overweight/Obesity Prevalence	None	Downs and Black checklist	Critically Low
Durão et al., 2023 [28]	Aged 2–18 years	RCTs, Interrupted time–series, prospective controlled studies	Policies or interventions that influence the school food environment	School	BMI Overweight/Obesity Prevalence	None	Overweight/Obesity Prevalence	Risk of Bias tool (Cochrane)	Moderate
Goncalves et al., 2021 [29]	Aged 10–19 years	Observational studies	Food environment in and around schools	School	BMI, Obesity Prevalence	Obesity Prevalence	None	The Joanna Briggs Institute	Moderate
Hayba et al., 2021 [51]	Aged 13–18 years, Racial/ethnic minorities	RCTs	Lifestyle factors, including nutrition and physical activity	Any	BMI, Overweight/Obesity Prevalence	None	BMI	Risk of Bias tool (Cochrane)	High
Hudak et al., 2019 [31]	Aged 2–18 years, participants in SNAP	Not described (mostly cross-sectional)	SNAP	Food Assistance Program	Overweight/Obesity Prevalence	None	None	Strength of Design framework	Critically Low
Kenney et al., 2023 [32]	Aged 2–18 years	Not described (studies with a comparison group)	Child and Adult Care Food Program	Food Assistance Program	Overweight/Obesity Prevalence	None	None	National Institutes of Health (NIH) quality assessment tool for observational cohort and cross-sectional studies	Critically Low
Kornet-van der Aa et al., 2017 [33]	Aged 12–18 years from disadvantaged backgrounds	RCTs, NRCTs	Any obesity prevention intervention	Any	BMI z-score, BMI, Overweight/Obesity Prevalence	None	None	Effective Public Health Practice Project	Moderate
Long et al., 2021 [34]	Aged <18 years	RCTs	Medical health education containing dietary contents	Any	BMI z-score, BMI	BMI z-score, BMI	None	Risk of Bias tool (Cochrane)	Critically Low
Morgan et al., 2020 [35]	Aged 2–18 years	RCTs, quasi-RCTs	Caregivers involved in at least one aspect of the intervention	Home and Family	BMI, Overweight/Obesity Prevalence	BMI, Overweight/Obesity Prevalence	None	Cochrane methods for cluster-RCTs	High
Nally et al., 2021 [36]	Aged 5–12 years	RCTs	School-based interventions	School	BMI z-score, BMI	BMI z-score, BMI	None	Risk of Bias tool (Cochrane)	Critically Low
Narzisi et al., 2020 [37]	Aged 0–5 years	RCTs	Lifestyle interventions to prevent obesity	Healthcare	Overweight Prevalence	No	No	JBI	Critically Low
Nury et al., 2021 [38]	Aged 4–18 years	Cluster-RCTs	Nutritional intervention strategies in the school setting	School	BMI z-score, BMI, Overweight/Obesity Prevalence	BMI z-score, BMI, Overweight/Obesity Prevalence	BMI z-score, BMI, Overweight/Obesity Prevalence	RoB2 tool (Cochrane)	High
Olstad et al., 2017 [39]	Aged 2–18 years from disadvantaged backgrounds	RCTs, quasi-experimental, controlled pre–post studies	Food Assistance Programs	Food Assistance Programs	BMI z-score, BMI	No	No	Effective Public Health Practice Project Quality	Moderate
Pineda et al., 2021 [40]	Aged ≤19 years	RCTs and Quasi-experimental studies	Interventions that focus on the school food environment	School	BMI z-score	BMI z-score	None	RoB2 ROBINS-I (Cochrane)	Critically Low
Qi et al., 2021 [41]	Aged 7–12 years	RCTs	School gardening	School	BMI z-score, BMI	BMI z-score, BMI	None	RoB2 (Cochrane)	Critically Low
Rochira et al., 2020 [42]	Aged 6–13 years	RCTs, quasi-experimental studies, observational studies	School gardening	School	BMI z-score, BMI percentile, BMI	BMI z-score, BMI Percentile, BMI	No	Cochrane Tool for Quality Assessment, STROBE	Critically Low
Salam et al., 2020 [43]	Aged 0–19 years	RCTs and Quasi-experimental studies	Lifestyle interventions, including dietary, physical activity, behavioral therapy, or any combination of these	Any	BMI z-score, Overweight/Obesity Prevalence, Cost-effectiveness	BMI z-score, Overweight/Obesity Prevalence	None	Risk of Bias tool (Cochrane)	Low
Seral-Cortes et al., 2021 [44]	Aged 6–12 years	RCTs	Health programs preventing obesity and T2DM with diet, physical activity, and behavioral support	Any	BMI z-score, BMI	BMI z-score, BMI	None	Risk of Bias tool (Cochrane)	Critically Low
Silva et al., 2022 [45]	Aged 10–19 years	RCTs and quasi-experimental studies	Computer-based nutrition interventions carried out in school	Electronic	BMI	BMI	BMI	Center for Reviews and Dissemination	Critically Low
Smit et al., 2023 [46]	Aged 6–12 years	RCTs and NRCTs	Primary school-based obesity prevention interventions	School	BMI z-score, BMI, Overweight/Obesity Prevalence	BMI z-score, BMI	BMI z-score, BMI, Overweight/Obesity Prevalence	RoB2 ROBINS-I (Cochrane)	High
Specchia et al., 2018 [47]	Aged <18 years	Not described	Multi-component, multi-level, or multi-setting	Any	Overweight/Obesity Prevalence	Overweight/Obesity Prevalence	None	Risk of Bias tool (Cochrane)	Low
Suleiman-Martos et al., 2021 [48]	Children or Adolescents	RCTs	Game-based interventions (gamification)	Electronic	BMI z-scores	Yes	No	Cochrane’s ROB	Critically Low
Tissot et al., 2021 [49]	Aged 10–19 years	RCTs and cluster-RCTs	Delivered in primary care	Healthcare	BMI z-scores, BMI percentiles	None	None	Risk of Bias tool (Cochrane)	Moderate
Whitehead et al., 2021 [50]	Aged ≤18 years	RCTs	Nurse-led interventions to prevent overweight or obesity	Healthcare	BMI, BMI-SDS	None	None	RoB2	Low

BMI—body mass index; NRCT—nonrandomized controlled trial; RCT—randomized controlled trial; RoB—risk of bias.

**Table 3:** Quantitative results of included systematic reviews examining the impact of pediatric obesity prevention interventions with nutrition.

Author, Year	Setting	Outcome	N Studies	N Participants	Effect Size Type	Effect Size	95% CI	CoE ^a^	SR Quality
Combined Age Groups									
Abdel Rahman et al., 2018 [20]	All settings	BMI z-score	3	3475	MD	−0.01	−0.05, 0.03	-	Critically Low
Long et al., 2021 [34]	All settings	BMI z-score	17	16,351	MD	−0.04	−0.06, −0.02	-	Critically Low
Salam et al., 2020 [43]	All settings	BMI z-score	32	33,039	MD	−0.12	−0.18, −0.06	Low	Low
Long et al., 2021 [34]	All settings	BMI	20	21,334	MD	−0.12	−0.20, −0.05	-	Critically Low
Salam et al., 2020 [43]	All settings	BMI	35	47,499	MR	−0.41	−0.60, −0.21	Low	Low
Salam et al., 2020 [43]	All settings	Overweight/Obesity Prevalence	12	NR	-	-	-	-	Low
Specchia et al., 2018 [47]	All settings	Overweight/Obesity Prevalence	11	137,058	MD (%)	−0.03	−0.04, −0.01	-	Low
Salam et al., 2020 [43]	All settings	Cost-effectiveness	6	NR	-	Four studies showed cost-efficacy, one study showed cost savings, and one was unclear.	-	-	Low
Ananthapavan et al., 2019 [21]	Community	BMI z-score	9	Unclear	MD	−0.07	−0.13, −0.01	-	Critically Low
Ananthapavan et al., 2019 [21]	Community	Cost-effectiveness	Modeling	Modeling	HALY, ICER, AUD/HALY gained	Probability of intervention being cost-effective was 95%	-	-	Critically Low
Suleiman-Martos et al., 2021 [48]	Electronic	BMI z-score	2	571	MD	−0.05	−0.21, 0.11	-	Critically Low
Silva et al., 2022 [45]	Electronic	BMI	3	3542	MD	−0.02	−0.18, 0.14	Moderate	Critically Low
Whitehead et al., 2021 [50]	Healthcare	BMI z-score	1	552	MD	−0.14	−0.26, −0.02	-	Low
Tissot et al., 2021 [49]	Healthcare	BMI	9	NR	-	Mixed findings	-	-	Moderate
Narzisi et al., 2020 [37]	Healthcare	Overweight Prevalence	1	NR	-	No difference between groups	-	-	Critically Low
Morgan et al., 2020 [35]	Home and Family	BMI	4	1861	SMD	0.05	−0.04, 0.15	-	High
Morgan et al., 2020 [35]	Home and Family	Overweight/Obesity Prevalence	3	1866	RR	1.02	0.89, 1.17	-	High
Abdel Rahman et al., 2018 [20]	School	BMI z-score	2	3384	MD	−0.04	−0.15, 0.06	-	Critically Low
Buchanan et al., 2023 [23]	School	BMI z-score	10	NR	Median (IQR)	−0.07	−0.19, −0.02	-	Critically Low
Dabravolskaj 2020 [27]	School 1	BMI z-score	9	17,105	MD	−0.016	−0.04, 0.01	-	Critically Low
Dabravolskaj 2020 [27]	School 3	BMI z-score	3	1,069,346	MD	−0.006	−0.02, 0.008	-	Critically Low
Dabravolskaj 2020 [27]	School 4	BMI z-score	2	1526	MD	0.05	−0.05, 0.15	-	Critically Low
Nury et al., 2021 [38]	School	BMI z-score	8	8174	MD	−0.09	−0.18, 0.00	Low	High
Pineda et al., 2021 [40]	School	BMI z-score	5	NR	MD	−0.12	−0.15, −0.10	-	Critically Low
Dabravolskaj 2020 [27]	School 1	BMI	8	15,018	MD	−0.26	−0.40, −0.12	-	Critically Low
Dabravolskaj 2020 [27]	School 2	BMI	1	320	MD	−0.33	−0.94, 0.28	-	Critically Low
Nury et al., 2021 [38]	School	BMI	10	12,067	MD	0.03	−0.10, 0.16	Low	High
Dabravolskaj 2020 [27]	School 2	BMI percentile	2	740	MD	−7.92	−16.53, 0.70	-	Critically Low
Durão et al., 2023 [28]	School	Overweight/Obesity Prevalence	3	67,841	-	Mixed Findings	-	Very Low	Moderate
Buchanan et al., 2023 [23]	School	Overweight/Obesity Prevalence	9	NR	Median (IQR) (%)	−2.5	−8.1, −1.6	-	Critically Low
Dabravolskaj 2020 [27]	School 1	Overweight/Obesity Prevalence	3	8848	OR	0.85	0.71, 1.01	-	Critically Low
Dabravolskaj 2020 [27]	School 3	Overweight/Obesity Prevalence	2	1,068,512	OR	0.96	0.86, 1.06	-	Critically Low
Dabravolskaj 2020 [27]	School 4	Overweight/Obesity Prevalence	1	1362	OR	1.21	0.95, 1.55	-	Critically Low
Goncalves et al., 2023 [29]	School 1	Overweight/Obesity Prevalence	5	88,530	OR	1.14	1.01, 1.28	-	Moderate
Goncalves et al., 2023 [29]	School 2	Overweight/Obesity Prevalence	4	80,864	OR	0.89	0.82, 0.96	-	Moderate
Goncalves et al., 2023 [29]	School 3	Overweight/Obesity Prevalence	3	10,377	OR	0.70	0.40, 1.22	-	Moderate
Nury et al., 2021 [38]	School	Overweight/Obesity Prevalence	3	901	OR	1.19	0.95, 1.49	Very Low	High
Ages 2–5 years									
Brown et al., 2019 [22]	All settings	BMI z-score	16	6261	MD	−0.07	−0.14, −0.01	Moderate	High
Brown et al., 2019 [22]	All settings	BMI	11	5536	MD	−0.11	−0.21, 0	Moderate	High
Brown et al., 2019 [22]	Community	BMI z-score	2	632	MD	−0.02	−0.13, 0.09	-	High
Brown et al., 2019 [22]	Community	BMI	1	75	MD	−0.59	−0.94, −0.24	-	High
Brown et al., 2019 [22]	Healthcare	BMI z-score	1	121	MD	−0.24	−0.26, −0.02	-	High
Brown et al., 2019 [22]	Home and Family	BMI z-score	3	595	MD	−0.13	−0.35, 0.09	-	High
Brown et al., 2019 [22]	Home and Family	BMI	2	778	MD	−0.33	−0.55, −0.1	-	High
Brown et al., 2019 [22]	School/Childcare	BMI z-score	10	4913	MD	−0.04	−0.09, 0.01	-	High
Brown et al., 2019 [22]	School/Childcare	BMI	9	4683	MD	−0.05	−0.14, 0.05	-	High
Ages 6–12 years									
Brown et al., 2019 [22]	All settings	BMI z-score	20	24,043	MD	−0.05	−0.1, −0.01	Low	High
Seral-Cortes 2021 [44]	All settings	BMI z-score	16	17,989	SMD	−0.06	−0.12, 0.01	-	Critically Low
Brown et al., 2019 [22]	All settings	BMI	25	19,498	MD	−0.05	−0.11, 0.01	Low	High
Seral-Cortes 2021 [44]	All settings	BMI	30	13,128	SMD	−0.01	−0.14, 0.13	-	Critically Low
Ananthapavan et al., 2019 [21]	Community	BMI z-score	5	NR	MD	−0.12	−0.23, −0.01	-	Critically Low
Brown et al., 2019 [22]	Community	BMI z-score	4	657	MD	−0.04	−0.39, 0.31	-	High
Brown et al., 2019 [22]	Community	BMI	9	742	MD	−0.08	−0.29, 0.23	-	High
Abdel Rahman et al., 2018 [20]	Home and Family	BMI z-score	1	93	MD	0.02	−0.02, 0.06	-	Critically Low
Brown et al., 2019 [22]	Home and Family	BMI z-score	1	134	MD	0.03	−0.04, 0.1	-	High
Brown et al., 2019 [22]	School	BMI z-score	15	22,879	MD	−0.05	−0.1, 0.01	-	High
Nally et al., 2021 [36]	School	BMI z-score	20	16,787	SMD	−0.05	−0.08, −0.02	-	Critically Low
Qi et al., 2021 [41]	School	BMI z-score	5	4285	MD	−0.12	−0.26, 0.02	-	Critically Low
Rochira et al., 2020 [42]	School	BMI z-score	4	1996	MD	−0.09	−0.19, 0.01	-	Critically Low
Smit et al., 2023 [46]	School	BMI z-score	3	2730	MD	−0.08	−0.20, 0.05	Very Low	High
Brown et al., 2019 [22]	School	BMI	16	18,488	MD	−0.04	−0.1, 0.02	-	High
Cerrato-Carretero et al. 2021 [25]	School	BMI	11	17,446	SMD	−0.00	−0.05, 0.04	-	Critically Low
Nally et al., 2021 [36]	School	BMI	21	14,101	MD	−0.39	−0.47, −0.30	-	Critically Low
Qi et al., 2021 [41]	School	BMI	5	3991	MD	−0.49	−1.63, 0.65	-	Critically Low
Rochira et al., 2020 [42]	School	BMI	2	188	MD	0.13	−0.94, 1.20	-	Critically Low
Smit et al., 2023 [46]	School	BMI	6	5453	MD	0.06	−0.38, 0.50	Very Low	High
Rochira et al., 2020 [42]	School	BMI percentile	4	4593	MD	−1.37	−2.38, 0.37	-	Critically Low
Smit et al., 2023 [46]	School	Overweight/Obesity Prevalence	9	7059	-	-	-	Very Low	High
Ages 13–17 years									
Brown et al., 2019 [22]	All settings	BMI z-score	6	16,543	MD	0.01	−0.05, 0.07	Low	High
Brown et al., 2019 [22]	All settings	BMI	8	16,583	MD	−0.02	−0.1, 0.05	Low	High
Hayba 2021 [51]	All settings	BMI	7	2763	-	No difference between groups	-	Low	High
Kornet-van der Aa 2017 [33]	All settings	Overweight/Obesity Prevalence	1	235	MD (%)	Percent with overweight/obesity decreased in intervention	-	-	Moderate
Ananthapavan et al., 2019 [21]	Community	BMI z-score	4	NR	MD	−0.02	−0.07, 0.03	-	
Brown et al., 2019 [22]	Home and Family	BMI z-score	1	75	MD	0.06	−0.13, 0.26	-	High
Brown et al., 2019 [22]	School	BMI z-score	5	16,173	MD	0	−0.06, 0.06	-	High
Brown et al., 2019 [22]	School	BMI	8	16,347	MD	−0.02	−0.1, 0.05	-	High

BMI—body mass index; CoE—certainty of evidence; CI—confidence interval; HALY—health-adjusted life years; ICER—incremental cost-effectiveness ratio; IQR—interquartile range; MD—mean difference; N—number; NR—not reported; SMD—standardized mean difference; SR—systematic review. ^a^ As determined by systematic review authors.

**Table 4:** Summary of findings table describing systematic reviews examining the effects of nutrition interventions for pediatric obesity prevention in all settings in individuals 2–17 years old.

Outcome No. of Participants (Studies)	Anticipated Absolute Effects (95% CI)	Certainty	What Happens
	Difference		
All Age Groups			
BMI z-score [43] No. of participants: 33,039 (32 RCTs)	MD 0.12 lower (0.18 lower to 0.06 lower)	⨁⨁◯◯ LOW ^a^	In pediatric individuals, diet and physical activity interventions may reduce BMI z-score.
BMI [43] No. of participants: 47,499 (35 RCTs)	MD 0.41 kg/m^2^ lower (0.6 lower to 0.21 lower)	⨁⨁◯◯ LOW ^a^	In pediatric individuals, diet and physical activity interventions may reduce BMI.
Overweight/Obesity Prevalence [47] No. of participants: 137,058 (11 RCTs)	MD 0.03% lower (0.04 lower to 0.01 lower)	⨁⨁◯◯ LOW ^b^	In pediatric individuals, highly integrated overweight/obesity prevention programs may reduce the prevalence of overweight/obesity.
Cost-effectiveness [43] No. of participants: Unclear (6 RCTs)	NR	⨁⨁◯◯ LOW ^c,d^	In pediatric individuals, diet and physical activity interventions may be cost-effective.
Ages 0–5 years			
BMI z-score [22] No. of participants: 6261 (16 RCTs)	MD 0.07 lower (0.14 lower to 0.01 lower)	⨁⨁⨁◯ MODERATE ^a^	Diet and physical activity interventions combined likely reduce BMI z-scores in individuals 0–5 years old.
BMI [22] No. of participants: 5536 (11 RCTs)	MD 0.11 kg/m^2^ lower (0.21 lower to 0)	⨁⨁⨁◯ MODERATE ^a^	Diet and physical activity interventions combined likely result in little to no difference in BMI in individuals 0–5 years old.
Ages 6–12 years			
BMI z-score [22] No. of participants: 24,043 (20 RCTs)	MD 0.05 lower (0.11 lower to 0.01 lower)	⨁⨁◯◯ LOW ^a^	Diet and physical activity interventions combined may reduce BMI z-score in individuals 6–12 years old.
BMI [22] No. of participants: 19,498 (25 RCTs)	MD 0.05 kg/m^2^ lower (0.11 lower to 0.01 higher)	⨁⨁◯◯ LOW ^b^	Diet and physical activity interventions combined may result in little to no difference in BMI in individuals 6–12 years old.
Ages 13–17 years			
BMI z-score [22] No. of participants: 16,453 (6 RCTs)	MD 0.01 higher (0.05 lower to 0.07 higher)	⨁⨁◯◯ LOW ^a^	Combined diet and physical activity interventions may result in little to no difference in BMI z-score in individuals 13–18 years old.
BMI [22] No. of participants: 16,583 (8 RCTs)	MD 0.02 lower (0.1 lower to 0.05 higher)	⨁⨁◯◯ LOW ^a,b^	Combined diet and physical activity interventions may result in little to no difference in BMI in individuals 13–18 years old.
Overweight and Obesity Prevalence [33] No. of participants: 235 (1 RCT)	NR	⨁⨁⨁◯ VERY LOW ^b,c,d^	In adolescents 12–18 years old, one RCT reported that obesity prevention interventions may reduce the percentage of participants with overweight or obesity.

BMI—body mass index; CI—confidence interval; MD—mean difference; NR—not reported; RCT—randomized controlled trial. Explanations: ^a^ High heterogeneity. ^b^ Risk of bias in included studies. ^c^ Small sample size or wide confidence interval. ^d^ No effect size described.

## Supplementary Materials

The following supporting information can be downloaded at: https://www.mdpi.com/article/10.3390/nu15245097/s1.
