# Supplementary material for: Nutrition Interventions for Pediatric Obesity Prevention: An Umbrella Review of Systematic Reviews
Source: Nutrients. 2023 Dec 13;15(24):5097. doi: 10.3390/nu15245097 (PMC10745722; doi:10.3390/nu15245097)
Supplement: Supplementary file 1 [file nutrients-15-05097-s001.zip › nutrients-2737881-supplementary.pdf]

Peds Obesity prevention Search June 8, 2023

Table S1. Summary of Entries Obtained from Databases Search on June 8, 2023 (Updated from 2021)

MEDLINE	1274
CINAHL	665
Food Science Source	425
CDSR	16
Total	2380
Duplicates	650+170=820
Unique Citations	1560

Table S2. MEDLINE search for Systematic Reviews Examining Impact of Pediatric Obesity Interventions on June 8, 2023

S49	S37 AND S46	**Limiters** - Date of Publication: 20210101-20231231<br>**Narrow by Language:** - english<br>**Search modes** - Boolean/Phrase	View Results (1,274)<br>View Details<br>Edit
S48	S37 AND S46	**Limiters** - Date of Publication: 20210101-20231231<br>**Search modes** - Boolean/Phrase	View Results (1,289)<br>View Details<br>Edit
S47	S37 AND S46	**Search modes** - Boolean/Phrase	View Results (5,018)<br>View Details<br>Edit
S46	S38 OR S39 OR S40 OR S41 OR S42 OR S43 OR S44 OR S45	**Search modes** - Boolean/Phrase	View Results (640,460)<br>View Details<br>Edit
S45	AB "risk of bias"	**Search modes** - Boolean/Phrase	View Results (40,379)<br>View Details<br>Edit
S44	AB (risk of bias or (newcastle N2 ottawa))	**Search modes** - Boolean/Phrase	View Results (48,981)<br>View Details

			Edit
S43	AB (data extraction or pubmed or medline or cinahl or scopus or embase)	**Search modes** - Boolean/Phrase	View Results (334,794)<br>View Details<br>Edit
S42	SO cochrane OR systematic review*	**Search modes** - Boolean/Phrase	View Results (323,051)<br>View Details<br>Edit
S41	(TI (metaanaly* or meta-analy*) OR AB (metaanaly* or meta-analy*)) OR (TI (evidence N2 synthesi*) OR AB (evidence N2 synthesi*)) OR (TI (evidence N2 review) OR AB (evidence N2 review))	**Search modes** - Boolean/Phrase	View Results (331,039)<br>View Details<br>Edit
S40	TI ((systematic or scoping or meta- or overview* or review) N2 review*) or AB ((systematic or scoping or meta- or overview*) N2 review*)	**Search modes** - Boolean/Phrase	View Results (338,079)<br>View Details<br>Edit
S39	(MH "Network Meta-Analysis") or MH "meta-analysis"	**Search modes** - Boolean/Phrase	View Results (183,087)<br>View Details<br>Edit
S38	(MH "Systematic Review") OR (MH "Systematic Reviews as Topic")	**Search modes** - Boolean/Phrase	View Results (239,616)<br>View Details<br>Edit
S37	(S28 AND S35) OR S36	**Search modes** - Boolean/Phrase	View Results (140,605)<br>View Details<br>Edit
S36	(MH "Child Nutrition Sciences")	**Search modes** - Boolean/Phrase	View Results (1,178)<br>View Details<br>Edit
S35	S29 OR S30 OR S31 OR S32 OR S33 OR S34	**Search modes** - Boolean/Phrase	View Results (3,972,961)<br>View Details

			Edit
S34	TI paediatric* or AB paediatric*	**Search modes** - Boolean/Phrase	View Results (79,333)<br>View Details<br>Edit
S33	TI (pediatric*) OR AB pediatric*	**Search modes** - Boolean/Phrase	View Results (370,651)<br>View Details<br>Edit
S32	MW (pediatric*)	**Search modes** - Boolean/Phrase	View Results (113,453)<br>View Details<br>Edit
S31	TI (preschool* or pre-school* or highschool* or middle school* or kindergar* or kintergar* or play school or play schools or playschool* or "after school") OR AB (preschool* or pre- school* or highschool* or middle school* or kindergar* or kintergar* or play school or play schools or playschool* or "after school")	**Search modes** - Boolean/Phrase	View Results (60,654)<br>View Details<br>Edit
S30	(TI (child or childs or children or child's or childhood or teen* or adolescent* or adolescence)) OR (AB (child or childs or children or child's or childhood or teen* or adolescent* or adolescence))	**Search modes** - Boolean/Phrase	View Results (1,800,396)<br>View Details<br>Edit
S29	(MH "Child+") OR (MH "Adolescent")	**Search modes** - Boolean/Phrase	View Results (3,355,090)<br>View Details<br>Edit
S28	S1 OR S2 OR S3 OR S4 OR S5 OR S6 OR S7 OR S8 OR S9 OR S10 OR S11 OR S12 OR S13 OR S14 OR S15 OR S16 OR S17 OR S18 OR S19 OR S20 OR S21 OR	**Search modes** - Boolean/Phrase	View Results (940,709)<br>View Details<br>Edit

	S22 OR S23 OR S24 OR S25 OR S26 OR S27		
S27	TI (nutrition N2 (counsel* or advice or advis*)) OR AB (nutrition N2 (counsel* or advice or advis*))	**Search modes** - Boolean/Phrase	View Results (2,239)<br>View Details<br>Edit
S26	TI (nutrition N2 (therapy or therapies)) OR AB (nutrition N2 (therapy or therapies))	**Search modes** - Boolean/Phrase	View Results (3,457)<br>View Details<br>Edit
S25	TI (nutrition N2 (guide* OR guidanc* or guiding)) OR AB (nutrition N2 (guide* OR guidanc* or guiding))	**Search modes** - Boolean/Phrase	View Results (2,208)<br>View Details<br>Edit
S24	TI (food N2 (guide* OR guidanc* or guiding)) OR AB (food N2 (guide* OR guidanc* or guiding))	**Search modes** - Boolean/Phrase	View Results (2,511)<br>View Details<br>Edit
S23	MH "Food+" AND MH "Decision Making"	**Search modes** - Boolean/Phrase	View Results (617)<br>View Details<br>Edit
S22	MH "Food+" AND MH "Choice Behavior"	**Search modes** - Boolean/Phrase	View Results (2,346)<br>View Details<br>Edit
S21	TI (healthy meal*) or AB (healthy meal*)	**Search modes** - Boolean/Phrase	View Results (2,066)<br>View Details<br>Edit
S20	TI ((healthy or poor) N2 eating) OR AB ((healthy or poor) N2 eating)	**Search modes** - Boolean/Phrase	View Results (11,318)<br>View Details<br>Edit
S19	TI (("soft drink" or "soft drinks" or ((sugar sweetened N2 (food or foods or beverage* or drink or drinks)) N2 (consum* or intake)) OR AB (("soft drink" or "soft	**Search modes** - Boolean/Phrase	View Results (6,473)<br>View Details<br>Edit

	drinks" or ((sugar sweetened N2 (food or foods or beverage* or drink or drinks)) N2 (consum* or intake))		
S18	TI ("lunch program" or "lunch programs" or "lunch programme" or "lunch programmes" or "school lunch*") OR AB ("lunch program" or "lunch programs" or "lunch programme" or "lunch programmes" or "school lunch*")	**Search modes** - Boolean/Phrase	View Results (1,171)<br>View Details<br>Edit
S17	TI (Recommended Dietary Allowance*) OR AB (Recommended Dietary Allowance*	**Search modes** - Boolean/Phrase	View Results (2,274)<br>View Details<br>Edit
S16	TI (recommended daily allowance) OR AB (recommended daily allowance)	**Search modes** - Boolean/Phrase	View Results (991)<br>View Details<br>Edit
S15	TI ((nutrition or food or foods) N2 (policy or policies or program*)) OR AB ((nutrition or food or foods) N2 (policy or policies or program*))	**Search modes** - Boolean/Phrase	View Results (13,006)<br>View Details<br>Edit
S14	(MH "Recommended Dietary Allowances") OR (MH "Nutrition Policy") OR (MH "Nutritional Sciences")	**Search modes** - Boolean/Phrase	View Results (24,204)<br>View Details<br>Edit
S13	MH "Nutrition Surveys" OR MH "Diet Surveys"	**Search modes** - Boolean/Phrase	View Results (33,001)<br>View Details<br>Edit
S12	TI (food N2 Legislation) OR AB (food N2 Legislation)	**Search modes** - Boolean/Phrase	View Results (379)<br>View Details<br>Edit

S11	TI ("Supplemental Nutrition Assistance Program" or "food stamps" or "food aid" or "snap program*" or "food assistance" OR "Special Supplemental Nutrition Program for Women" or "WIC program*") OR AB ("Supplemental Nutrition Assistance Program" or "food stamps" or "food stamps" or "food aid" or "snap program*" or "food assistance" OR "Special Supplemental Nutrition Program for Women" or "WIC program*"))	**Search modes** - Boolean/Phrase	View Results (2,830)<br>View Details<br>Edit
S10	(MH "Legislation, Food") OR (MH "Food Assistance")	**Search modes** - Boolean/Phrase	View Results (4,286)<br>View Details<br>Edit
S9	MH "Diet Therapy+" OR MH "Nutrition Therapy"	**Search modes** - Boolean/Phrase	View Results (65,123)<br>View Details<br>Edit
S8	TI (feeding behavior* or feeding behaviour*) or AB (feeding behavior* or feeding behaviour*)	**Search modes** - Boolean/Phrase	View Results (16,527)<br>View Details<br>Edit
S7	TI ((food or eating) N2 (behavior* or behaviour* or habit or habits or preference*)) OR AB ((food or eating) N2 (behavior* or behaviour* or habit or habits or preference*))	**Search modes** - Boolean/Phrase	View Results (36,299)<br>View Details<br>Edit
S6	(MH "Fast Foods") OR TI ("fast food*" OR "junk food*" or "processed food*" or convenience food* or "ready to eat" or "ready prepared food*") OR AB ("fast food*" OR "junk food*" or "processed food*" or convenience	**Search modes** - Boolean/Phrase	View Results (14,938)<br>View Details<br>Edit

	food* or "ready to eat" or "ready prepared food*")		
S5	MH "Food Preferences" OR MH "Food Fussiness"	**Search modes** - Boolean/Phrase	View Results (16,314)<br>View Details<br>Edit
S4	MH "Feeding Behavior"	**Search modes** - Boolean/Phrase	View Results (92,470)<br>View Details<br>Edit
S3	TI (energy intake or (calori* N2 (intake or restriction*)) OR portion size* or serving size*) OR AB (energy intake or (calori* N2 (intake or restriction*)) OR portion size* or serving size*)	**Search modes** - Boolean/Phrase	View Results (58,456)<br>View Details<br>Edit
S2	TI (diet*) or AB (diet*)	**Search modes** - Boolean/Phrase	View Results (666,704)<br>View Details<br>Edit
S1	(MH "Diet+") OR ((MH "Nutritionists") OR (MH "Dietetics"))	**Search modes** - Boolean/Phrase	View Results (334,659)<br>View Details<br>Edit

Table S3. CINAHL search for Systematic Reviews Examining Impact of Pediatric Obesity Interventions on June 8, 2023

S49	S37 AND S46	Limiters - Published Date: 20210101-20231231<br>Search modes - Boolean/Phrase	Interface - EBSCO Discovery Service<br>Search Screen - Advanced Search<br>Database - Discovery Service for Academy of Nutrition and Dietetics.	665
S48	S37 AND S46	Limiters - Published Date: 20210101-20231231<br>Search modes - Boolean/Phrase	Interface - EBSCO Discovery Service<br>Search Screen - Advanced Search<br>Database - Discovery Service for Academy of Nutrition and Dietetics.	679
S47	S37 AND S46	Search modes - Boolean/Phrase	Interface - EBSCO Discovery Service<br>Search Screen - Advanced Search<br>Database - Discovery Service for Academy of Nutrition and Dietetics.	3,053
S46	S38 OR S39 OR S40 OR S41 OR S42 OR S43 OR S44 OR S45	Search modes - Boolean/Phrase	Interface - EBSCO Discovery Service<br>Search Screen - Advanced Search<br>Database - Discovery Service for Academy of Nutrition and Dietetics.	292,327
S45	AB "risk of bias"	Search modes - Boolean/Phrase	Interface - EBSCO Discovery Service<br>Search Screen - Advanced Search<br>Database - Discovery Service for Academy of Nutrition and Dietetics.	15,707
S44	AB (risk of bias or (newcastle N2 ottawa))	Search modes - Boolean/Phrase	Interface - EBSCO Discovery Service<br>Search Screen - Advanced Search<br>Database - Discovery Service for Academy of Nutrition and Dietetics.	20,147
S43	AB (data extraction or pubmed or medline or cinahl or scopus or embase)	Search modes - Boolean/Phrase	Interface - EBSCO Discovery Service<br>Search Screen - Advanced Search<br>Database - Discovery Service for Academy of Nutrition and Dietetics.	125,397
S42	SO cochrane OR systematic review*	Search modes - Boolean/Phrase	Interface - EBSCO Discovery Service<br>Search Screen - Advanced Search<br>Database - Discovery Service for Academy of Nutrition and Dietetics.	196,840

S41	TI (metaanaly* or meta-analy*) OR AB (metaanaly* or meta-analy*)) OR (TI (evidence N2 synthesi*) OR AB (evidence N2 synthesi*)) OR (TI (evidence N2 review) OR AB (evidence N2 review))	Search modes - Boolean/Phrase	Interface - EBSCO Discovery Service Search Screen - Advanced Search Database - Discovery Service for Academy of Nutrition and Dietetics.	130,723
S40	TI ((systematic or scoping or meta- or overview* or review) N2 review*) or AB ((systematic or scoping or meta- or overview*) N2 review*)	Search modes - Boolean/Phrase	Interface - EBSCO Discovery Service Search Screen - Advanced Search Database - Discovery Service for Academy of Nutrition and Dietetics.	162,652
S39	(MH "Meta Analysis") OR (MH "Meta Synthesis")	Search modes - Boolean/Phrase	Interface - EBSCO Discovery Service Search Screen - Advanced Search Database - Discovery Service for Academy of Nutrition and Dietetics.	71,212
S38	(MH "Systematic Review") OR (MH "Scoping Review") OR (MH "Cochrane Library")	Search modes - Boolean/Phrase	Interface - EBSCO Discovery Service Search Screen - Advanced Search Database - Discovery Service for Academy of Nutrition and Dietetics.	133,396
S37	(S28 AND S35) OR S36	Search modes - Boolean/Phrase	Interface - EBSCO Discovery Service Search Screen - Advanced Search Database - Discovery Service for Academy of Nutrition and Dietetics.	65,772
S36	(MH "Adolescent Nutrition") OR (MH "Adolescent Nutritional Physiology") OR (MH "Child Nutritional Physiology") OR (MH "Child Nutrition")	Search modes - Boolean/Phrase	Interface - EBSCO Discovery Service Search Screen - Advanced Search Database - Discovery Service for Academy of Nutrition and Dietetics.	10,112
S35	S29 OR S30 OR S31 OR S32 OR S33 OR S34	Search modes - Boolean/Phrase	Interface - EBSCO Discovery Service Search Screen - Advanced Search Database - Discovery Service for Academy of Nutrition and Dietetics.	1,210,595
S34	TI paediatric* or AB paediatric*	Search modes - Boolean/Phrase	Interface - EBSCO Discovery Service Search Screen - Advanced Search	30,992

			Database - Discovery Service for Academy of Nutrition and Dietetics.	
S33	TI (pediatric*) OR AB pediatric*	Search modes - Boolean/Phrase	Interface - EBSCO Discovery Service Search Screen - Advanced Search Database - Discovery Service for Academy of Nutrition and Dietetics.	142,927
S32	MW (pediatric*)	Search modes - Boolean/Phrase	Interface - EBSCO Discovery Service Search Screen - Advanced Search Database - Discovery Service for Academy of Nutrition and Dietetics.	100,858
S31	TI (preschool* or pre-school* or highschool* or middle school* or kindergar* or kintergar* or play school or play schools or playschool* or "after school") OR AB (preschool* or pre- school* or highschool* or middle school* or kindergar* or kintergar* or play school or play schools or playschool* or "after school")	Search modes - Boolean/Phrase	Interface - EBSCO Discovery Service Search Screen - Advanced Search Database - Discovery Service for Academy of Nutrition and Dietetics.	29,880
S30	(TI (child or childs or children or child's or childhood or teen* or adolescent* or adolescence)) OR (AB (child or childs or children or child's or childhood or teen* or adolescent* or adolescence))	Search modes - Boolean/Phrase	Interface - EBSCO Discovery Service Search Screen - Advanced Search Database - Discovery Service for Academy of Nutrition and Dietetics.	683,107
S29	(MH "Child") OR (MH "Adolescence+")	Search modes - Boolean/Phrase	Interface - EBSCO Discovery Service Search Screen - Advanced Search Database - Discovery Service for Academy of Nutrition and Dietetics.	885,263
S28	S1 OR S2 OR S3 OR S4 OR S6 OR S7 OR S8 OR S9 OR S10 OR S11 OR S12 OR S13 OR S15 OR S16 OR S17 OR S18 OR S19 OR	Search modes - Boolean/Phrase	Interface - EBSCO Discovery Service Search Screen - Advanced Search Database - Discovery Service for Academy of Nutrition and Dietetics.	278,983

	S20 OR S21 OR S23 OR S24 OR S25 OR S26 OR S27			
S27	TI (nutrition N2 (counsel* or advice or advis*)) OR AB (nutrition N2 (counsel* or advice or advis*))	Search modes - Boolean/Phrase	Interface - EBSCO Discovery Service Search Screen - Advanced Search Database - Discovery Service for Academy of Nutrition and Dietetics.	1,421
S26	TI (nutrition N2 (therapy or therapies)) OR AB (nutrition N2 (therapy or therapies))	Search modes - Boolean/Phrase	Interface - EBSCO Discovery Service Search Screen - Advanced Search Database - Discovery Service for Academy of Nutrition and Dietetics.	1,995
S25	TI (nutrition N2 (guide* OR guidanc* or guiding)) OR AB (nutrition N2 (guide* OR guidanc* or guiding))	Search modes - Boolean/Phrase	Interface - EBSCO Discovery Service Search Screen - Advanced Search Database - Discovery Service for Academy of Nutrition and Dietetics.	1,538
S24	TI (food N2 (guide* OR guidanc* or guiding)) OR AB (food N2 (guide* OR guidanc* or guiding))	Search modes - Boolean/Phrase	Interface - EBSCO Discovery Service Search Screen - Advanced Search Database - Discovery Service for Academy of Nutrition and Dietetics.	1,509
S23	MH "Food+" AND ((MH "Decision Making+") OR (MH "Decision Making, Patient"))	Search modes - Boolean/Phrase	Interface - EBSCO Discovery Service Search Screen - Advanced Search Database - Discovery Service for Academy of Nutrition and Dietetics.	2,054
S22	MH "Food+" AND MH "Choice Behavior"	Search modes - Boolean/Phrase	Interface - EBSCO Discovery Service Search Screen - Advanced Search Database - Discovery Service for Academy of Nutrition and Dietetics.	0
S21	TI (healthy meal*) or AB (healthy meal*)	Search modes - Boolean/Phrase	Interface - EBSCO Discovery Service Search Screen - Advanced Search Database - Discovery Service for Academy of Nutrition and Dietetics.	1,116
S20	TI ((healthy or poor) N2 eating) OR AB ((healthy or poor) N2 eating)	Search modes - Boolean/Phrase	Interface - EBSCO Discovery Service Search Screen - Advanced Search Database - Discovery Service for Academy of Nutrition and Dietetics.	8,661

S19	TI (("soft drink" or "soft drinks" or ((sugar sweetened N2 (food or foods or beverage* or drink or drinks)) N2 (consum* or intake)) OR AB (("soft drink" or "soft drinks" or ((sugar sweetened N2 (food or foods or beverage* or drink or drinks)) N2 (consum* or intake))	Search modes - Boolean/Phrase	Interface - EBSCO Discovery Service Search Screen - Advanced Search Database - Discovery Service for Academy of Nutrition and Dietetics.	3,189
S18	TI ("lunch program" or "lunch programs" or "lunch programme" or "lunch programmes" or "school lunch*") OR AB ("lunch program" or "lunch programs" or "lunch programme" or "lunch programmes" or "school lunch*")	Search modes - Boolean/Phrase	Interface - EBSCO Discovery Service Search Screen - Advanced Search Database - Discovery Service for Academy of Nutrition and Dietetics.	851
S17	TI (Recommended Dietary Allowance*) OR AB (Recommended Dietary Allowance*)	Search modes - Boolean/Phrase	Interface - EBSCO Discovery Service Search Screen - Advanced Search Database - Discovery Service for Academy of Nutrition and Dietetics.	642
S16	TI (recommended daily allowance) OR AB (recommended daily allowance)	Search modes - Boolean/Phrase	Interface - EBSCO Discovery Service Search Screen - Advanced Search Database - Discovery Service for Academy of Nutrition and Dietetics.	277
S15	TI ((nutrition or food or foods) N2 (policy or policies or program*)) OR AB ((nutrition or food or foods) N2 (policy or policies or program*))	Search modes - Boolean/Phrase	Interface - EBSCO Discovery Service Search Screen - Advanced Search Database - Discovery Service for Academy of Nutrition and Dietetics.	7,744
S14	(MH "Recommended Dietary Allowances") OR (MH "Nutrition Policy") OR (MH "Nutritional Sciences")	Search modes - Boolean/Phrase	Interface - EBSCO Discovery Service Search Screen - Advanced Search Database - Discovery Service for Academy of Nutrition and Dietetics.	5,022
S13	(MH "Food Guide Pyramid") OR (MH "MyPlate") OR (MH	Search modes - Boolean/Phrase	Interface - EBSCO Discovery Service Search Screen - Advanced Search	12,022

	"Nutritional Requirements") OR (MH "Dietary Reference Intakes") OR (MH "Nutritional Support Team") OR (MH "Nutrition Services")		Database - Discovery Service for Academy of Nutrition and Dietetics.	
S12	TI (food N2 Legislation) OR AB (food N2 Legislation)	Search modes - Boolean/Phrase	Interface - EBSCO Discovery Service Search Screen - Advanced Search Database - Discovery Service for Academy of Nutrition and Dietetics.	74
S11	TI ("Supplemental Nutrition Assistance Program" or "food stamps" or "food aid" or "snap program*" or "food assistance" OR "Special Supplemental Nutrition Program for Women" or "WIC program*") OR AB ("Supplemental Nutrition Assistance Program" or "food stamps" or "food stamps" or "food aid" or "snap program*" or "food assistance" OR "Special Supplemental Nutrition Program for Women" or "WIC program*"))	Search modes - Boolean/Phrase	Interface - EBSCO Discovery Service Search Screen - Advanced Search Database - Discovery Service for Academy of Nutrition and Dietetics.	2,079
S10	(MH "Food Assistance")	Search modes - Boolean/Phrase	Interface - EBSCO Discovery Service Search Screen - Advanced Search Database - Discovery Service for Academy of Nutrition and Dietetics.	1,914
S9	MH "Diet Therapy+"	Search modes - Boolean/Phrase	Interface - EBSCO Discovery Service Search Screen - Advanced Search Database - Discovery Service for Academy of Nutrition and Dietetics.	36,316
S8	TI (feeding behavior* or feeding behaviour*) or AB (feeding behavior* or feeding behaviour*)	Search modes - Boolean/Phrase	Interface - EBSCO Discovery Service Search Screen - Advanced Search Database - Discovery Service for Academy of Nutrition and Dietetics.	1,918

S7	TI ((food or eating) N2 (behavior* or behaviour* or habit or habits or preference*)) OR AB ((food or eating) N2 (behavior* or behaviour* or habit or habits or preference*))	Search modes - Boolean/Phrase	Interface - EBSCO Discovery Service<br>Search Screen - Advanced Search<br>Database - Discovery Service for Academy of Nutrition and Dietetics.	15,748
S6	(MH "Fast Foods") OR TI ("fast food*" OR "junk food*" or "processed food*" or convenience food* or "ready to eat" or "ready prepared food*") OR AB ("fast food*" OR "junk food*" or "processed food*" or convenience food* or "ready to eat" or "ready prepared food*")	Search modes - Boolean/Phrase	Interface - EBSCO Discovery Service<br>Search Screen - Advanced Search<br>Database - Discovery Service for Academy of Nutrition and Dietetics.	6,729
S5	MH "Food Preferences" OR MH "Food Fussiness"	Search modes - Boolean/Phrase	Interface - EBSCO Discovery Service<br>Search Screen - Advanced Search<br>Database - Discovery Service for Academy of Nutrition and Dietetics.	7,684
S4	(MH "Eating Behavior+")	Search modes - Boolean/Phrase	Interface - EBSCO Discovery Service<br>Search Screen - Advanced Search<br>Database - Discovery Service for Academy of Nutrition and Dietetics.	49,680
S3	TI (energy intake or (calori* N2 (intake or restriction*)) OR portion size* or serving size*) OR AB (energy intake or (calori* N2 (intake or restriction*)) OR portion size* or serving size*)	Search modes - Boolean/Phrase	Interface - EBSCO Discovery Service<br>Search Screen - Advanced Search<br>Database - Discovery Service for Academy of Nutrition and Dietetics.	18,582
S2	TI (diet*) or AB (diet*)	Search modes - Boolean/Phrase	Interface - EBSCO Discovery Service<br>Search Screen - Advanced Search<br>Database - Discovery Service for Academy of Nutrition and Dietetics.	158,255
S1	(MH "Diet+") OR (MH "Dietitians") OR (MH "Dietetics")) OR (MH	Search modes - Boolean/Phrase	Interface - EBSCO Discovery Service<br>Search Screen - Advanced Search	146,212

	"Nutritional Support Team") OR (MH "Nutrition Services")		Database - Discovery Service for Academy of Nutrition and Dietetics.	

Table S4. Food Science Source search for Systematic Reviews Examining Impact of Pediatric Obesity Interventions on June 8, 2023

S39	(S24 OR S25) AND S29 AND S36	**Limiters** - Publication Date: 20210101-20231231<br>**Narrow by Language:** - english<br>**Search modes** - Boolean/Phrase	View Results (425)<br>View Details<br>Edit
S38	(S24 OR S25) AND S29 AND S36	**Limiters** - Publication Date: 20210101-20231231<br>**Search modes** - Boolean/Phrase	View Results (428)<br>View Details<br>Edit
S37	(S24 OR S25) AND S29 AND S36	**Search modes** - Boolean/Phrase	View Results (1,498)<br>View Details<br>Edit
S36	S30 OR S31 OR S32 OR S33 OR S34 OR S35	**Search modes** - Boolean/Phrase	View Results (51,155)<br>View Details<br>Edit
S35	AB (risk of bias or (newcastle N2 ottawa))	**Search modes** - Boolean/Phrase	View Results (3,724)<br>View Details<br>Edit
S34	AB (data extraction or pubmed or medline or cinahl or scopus or embase)	**Search modes** - Boolean/Phrase	View Results (20,607)<br>View Details<br>Edit
S33	SO cochrane OR SO systematic review*	**Search modes** - Boolean/Phrase	View Results (0)<br>View Details<br>Edit
S32	TI (metaanaly* or meta-analy*) OR AB (metaanaly* or meta-analy*)) OR KW (metaanaly* or meta-analy*) OR (TI (evidence N2 synthesi*) OR AB (evidence N2 synthesi*)) OR (TI (evidence N2 review) OR AB (evidence N2 review)) OR KW (evidence N2 synthesi*) OR AB (evidence N2 synthesi*)	**Search modes** - Boolean/Phrase	View Results (27,581)<br>View Details<br>Edit

S31	(TI ((systematic or scoping or meta- or overview* or review) N2 review*) or AB ((systematic or scoping or meta- or overview*) N2 review*))	**Search modes** - Boolean/Phrase	**View Results** (30,691)<br>**View Details**<br>**Edit**
S30	((SU "Systematic Review") OR (SU "Scoping Review")) OR ((SU "Meta Analysis") OR (SU "Meta Synthesis"))	**Search modes** - Boolean/Phrase	**View Results** (12,069)<br>**View Details**<br>**Edit**
S29	S26 OR S27 OR S28	**Search modes** - Boolean/Phrase	**View Results** (157,284)<br>**View Details**<br>**Edit**
S28	TI pediatric* OR AB pediatric* OR KW pediatric* OR TI Paediatric* OR AB paediatric* OR KW Paediatric*	**Search modes** - Boolean/Phrase	**View Results** (21,494)<br>**View Details**<br>**Edit**
S27	TI (preschool* or pre-school* or highschool* or middle school* or kindergar* or kintergar* or play school or play schools or playschool* or "after school") OR AB (preschool* or pre-school* or highschool* or middle school* or kindergar* or kintergar* or play school or play schools or playschool* or "after school") OR KW (preschool* or pre-school* or highschool* or middle school* or kindergar* or kintergar* or play school or play schools or playschool* or "after school")	**Search modes** - Boolean/Phrase	**View Results** (7,358)<br>**View Details**<br>**Edit**
S26	TI (child or childs or children or child's or childhood or teen* or adolescent* or adolescence)) OR (AB (child or childs or children or child's or childhood or teen* or	**Search modes** - Boolean/Phrase	**View Results** (149,665)<br>**View Details**<br>**Edit**

	adolescent* or adolescence)) OR KW (child or childs or children or child's or childhood or teen* or adolescent* or adolescence) OR SU (child or childs or children or child's or childhood or teen* or adolescent* or adolescence)		
S25	(TI (nutrition N2 (guide* OR guidanc* or guiding)) OR AB (nutrition N2 (guide* OR guidanc* or guiding)) OR KW (nutrition N2 (guide* OR guidanc* or guiding))) OR (TI (food N2 (choice* OR guide* OR guidanc* or guiding)) OR AB (food N2 (choice* OR guide* OR guidanc* or guiding)) OR KW (food N2 (choice* OR guide* OR guidanc* or guiding)))	**Search modes** - Boolean/Phrase	View Results (7,617)<br>View Details<br>Edit
S24	S1 OR S2 OR S3 OR S4 OR S5 OR S6 OR S7 OR S8 OR S9 OR S10 OR S11 OR S12 OR S13 OR S14 OR S15 OR S16 OR S17 OR S18 OR S19 OR S20 OR S21 OR S22 OR S23	**Search modes** - Boolean/Phrase	View Results (240,072)<br>View Details<br>Edit
S23	TI (nutrition N2 (counsel* or advice or advis*)) OR AB (nutrition N2 (counsel* or advice or advis*)) OR KW (nutrition N2 (counsel* or advice or advis*))	**Search modes** - Boolean/Phrase	View Results (917)<br>View Details<br>Edit
S22	TI (nutrition N2 (therapy or therapies)) OR AB (nutrition N2 (therapy or therapies)) OR KW (nutrition N2 (therapy or therapies))	**Search modes** - Boolean/Phrase	View Results (865)<br>View Details<br>Edit
S21	SU Food AND SU decision making	**Search modes** - Boolean/Phrase	View Results (839)<br>View Details

			Edit
S20	SU Food AND SU Choice	**Search modes** - Boolean/Phrase	View Results (235)<br>View Details<br>Edit
S19	TI (healthy meal*) or AB (healthy meal*) OR KW (healthy meal*)	**Search modes** - Boolean/Phrase	View Results (1,243)<br>View Details<br>Edit
S18	TI ((healthy or poor) N2 eating) OR AB ((healthy or poor) N2 eating) OR KW ((healthy or poor) N2 eating)	**Search modes** - Boolean/Phrase	View Results (6,023)<br>View Details<br>Edit
S17	TI (("soft drink" or "soft drinks" or ((sugar sweetened N2 (food or foods or beverage* or drink or drinks)) N2 (consum* or intake)) OR AB (("soft drink" or "soft drinks" or ((sugar sweetened N2 (food or foods or beverage* or drink or drinks)) N2 (consum* or intake)) OR KW (("soft drink" or "soft drinks" or ((sugar sweetened N2 (food or foods or beverage* or drink or drinks)) N2 (consum* or intake))	**Search modes** - Boolean/Phrase	View Results (9,273)<br>View Details<br>Edit
S16	TI ("lunch program" or "lunch programs" or "lunch programme" or "lunch programmes" or "school lunch*") OR AB ("lunch program" or "lunch programs" or "lunch programme" or "lunch programmes" or "school lunch*") OR KW ("lunch program" or "lunch programs" or "lunch programme" or "lunch programmes" or "school lunch*")	**Search modes** - Boolean/Phrase	View Results (1,520)<br>View Details<br>Edit

S15	TI (Recommended Dietary Allowance*) OR AB (Recommended Dietary Allowance*) OR KW (Recommended Dietary Allowance*	**Search modes** - Boolean/Phrase	View Results (925)<br>View Details<br>Edit
S14	TI (recommended daily allowance) OR AB (recommended daily allowance) OR KW (recommended daily allowance)	**Search modes** - Boolean/Phrase	View Results (352)<br>View Details<br>Edit
S13	TI ((nutrition or food or foods) N2 (policy or policies or program*)) OR AB ((nutrition or food or foods) N2 (policy or policies or program*)) OR KW ((nutrition or food or foods) N2 (policy or policies or program*))	**Search modes** - Boolean/Phrase	View Results (12,281)<br>View Details<br>Edit
S12	(SU Recommended Dietary Allowance*) OR (SU Nutrition Polic*) OR (SU Nutrition* Science*)	**Search modes** - Boolean/Phrase	View Results (3,617)<br>View Details<br>Edit
S11	(SU "Food Guide Pyramid") OR (SU "MyPlate") OR (SU "Nutritional Requirements") OR (SU "Dietary Reference Intakes") OR (SU "Nutritional Support Team") OR (SU "Nutrition Services")	**Search modes** - Boolean/Phrase	View Results (9,726)<br>View Details<br>Edit
S10	TI (food N2 Legislation) OR AB (food N2 Legislation) OR KW (food N2 Legislation)	**Search modes** - Boolean/Phrase	View Results (638)<br>View Details<br>Edit
S9	TI ("Supplemental Nutrition Assistance Program" or "food stamps" or "food aid" or "snap	**Search modes** - Boolean/Phrase	View Results (2,611)<br>View Details<br>Edit

	program*" or "food assistance" OR "Special Supplemental Nutrition Program for Women" or "WIC program*") OR AB ("Supplemental Nutrition Assistance Program" or "food stamps" or "food stamps" or "food aid" or "snap program*" or "food assistance" OR "Special Supplemental Nutrition Program for Women" or "WIC program*")) OR KW ("Supplemental Nutrition Assistance Program" or "food stamps" or "food stamps" or "food a ...		
S8	(SU "Food Assistance")	**Search modes** - Boolean/Phrase	**View Results** (1)<br>**View Details**<br>**Edit**
S7	TI (feeding behavior* or feeding behaviour*) or AB (feeding behavior* or feeding behaviour*) OR KW (feeding behavior* or feeding behaviour*	**Search modes** - Boolean/Phrase	**View Results** (3,087)<br>**View Details**<br>**Edit**
S6	TI ((food or eating) N2 (behavior* or behaviour* or habit or habits or preference*)) OR AB ((food or eating) N2 (behavior* or behaviour* or habit or habits or preference*)) OR KW ((food or eating) N2 (behavior* or behaviour* or habit or habits or preference*))	**Search modes** - Boolean/Phrase	**View Results** (12,794)<br>**View Details**<br>**Edit**
S5	SU Food Preference* OR SU Food Fussiness	**Search modes** - Boolean/Phrase	**View Results** (3,825)<br>**View Details**<br>**Edit**

S4	SU Food Preference* OR SU Food Fussiness	**Search modes** - Boolean/Phrase	View Results (3,825)<br>View Details<br>Edit
S3	SU Eating Behavior*	**Search modes** - Boolean/Phrase	View Results (9)<br>View Details<br>Edit
S2	TI (energy intake or (calori* N2 (intake or restriction*)) OR portion size* or serving size*) OR AB (energy intake or (calori* N2 (intake or restriction*)) OR portion size* or serving size*) OR KW (energy intake or (calori* N2 (intake or restriction*)) OR portion size* or serving size*)	**Search modes** - Boolean/Phrase	View Results (17,839)<br>View Details<br>Edit
S1	TI (diet*) or AB (diet*) OR KW diet* OR SU diet*	**Search modes** - Boolean/Phrase	View Results (199,668)<br>View Details<br>Edit

Table S5. Cochrane Database of Systematic Reviews search for Systematic Reviews Examining Impact of Pediatric Obesity Interventions on June 8, 2023

S32	S23 AND S30	**Limiters** - Year of Publication -- Reviews: 2021-2023<br>**Search modes** - Boolean/Phrase	**View Results** (16)<br>**View Details**<br>**Edit**
S31	S23 AND S30	**Search modes** - Boolean/Phrase	**View Results** (452)<br>**View Details**<br>**Edit**
S30	S24 OR S25 OR S26 OR S27 OR S28 OR S29	**Search modes** - Boolean/Phrase	**View Results** (2,680)<br>**View Details**<br>**Edit**
S29	TI paediatric* or AB paediatric*	**Search modes** - Boolean/Phrase	**View Results** (269)<br>**View Details**<br>**Edit**
S28	TI (pediatric*) OR AB pediatric*	**Search modes** - Boolean/Phrase	**View Results** (138)<br>**View Details**<br>**Edit**
S27	MW (pediatric*)	**Search modes** - Boolean/Phrase	**View Results** (18)<br>**View Details**<br>**Edit**
S26	TI (preschool* or pre-school* or highschool* or middle school* or kindergar* or kintergar* or play school or play schools or playschool* or "after school") OR AB (preschool* or pre- school* or highschool* or middle school* or kindergar* or kintergar* or play school or play schools or playschool* or "after school")	**Search modes** - Boolean/Phrase	**View Results** (57)<br>**View Details**<br>**Edit**
S25	(TI (child or childs or children or child's or childhood or	**Search modes** - Boolean/Phrase	**View Results** (2,405)<br>**View Details**

	teen* or adolescent* or adolescence)) OR (AB (child or childs or children or child's or childhood or teen* or adolescent* or adolescence))		Edit
S24	(MW "Child") OR (MW "Adolescent")	**Search modes** - Boolean/Phrase	View Results (1,501)<br>View Details<br>Edit
S23	S1 OR S2 OR S3 OR S4 OR S5 OR S6 OR S7 OR S8 OR S9 OR S10 OR S11 OR S12 OR S13 OR S14 OR S15 OR S16 OR S17 OR S18 OR S19 OR S21 OR S22	**Search modes** - Boolean/Phrase	View Results (1,265)<br>View Details<br>Edit
S22	TI (nutrition) OR AB (nutrition)	**Search modes** - Boolean/Phrase	View Results (240)<br>View Details<br>Edit
S21	TI (food or foods or eating) OR AB (food or foods or eating)	**Search modes** - Boolean/Phrase	View Results (554)<br>View Details<br>Edit
S20	TI (food N2 (guide* OR guidanc* or guiding)) OR AB (food N2 (guide* OR guidanc* or guiding))	**Search modes** - Boolean/Phrase	View Results (1)<br>View Details<br>Edit
S19	MW "Food" AND MW "Decision Making"	**Search modes** - SmartText Searching	View Results (1)<br>View Details<br>Edit
S18	MW "Food+" AND MW "Choice Behavior"	**Search modes** - SmartText Searching	View Results (1)<br>View Details<br>Edit
S17	TI (healthy meal*) or AB (healthy meal*)	**Search modes** - SmartText Searching	View Results (1)<br>View Details<br>Edit

S16	TI ((healthy or poor) N2 eating) OR AB ((healthy or poor) N2 eating)	**Search modes** - Boolean/Phrase	View Results (18)<br>View Details<br>Edit
S15	TI (("soft drink" or "soft drinks" or ((sugar sweetened N2 (food or foods or beverage* or drink or drinks)) N2 (consum* or intake)) OR AB (("soft drink" or "soft drinks" or ((sugar sweetened N2 (food or foods or beverage* or drink or drinks)) N2 (consum* or intake))	**Search modes** - Boolean/Phrase	View Results (4)<br>View Details<br>Edit
S14	TI ("lunch program" or "lunch programs" or "lunch programme" or "lunch programmes" or "school lunch*") OR AB ("lunch program" or "lunch programs" or "lunch programme" or "lunch programmes" or "school lunch*")	**Search modes** - Boolean/Phrase	View Results (1)<br>View Details<br>Edit
S13	TI (Recommended Dietary Allowance*) OR AB (Recommended Dietary Allowance*)	**Search modes** - Boolean/Phrase	View Results (1)<br>View Details<br>Edit
S12	TI (recommended daily allowance) OR AB (recommended daily allowance)	**Search modes** - Boolean/Phrase	View Results (3)<br>View Details<br>Edit
S11	(MW "Recommended Dietary Allowances") OR (MW "Nutrition Policy") OR (MW "Nutritional Sciences")	**Search modes** - Boolean/Phrase	View Results (3)<br>View Details<br>Edit

S10	MW "Nutrition Surveys" OR MW "Diet Surveys"	**Search modes** - SmartText Searching	View Results (0)<br>View Details<br>Edit
S9	TI ("Supplemental Nutrition Assistance Program" or "food stamps" or "food aid" or "snap program*" or "food assistance" OR "Special Supplemental Nutrition Program for Women" or "WIC program*") OR AB ("Supplemental Nutrition Assistance Program" or "food stamps" or "food stamps" or "food aid" or "snap program*" or "food assistance" OR "Special Supplemental Nutrition Program for Women" or "WIC program*"))	**Search modes** - Boolean/Phrase	View Results (1)<br>View Details<br>Edit
S8	(MW "Legislation, Food") OR (MW "Food Assistance")	**Search modes** - Boolean/Phrase	View Results (1)<br>View Details<br>Edit
S7	TI (feeding behavior* or feeding behaviour*) or AB (feeding behavior* or feeding behaviour*	**Search modes** - Boolean/Phrase	View Results (3)<br>View Details<br>Edit
S6	(MW "Fast Foods") OR TI ("fast food*" OR "junk food*" or "processed food*" or convenience food* or "ready to eat" or "ready prepared food*") OR AB ("fast food*" OR "junk food*" or "processed food*" or convenience food* or "ready	**Search modes** - Boolean/Phrase	View Results (6)<br>View Details<br>Edit

	to eat" or "ready prepared food*")		
S5	MW "Food Preferences" OR MW "Food Fussiness"	**Search modes** - Boolean/Phrase	View Results (1)<br>View Details<br>Edit
S4	MW "Feeding Behavior"	**Search modes** - Boolean/Phrase	View Results (8)<br>View Details<br>Edit
S3	TI (energy intake or (calori* N2 (intake or restriction*)) OR portion size* or serving size*) OR AB (energy intake or (calori* N2 (intake or restriction*)) OR portion size* or serving size*)	**Search modes** - Boolean/Phrase	View Results (51)<br>View Details<br>Edit
S2	TI (diet* or nutrition*) or AB (diet* or nutrition*)	**Search modes** - Boolean/Phrase	View Results (809)<br>View Details<br>Edit
S1	MW (Diet* OR Nutrition*)	**Search modes** - Boolean/Phrase	View Results (401)<br>View Details<br>Edit

Table S6. List of Articles During Full-Text Review of the Umbrella Review Examining Impact of Pediatric Obesity Prevention Interventions

Articles	Reason for Exclusion
1. Acosta A, Streett S, Kroh MD, et al. White Paper AGA: POWER - Practice Guide on Obesity and Weight Management, Education, and Resources. *Clin Gastroenterol Hepatol.* 2017;15(5):631-649 e610. PMID: 28242319	Population
2. Adeyemo M. Nurse Practitioners' Guide on Consumption of Hundred Percent Fruit Juice by Children. *Walden Dissertations and Doctoral Studies.* 2017.	Study Design
3. Agaronov A, Ash T, Sepulveda M, Taveras EM, Davison KK. Inclusion of Sleep Promotion in Family-Based Interventions To Prevent Childhood Obesity. *Child Obes.* 2018;14(8):485-500. PMID: 30109955	No nutrition intervention of interest
4. Agnoli C, Baroni L, Bertini I, et al. Position paper on vegetarian diets from the working group of the Italian Society of Human Nutrition. *Nutr Metab Cardiovasc Dis.* 2017;27(12):1037-1052. PMID: 29174030	Study Design
5. Akbarzadeh ZN, Mojgan; Hovsepian, Silva; Kelishadi, Roya. Dietary Patterns and Metabolic Syndrome in Children and Adolescents: A Systematic Review. *Journal of Pediatrics Review.* 2018;6(2):1-13. DOI: 10.5812/jpr.11656	Outcome
6. Akuno MH, Nocella G, Milia EP, Gutierrez L. Factors influencing the relationship between fluoride in drinking water and dental fluorosis: a ten-year systematic review and meta-analysis. *J Water Health.* 2019;17(6):845-862. PMID: 31850893	SR does not have 2 databases or ROB assessment
7. Albert Perez E, Mateu Olivares V, Martinez-Espinosa RM, Molina Vila MD, Reig Garcia-Galbis M. New Insights about How to Make an Intervention in Children and Adolescents with Metabolic Syndrome: Diet, Exercise vs. Changes in Body Composition. A Systematic Review of RCT. *Nutrients.* 2018;10(7). PMID: 29986479	SR does not have 2 databases or ROB assessment
8. Alcantara CM, Silva ANS, Pinheiro P, Queiroz MVO. Digital technologies for promotion of healthy eating habits in teenagers. *Rev Bras Enferm.* 2019;72(2):513-520. PMID: 31017217	SR does not have 2 databases or ROB assessment
9. Al-Khudairy L, Loveman E, Colquitt JL, et al. Diet, physical activity and behavioural interventions for the treatment of overweight or obese adolescents aged 12 to 17 years. *Cochrane Database Syst Rev.* 2017;6:CD012691. PMID: 28639320	Population
10. Alston L, Partridge SR. Limited dietary interventions in rural Australian communities: A systematic review. *Nutr Diet.* 2021;78(1):57-68. PMID: 32160401	Outcome

Articles	Reason for Exclusion
11. An R, Nickols-Richardson SM, Khan N, Liu J, Liu R, Clarke C. Impact of Beef and Beef Product Intake on Cognition in Children and Young Adults: A Systematic Review. *Nutrients.* 2019;11(8). PMID: 31382632	Outcome
12. Andela S, Burrows TL, Baur LA, Coyle DH, Collins CE, Gow ML. Efficacy of very low-energy diet programs for weight loss: A systematic review with meta-analysis of intervention studies in children and adolescents with obesity. *Obes Rev.* 2019;20(6):871-882. pMID: 30734459	Population
13. Angawi K, Gaissi A. Systematic Review of Setting-Based Interventions for Preventing Childhood Obesity. Biomed Res Int. 2021;2021:4477534.	SR does not have 2 databases or ROB assessment
14. Andrade J, Lotton J, Andrade J. Systematic Review: Frameworks Used in School-Based Interventions, the Impact on Hispanic Children's Obesity-Related Outcomes. *J Sch Health.* 2018;88(11):847-858. PMID: 30300932	Population
15. Appleton KM, Hemingway A, Rajska J, Hartwell H. Repeated exposure and conditioning strategies for increasing vegetable liking and intake: systematic review and meta-analyses of the published literature. *Am J Clin Nutr.* 2018;108(4):842-856. PMID: 30321277	Outcome
16. Appleton KM, Tuorila H, Bertenshaw EJ, de Graaf C, Mela DJ. Sweet taste exposure and the subsequent acceptance and preference for sweet taste in the diet: systematic review of the published literature. *Am J Clin Nutr.* 2018;107(3):405-419. PMID: 29566187	Outcome
17. Arango-Angarita A, Rodriguez-Ramirez S, Serra-Majem L, Shamah-Levy T. Dietary Energy Density and Its Association with Overweight or Obesity in Adolescents: A Systematic Review of Observational Studies. *Nutrients.* 2018;10(11). PMID: 30388849	Study Design
18. Arslanian S, Bacha F, Grey M, Marcus MD, White NH, Zeitler P. Evaluation and Management of Youth-Onset Type 2 Diabetes: A Position Statement by the American Diabetes Association. *Diabetes Care.* 2018;41(12):2648-2668. PMID: 30425094	Population
19. Ash T, Agaronov A, Young T, Aftosmes-Tobio A, Davison KK. Family-based childhood obesity prevention interventions: a systematic review and quantitative content analysis. *Int J Behav Nutr Phys Act.* 2017;14(1):113. PMID: 28836983	SR does not have 2 databases or ROB assessment
20. Auerbach BJ, Wolf FM, Hikida A, et al. Fruit Juice and Change in BMI: A Meta-analysis. *Pediatrics.* 2017;139(4). PMID: 28336576	Study Design
21. Avery A, Anderson C, McCullough F. Associations between children's diet quality and watching television during meal or snack consumption: A systematic review. *Matern Child Nutr.* 2017;13(4). PMID: 28211230	No nutrition intervention of interest

Articles	Reason for Exclusion
22. Bagherniya M, Taghipour A, Sharma M, et al. Obesity intervention programs among adolescents using social cognitive theory: a systematic literature review. *Health Educ Res.* 2018;33(1):26-39. PMID: 29293954	Population
23. Bahia L, Schaan CW, Sparrenberger K, et al. Overview of meta-analysis on prevention and treatment of childhood obesity. *J Pediatr (Rio J).* 2019;95(4):385-400. PMID: 30121174	Population
24. Bailey CJ, Drummond MJ, Ward PR. Food literacy programmes in secondary schools: a systematic literature review and narrative synthesis of quantitative and qualitative evidence. *Public Health Nutr.* 2019;22(15):2891-2913. PMID: 31290384	Outcome
25. Baker-Smith CM, de Ferranti SD, Cochran WJ, Committee On Nutrition SOGH, Nutrition. The Use of Nonnutritive Sweeteners in Children. *Pediatrics.* 2019;144(5). PMID: 31659005	Study Design
26. Baroni L, Goggi S, Battaglino R, et al. Vegan Nutrition for Mothers and Children: Practical Tools for Healthcare Providers. *Nutrients.* 2018;11(1). PMID: 30577451	Study Design
27. Bates CR, Buscemi J, Nicholson LM, Cory M, Jagpal A, Bohnert AM. Links between the organization of the family home environment and child obesity: a systematic review. *Obes Rev.* 2018;19(5):716-727. PMID: 29520946	No nutrition intervention of interest
28. Beckers D, Karssen LT, Vink JM, Burk WJ, Larsen JK. Food parenting practices and children's weight outcomes: A systematic review of prospective studies. *Appetite.* 2021;158:105010. PMID: 33075443	No nutrition intervention of interest
29. Behrman P, Demirci J, Yanez B, Beharie N, Laroche H. Society of Behavioral Medicine (SBM) position statement: SBM urges Congress to preserve and increase the financing of federally funded nutrition assistance programs and services. *Transl Behav Med.* 2019;9(1):170-174. PMID: 29726980	Study Design
30. Benjamin-Neelon SE. Position of the Academy of Nutrition and Dietetics: Benchmarks for Nutrition in Child Care. *J Acad Nutr Diet.* 2018;118(7):1291-1300. PMID: 29937055	Study Design
31. Black AP, D'Onise K, McDermott R, Vally H, O'Dea K. How effective are family-based and institutional nutrition interventions in improving children's diet and health? A systematic review. *BMC Public Health.* 2017;17(1):818. PMID: 29041899	Outcome
32. Blaine RE, Kachurak A, Davison KK, Klabunde R, Fisher JO. Food parenting and child snacking: a systematic review. *Int J Behav Nutr Phys Act.* 2017;14(1):146. PMID: 29096640	Outcome

Articles	Reason for Exclusion
33. Bleich SN, Vercammen KA. The negative impact of sugar-sweetened beverages on children's health: an update of the literature. *BMC Obes.* 2018;5:6. PMID: 29484192	SR does not have 2 databases or ROB assessment
34. Bleich SN, Vercammen KA, Zatz LY, Frelier JM, Ebbeling CB, Peeters A. Interventions to prevent global childhood overweight and obesity: a systematic review. Lancet Diabetes Endocrinol. 2018;6(4):332-346.	No meta-analysis or graded certainty of evidence
35. Boff RM, Liboni RPA, Batista IPA, de Souza LH, Oliveira MDS. Weight loss interventions for overweight and obese adolescents: a systematic review. *Eat Weight Disord.* 2017;22(2):211-229. PMID: 27542161	Population
36. Bohnert A, Zarrett N, Beets MW, et al. Society of Behavioral Medicine (SBM) position statement: SBM supports curbing summertime weight gain among America's youth. *Transl Behav Med.* 2017;7(4):912-914. PMID: 28660591	No nutrition intervention of interest
37. Bonvicini L, Pingani I, Venturelli F, et al. Effectiveness of mobile health interventions targeting parents to prevent and treat childhood Obesity: Systematic review. Prev Med Rep. 2022;29:101940.	Population
38. Boyland E, McGale L, Maden M, Hounsome J, Boland A, Jones A. Systematic review of the effect of policies to restrict the marketing of foods and non-alcoholic beverages to which children are exposed. Obes Rev. 2022;23(8):e13447.	Other
39. Bray GA, Heisel WE, Afshin A, et al. The Science of Obesity Management: An Endocrine Society Scientific Statement. *Endocr Rev.* 2018;39(2):79-132. PMID: 29518206	Population
40. Brink E, van Rossum C, Postma-Smeets A, et al. Development of healthy and sustainable food-based dietary guidelines for the Netherlands. *Public Health Nutr.* 2019;22(13):2419-2435. PMID: 31262374	Study Design
41. Brown T, O'Malley C, Blackshaw J, et al. Exploring the evidence base for Tier 3 specialist weight management interventions for children aged 2-18 years in the UK: a rapid systematic review. *J Public Health (Oxf).* 2018;40(4):835-847. PMID: 29228233	Population
42. Browne J, Adams K, Atkinson P, Gleeson D, Hayes R. Food and nutrition programs for Aboriginal and Torres Strait Islander Australians: an overview of systematic reviews. *Aust Health Rev.* 2018;42(6):689-697. PMID: 28923162	Population
43. Bucher T, Murawski B, Duncanson K, Labbe D, Van der Horst K. The effect of the labelled serving size on consumption: A systematic review. *Appetite.* 2018;128:50-57. PMID: 29859775	Population

Articles	Reason for Exclusion
44. Buckland NJ, Er V, Redpath I, Beaulieu K. Priming food intake with weight control cues: systematic review with a meta-analysis. *Int J Behav Nutr Phys Act.* 2018;15(1):66. PMID: 29986712	Population
45. Burnett AJ, Lamb KE, McCann J, Worsley A, Lacy KE. Parenting styles and the dietary intake of pre-school children: a systematic review. *Psychol Health.* 2020;35(11):1326-1345. PMID: 32255381	No nutrition intervention of interest
46. Burrows T, Goldman S, Pursey K, Lim R. Is there an association between dietary intake and academic achievement: a systematic review. *J Hum Nutr Diet.* 2017;30(2):117-140. PMID: 27599886	No nutrition intervention of interest
47. Buscemi J, Odoms-Young A, Yaroch AL, et al. Society of Behavioral Medicine position statement: retain school meal standards and healthy school lunches. *Transl Behav Med.* 2019;9(2):389-390. PMID: 29669135	Study Design
48. Cafe ACC, Lopes CAO, Novais RLR, et al. Intake of Sugar-Sweetened Beverages, Milk and Its Association with Body Mass Index in Adolescence: A Systematic Review. *Rev Paul Pediatr.* 2018;36(1):91-99. PMID: 29513855	Study Design
49. Calvert S, Dempsey RC, Povey R. Delivering in-school interventions to improve dietary behaviours amongst 11- to 16-year-olds: A systematic review. *Obes Rev.* 2019;20(4):543-553. PMID: 30550629	Outcome
50. Campbell ET, Franks AT, Joseph PV. Adolescent obesity in the past decade: A systematic review of genetics and determinants of food choice. *J Am Assoc Nurse Pract.* 2019;31(6):344-351. PMID: 31157651	No nutrition intervention of interest
51. Cashman KD, Ritz C. Individual participant data (IPD)-level meta-analysis of randomised controlled trials among dark-skinned populations to estimate the dietary requirement for vitamin D. *Syst Rev.* 2019;8(1):128. PMID: 31138301	Population
52. Cavero-Redondo I, Alvarez-Bueno C, Sotos-Prieto M, Gil A, Martinez-Vizcaino V, Ruiz JR. Milk and Dairy Product Consumption and Risk of Mortality: An Overview of Systematic Reviews and Meta-Analyses. *Adv Nutr.* 2019;10(suppl_2):S97-S104. PMID: 31089743	Population
53. Centeno Tablante E, Pachon H, Guetterman HM, Finkelstein JL. Fortification of wheat and maize flour with folic acid for population health outcomes. *Cochrane Database Syst Rev.* 2019;7:CD012150. PMID: 31257574	Population
54. Chai LK, Collins C, May C, Brain K, Wong See D, Burrows T. Effectiveness of family-based weight management interventions for children with overweight and obesity: an umbrella review. *JBI Database System Rev Implement Rep.* 2019;17(7):1341-1427. PMID: 31021970	Population

Articles	Reason for Exclusion
55. Chai LK, Farletti R, Fathi L, Littlewood R. A Rapid Review of the Impact of Family-Based Digital Interventions for Obesity Prevention and Treatment on Obesity-Related Outcomes in Primary School-Aged Children. Nutrients. 2022;14(22).	Population
56. Chamberlain JJ, Doyle-Delgado K, Peterson L, Skolnik N. Diabetes Technology: Review of the 2019 American Diabetes Association Standards of Medical Care in Diabetes. *Ann Intern Med.* 2019;171(6):415-420. PMID: 31404925	Study Design
57. Chambers T, Segal A, Sassi F. Interventions using behavioural insights to influence children's diet-related outcomes: A systematic review. *Obes Rev.* 2021;22(2):e13152. PMID: 33462932	Outcome
58. Chan AS, Tran TTK, Hsu YH, Liu SYS, Kroon J. A systematic review of dietary acids and habits on dental erosion in adolescents. *Int J Paediatr Dent.* 2020;30(6):713-733. PMID: 32246790	Outcome
59. Chan CY, Mohamed N, Ima-Nirwana S, Chin KY. A Review of Knowledge, Belief and Practice Regarding Osteoporosis among Adolescents and Young Adults. *Int J Environ Res Public Health.* 2018;15(8). PMID: 30103534	SR does not have 2 databases or ROB assessment
60. Chan CL, Tan PY, Gong YY. Evaluating the impacts of school garden-based programmes on diet and nutrition-related knowledge, attitudes and practices among the school children: a systematic review. BMC Public Health. 2022;22(1):1251.	Outcome
61. Chau MM, Burgermaster M, Mamykina L. The use of social media in nutrition interventions for adolescents and young adults-A systematic review. *Int J Med Inform.* 2018;120:77-91. PMID: 30409348	Population
62. Chaudhary A, Sudzina F, Mikkelsen BE. Promoting Healthy Eating among Young People-A Review of the Evidence of the Impact of School-Based Interventions. *Nutrients.* 2020;12(9). PMID: 32971883	No meta-analysis or graded certainty of evidence
63. Chen LH, Wang YF, Xu QH, Chen SS. Omega-3 fatty acids as a treatment for non-alcoholic fatty liver disease in children: A systematic review and meta-analysis of randomized controlled trials. *Clin Nutr.* 2018;37(2):516-521. PMID: 28040302	Population
64. Chitturi S, Wong VW, Chan WK, et al. The Asia-Pacific Working Party on Non-alcoholic Fatty Liver Disease guidelines 2017-Part 2: Management and special groups. *J Gastroenterol Hepatol.* 2018;33(1):86-98. PMID: 28692197	Study Design

Articles	Reason for Exclusion
65. Chu A, Holdaway C, Varma T, Petocz P, Samman S. Lower Serum Zinc Concentration Despite Higher Dietary Zinc Intake in Athletes: A Systematic Review and Meta-analysis. *Sports Med.* 2018;48(2):327-336. PMID: 39164533	Population
66. Chung SJ, Ersig AL, McCarthy AM. The Influence of Peers on Diet and Exercise Among Adolescents: A Systematic Review. *J Pediatr Nurs.* 2017;36:44-56. PMID: 28888511	No nutrition intervention of interest
67. Cohen JFW, Hecht AA, McLoughlin GM, Turner L, Schwartz MB. Universal School Meals and Associations with Student Participation, Attendance, Academic Performance, Diet Quality, Food Security, and Body Mass Index: A Systematic Review. Nutrients. 2021;13(3).	No meta-analysis or graded certainty of evidence
68. Cole NC, An R, Lee SY, Donovan SM. Correlates of picky eating and food neophobia in young children: a systematic review and meta-analysis. *Nutr Rev.* 2017;75(7):516-532. PMID: 28535257	Population
69. Collado-Soler R, Alférez-Pastor M, Torres FL, Trigueros R, Aguilar-Parra JM, Navarro N. A Systematic Review of Healthy Nutrition Intervention Programs in Kindergarten and Primary Education. Nutrients. 2023;15(3).	SR does not have 2 databases or ROB assessment
70. Collese TS, Nascimento-Ferreira MV, de Moraes ACF, et al. Role of fruits and vegetables in adolescent cardiovascular health: a systematic review. *Nutr Rev.* 2017;75(5):339-349. PMID: 28475799	No nutrition intervention of interest
71. Colley P, Myer B, Seabrook J, Gilliland J. The Impact of Canadian School Food Programs on Children's Nutrition and Health: A Systematic Review. *Can J Diet Pract Res.* 2019;80(2):79-86. PMID: 30430855	Outcome
72. Corepal R, Tully MA, Kee F, Miller SJ, Hunter RF. Behavioural incentive interventions for health behaviour change in young people (5-18years old): A systematic review and meta-analysis. *Prev Med.* 2018;110:55-66. PMID: 29432789	No nutrition intervention of interest
73. Costa CS, Del-Ponte B, Assuncao MCF, Santos IS. Consumption of ultra-processed foods and body fat during childhood and adolescence: a systematic review. *Public Health Nutr.* 2018;21(1):148-159. PMID: 28676132	Study Design
74. Coughlin SS, Smith SA. Community-Based Participatory Research to Promote Healthy Diet and Nutrition and Prevent and Control Obesity Among African-Americans: a Literature Review. *J Racial Ethn Health Disparities.* 2017;4(2):259-268. PMID: 27059053	SR does not have 2 databases or ROB assessment
75. Craig JV, Bunn DK, Hayhoe RP, Appleyard WO, Lenaghan EA, Welch AA. Relationship between the Mediterranean dietary pattern and musculoskeletal health in children, adolescents, and adults: systematic review and evidence map. *Nutr Rev.* 2017;75(10):830-857. PMID: 29028268	Population

Articles	Reason for Exclusion
76. Cunha CM, Costa PRF, de Oliveira LPM, Queiroz VAO, Pitangueira JCD, Oliveira AM. Dietary patterns and cardiometabolic risk factors among adolescents: systematic review and meta-analysis. *Br J Nutr.* 2018;119(8):859-879. PMID: 29644953	No nutrition intervention of interest
77. da Costa Peres CM, Gardone DS, Costa BVL, Duarte CK, Pessoa MC, Mendes LL. Retail food environment around schools and overweight: a systematic review. *Nutr Rev.* 2020;78(10):841-856. PMID: 31968100	No meta-analysis or graded certainty of evidence
78. Dallacker M, Hertwig R, Mata J. Quality matters: A meta-analysis on components of healthy family meals. *Health Psychol.* 2019;38(12):1137-1149. PMID: 31556657	No nutrition intervention of interest
79. Darling AL, Manders RJF, Sahni S, et al. Dietary protein and bone health across the life-course: an updated systematic review and meta-analysis over 40 years. *Osteoporos Int.* 2019;30(4):741-761. PMID: 30903209	SR does not have 2 databases or ROB assessment
80. Darling KE, Sato AF. Systematic Review and Meta-Analysis Examining the Effectiveness of Mobile Health Technologies in Using Self-Monitoring for Pediatric Weight Management. *Child Obes.* 2017;13(5):347-355. PMID: 28471699	Population
81. Das JK, Salam RA, Mahmood SB, et al. Food fortification with multiple micronutrients: impact on health outcomes in general population. *Cochrane Database Syst Rev.* 2019;12:CD011400. PMID: 31849042	No nutrition intervention of interest
82. Dawodu A, Cleaver K. Behavioural correlates of energy drink consumption among adolescents: A review of the literature. *J Child Health Care.* 2017;21(4):446-462. PMID: 29110525	No nutrition intervention of interest
83. de Carvalho GB, Dias-Vasconcelos NL, Santos RKF, Brandao-Lima PN, da Silva DG, Pires LV. Effect of different dietary patterns on glycemic control in individuals with type 2 diabetes mellitus: A systematic review. *Crit Rev Food Sci Nutr.* 2020;60(12):1999-2010. PMID: 31204492	Population
84. de Droog SM, van Nee R, Govers M, Buijzen M. Promoting toddlers' vegetable consumption through interactive reading and puppetry. *Appetite.* 2017;116:75-81. PMID: 28438548	Study Design
85. de Lamas C, de Castro MJ, Gil-Campos M, Gil A, Couce ML, Leis R. Effects of Dairy Product Consumption on Height and Bone Mineral Content in Children: A Systematic Review of Controlled Trials. *Adv Nutr.* 2019;10(suppl_2):S88-S96. PMID: 31089738	No nutrition intervention of interest
86. de Oliveira JF, Romano MCC, Araújo A, Fiedler MW. Effect of Educational Software on Adolescents. *J Nurs. UFPE* 2018;12(11):3078-3088. DOI: 10.5205/1981-8963-v12i11a237657p3078-3088-2018	SR does not have 2 databases or ROB assessment
87. DeCosta P, Moller P, Frost MB, Olsen A. Changing children's eating behaviour - A review of experimental research. *Appetite.* 2017;113:327-357. PMID: 28286164	SR does not have 2 databases or ROB assessment

Articles	Reason for Exclusion
88. Deeb A, Elbarbary N, Smart CE, et al. ISPAD Clinical Practice Consensus Guidelines: Fasting during Ramadan by young people with diabetes. *Pediatric Diabetes.* 2019;21(1):5-17. DOI: 10.1111/pedi.12920	Study Design
89. Della Corte KW, Perrar I, Penczynski KJ, Schwingshackl L, Herder C, Buyken AE. Effect of Dietary Sugar Intake on Biomarkers of Subclinical Inflammation: A Systematic Review and Meta-Analysis of Intervention Studies. *Nutrients.* 2018;10(5). PMID: 29757229	Population
90. Denova-Gutierrez E, Mendez-Sanchez L, Munoz-Aguirre P, Tucker KL, Clark P. Dietary Patterns, Bone Mineral Density, and Risk of Fractures: A Systematic Review and Meta-Analysis. *Nutrients.* 2018;10(12). PMID: 30563066	Population
91. De-Regil LM, Jefferds MED, Pena-Rosas JP. Point-of-use fortification of foods with micronutrient powders containing iron in children of preschool and school-age. *Cochrane Database Syst Rev.* 2017;11:CD009666. PMID: 29168569	Other
92. Deren K, Weghuber D, Caroli M, et al. Consumption of Sugar-Sweetened Beverages in Paediatric Age: A Position Paper of the European Academy of Paediatrics and the European Childhood Obesity Group. *Ann Nutr Metab.* 2019;74(4):296-302. PMID: 31013493	Study Design
93. Dias JD, Domingues AN, Tibes CM, Zem-Mascarenhas SH, Fonseca LMM. Serious games as an educational strategy to control childhood obesity: a systematic literature review1. *Rev Lat Am Enfermagem.* 2018;26:e3036. PMID: 30183872	SR does not have 2 databases or ROB assessment
94. Divaris K, Bhaskar V, McGraw KA. Pediatric obesity-related curricular content and training in dental schools and dental hygiene programs: systematic review and recommendations. *J Public Health Dent.* 2017;77 Suppl 1:S96-S103. PMID: 28708273	SR does not have 2 databases or ROB assessment
95. do Amaral EMGR, de Carvalho Silva Vargas F, Dos Santos Chagas CM, Toral N. Nutritional interventions for adolescents using information and communication technologies (ICTs): A systematic review. *PLoS One.* 2017;12(9):e0184509. PMID: 28961246	Outcome
96. do Amaral EMGR, Silva PO, Nakabayashi J, Bandeira MV, Toral N, Monteiro R. Family meal frequency and its association with food consumption and nutritional status in adolescents: A systematic review. *PLoS One.* 2020;15(9):e0239274. PMID: 32946506	No meta-analysis or graded certainty of evidence
97. Driggers J. 2017 Hypertension Guidelines for Children. *SOY CONNECT.* 2018;26(3):5-6.	Study Design

Articles	Reason for Exclusion
98. Durbin J. Pediatric Obesity in Primary Practice: A Review of the Literature. *PEDIATR NURS.* 2018;44(4):202-206. DOI: 10.1249/ MSS. 00000 0 0000000649	SR does not have 2 databases or ROB assessment
99. Eaton JC, Rothpletz-Puglia P, Dreker MR, et al. Effectiveness of provision of animal-source foods for supporting optimal growth and development in children 6 to 59 months of age. *Cochrane Database Syst Rev.* 2019;2:CD012818. PMID: 30779870	Population
100. Edefonti V, Bravi F, Ferraroni M. Breakfast and behavior in morning tasks: Facts or fads? *J Affect Disord.* 2017;224:16-26. PMID: 28062077	SR does not have 2 databases or ROB assessment
101. Egbert AH, Creber C, Loren DM, Bohnert AM. Executive function and dietary intake in youth: A systematic review of the literature. *Appetite.* 2019;139:197-212. PMID: 31014952	SR does not have 2 databases or ROB assessment
102. Eichler K, Hess S, Twerenbold C, Sabatier M, Meier F, Wieser S. Health effects of micronutrient fortified dairy products and cereal food for children and adolescents: A systematic review. *PLoS One.* 2019;14(1):e0210899. PMID: 30673769	No nutrition intervention of interest
103. Eid NMS. Child Nutrition Programs in Kindergarten Schools Implemented by the Governmental Sector and Global Nutrition Consulting Companies: A Systematic Review. *Current Research in Nutrition & Food Science.* 2018;6(3):656-663. DOI: 10.12944/CRNFSJ.6.3.07	SR does not have 2 databases or ROB assessment
104. Ells LJ, Rees K, Brown T, et al. Interventions for treating children and adolescents with overweight and obesity: an overview of Cochrane reviews. *Int J Obes (Lond).* 2018;42(11):1823-1833. PMID: 30301964	SR does not have 2 databases or ROB assessment
105. Eva JJ, Kassab YW, Neoh CF, et al. Self-Care and Self-Management Among Adolescent T2DM Patients: A Review. *Front Endocrinol (Lausanne).* 2018;9:489. PMID: 30459707	SR does not have 2 databases or ROB assessment
106. Evans RA, Frese M, Romero J, Cunningham JH, Mills KE. Chronic fructose substitution for glucose or sucrose in food or beverages has little effect on fasting blood glucose, insulin, or triglycerides: a systematic review and meta-analysis. *Am J Clin Nutr.* 2017;106(2):519-529. PMID: 28592603	Population
107. Evans RA, Frese M, Romero J, Cunningham JH, Mills KE. Fructose replacement of glucose or sucrose in food or beverages lowers postprandial glucose and insulin without raising triglycerides: a systematic review and meta-analysis. *Am J Clin Nutr.* 2017;106(2):506-518. PMID: 28592611	Population

Articles	Reason for Exclusion
108. Fair KN, Solari Williams KD, Warren J, McKyer ELJ, Ory MG. The Influence of Organizational Culture on School-Based Obesity Prevention Interventions: A Systematic Review of the Literature. *J Sch Health.* 2018;88(6):462-473. PMID: 29748998	Study Design
109. Fardet A, Richonnet C, Mazur A. Association between consumption of fruit or processed fruit and chronic diseases and their risk factors: a systematic review of meta-analyses. *Nutr Rev.* 2019;77(6):376-387. PMID: 30995309	Study Design
110. Fattore E, Botta F, Agostoni C, Bosetti C. Effects of free sugars on blood pressure and lipids: a systematic review and meta-analysis of nutritional isoenergetic intervention trials. *Am J Clin Nutr.* 2017;105(1):42-56. PMID: 28003201	Population
111. Fidler Mis N, Braegger C, Bronsky J, et al. Sugar in Infants, Children and Adolescents: A Position Paper of the European Society for Paediatric Gastroenterology, Hepatology and Nutrition Committee on Nutrition. *J Pediatr Gastroenterol Nutr.* 2017;65(6):681-696. PMID: 28922262	Study Design
112. Flodgren G, Goncalves-Bradley DC, Summerbell CD. Interventions to change the behaviour of health professionals and the organisation of care to promote weight reduction in children and adults with overweight or obesity. *Cochrane Database Syst Rev.* 2017;11:CD000984. PMID: 29190418	Population
113. Fornari E, Brusati M, Maffeis C. Nutritional Strategies for Childhood Obesity Prevention. Life (Basel). 2021;11(6).	Study Design
114. Flynn JT, Kaelber DC, Baker-Smith CM, et al. Clinical Practice Guideline for Screening and Management of High Blood Pressure in Children and Adolescents. *Pediatrics.* 2017;140(3). PMID: 28827377	No nutrition intervention of interest
115. Folkvord F, van 't Riet J. The persuasive effect of advergames promoting unhealthy foods among children: A meta-analysis. *Appetite.* 2018;129:245-251. PMID: 30031786	Outcome
116. Francis L, Shodeinde L, Black MM, Allen J. Examining the Obesogenic Attributes of the Family Child Care Home Environment: A Literature Review. *J Obes.* 2018;2018:3490651. PMID: 29983998	SR does not have 2 databases or ROB assessment
117. Franse CB, Boelens M, Fries LR, Constant F, van Grieken A, Raat H. Interventions to increase the consumption of water among children: A systematic review and meta-analysis. *Obes Rev.* 2020;21(7):e13015. PMID: 32167233	Outcome
118. Gaidhane AM, Sinha A, Khatib MN, et al. A Systematic Review on Effect of Electronic Media on Diet, Exercise, and Sexual Activity among Adolescents. Indian J Community Med. 2018;43(Suppl 1):S56-s65.	Population

Articles	Reason for Exclusion
119. Gao X, Zhang H, Guo XF, Li K, Li S, Li D. Effect of Betaine on Reducing Body Fat-A Systematic Review and Meta-Analysis of Randomized Controlled Trials. *Nutrients.* 2019;11(10). PMID: 31623137	Population
120. Garbers S, Hunersen K, Nechitilo M, et al. Healthy Weight and Cardiovascular Health Promotion Interventions for Adolescent and Young Adult Males of Color: A Systematic Review. *Am J Mens Health.* 2018;12(5):1328-1351. PMID: 29808765	SR does not have 2 databases or ROB assessment
121. Garcia RI, Kleinman D, Holt K, et al. Healthy Futures: Engaging the oral health community in childhood obesity prevention - Conference summary and recommendations. *J Public Health Dent.* 2017;77 Suppl 1:S136-S140. PMID: 28621818	Study Design
122. Garrido-Miguel M, Oliveira A, Cavero-Redondo I, et al. Prevalence of Overweight and Obesity among European Preschool Children: A Systematic Review and Meta-Regression by Food Group Consumption. *Nutrients.* 2019;11(7). PMID: 31340602	Study Design
123. Ghobadi S, Hassanzadeh-Rostami Z, Salehi-Marzijarani M, et al. Association of eating while television viewing and overweight/obesity among children and adolescents: a systematic review and meta-analysis of observational studies. *Obes Rev.* 2018;19(3):313-320. PMID: 29266643	No nutrition intervention of interest
124. Gibson PS, Lang S, Dhawan A, et al. Systematic Review: Nutrition and Physical Activity in the Management of Paediatric Nonalcoholic Fatty Liver Disease. *J Pediatr Gastroenterol Nutr.* 2017;65(2):141-149. PMID: 28737568	Population
125. Godin KM, Kirkpatrick SI, Hanning RM, Stapleton J, Leatherdale ST. Examining Guidelines for School-Based Breakfast Programs in Canada: A Systematic Review of the Grey Literature. *Can J Diet Pract Res.* 2017;78(2):92-100. PMID: 28145767	SR does not have 2 databases or ROB assessment
126. Goldthorpe J, Epton T, Keyworth C, Calam R, Armitage CJ. Are primary/elementary school-based interventions effective in preventing/ameliorating excess weight gain? A systematic review of systematic reviews. *Obes Rev.* 2020;21(6):e13001. PMID: 32162477	Study Design
127. Gonzalez KE, Freysteinson W. Youth-Onset Type 2 Diabetes Mellitus In Primary Practice: A Review. *Pediatric Nursing.* 2019;45(6):293-297.	SR does not have 2 databases or ROB assessment
128. Goodyear VA, Wood G, Skinner B, Thompson JL. The effect of social media interventions on physical activity and dietary behaviours in young people and adults: a systematic review. Int J Behav Nutr Phys Act. 2021;18(1):72.	Population

Articles	Reason for Exclusion
129. Goran MI, Riemer SL, Alderete TL. Simplified and age-appropriate recommendations for added sugars in children. *Pediatr Obes.* 2018;13(4):269-272. PMID: 28921869	Study Design
130. Gordon K, Dynan L, Siegel R. Healthier Choices in School Cafeterias: A Systematic Review of Cafeteria Interventions. *J Pediatr.* 2018;203:273-279 e272. PMID: 30213461	SR does not have 2 databases or ROB assessment
131. Gori D, Guaraldi F, Cinocca S, Moser G, Rucci P, Fantini MP. Effectiveness of educational and lifestyle interventions to prevent paediatric obesity: systematic review and meta-analyses of randomized and non-randomized controlled trials. *Obes Sci Pract.* 2017;3(3):235-248. PMID: 29071100	Population
132. Gow ML, Tee MSY, Garnett SP, et al. Pediatric obesity treatment, self-esteem, and body image: A systematic review with meta-analysis. *Pediatr Obes.* 2020;15(3):e12600. PMID: 32020780	Population
133. Graziose MM, Downs SM, O'Brien Q, Fanzo J. Systematic review of the design, implementation and effectiveness of mass media and nutrition education interventions for infant and young child feeding. *Public Health Nutr.* 2018;21(2):273-287. PMID: 29081315	Population
134. Grimes CA, Bolton KA, Booth AB, et al. The association between dietary sodium intake, adiposity and sugar-sweetened beverages in children and adults: a systematic review and meta-analysis. *Br J Nutr.* 2021;126(3):409-427. PMID: 33054868	Study Design
135. Grossman Z, Hadjipanayis A, Stiris T, et al. Vitamin D in European children-statement from the European Academy of Paediatrics (EAP). *Eur J Pediatr.* 2017;176(6):829-831. PMID: 28401345	Other
136. Guzek D, Glabska D, Groele B, Gutkowska K. Role of fruit and vegetables for the mental health of children: a systematic review. *Rocz Panstw Zakl Hig.* 2020;71(1):5-13. PMID: 32227779	SR does not have 2 databases or ROB assessment
137. Haines J, Haycraft E, Lytle L, et al. Nurturing Children's Healthy Eating: Position statement. *Appetite.* 2019;137:124-133. PMID: 30797837	Study Design
138. Hamid MA, Sazlina SG. Interventions for obesity among schoolchildren: A systematic review and meta-analyses. PLoS One. 2019;14(1):e0209746. PMID: 30625165	Population

Articles	Reason for Exclusion
139. Hammersley M, Jones R, Okely A. Parent-focused childhood overweight and obesity eHealth interventions: A systematic review and meta-analysis. *Obesity research & clinical practice.* 2019;13(1):84.	No nutrition intervention of interest
140. Harrison SE, Greenhouse D. Dietary and Nutrition Recommendations in Pediatric Primary Care: A Call to Action. *South Med J.* 2018;111(1):12-17. PMID: 29298363	Study Design
141. Hasan B, Thompson WG, Almasri J, et al. The effect of culinary interventions (cooking classes) on dietary intake and behavioral change: a systematic review and evidence map. *BMC Nutr.* 2019;5:29. PMID: 32153942	Population
142. Hauger H, Laursen RP, Ritz C, Molgaard C, Lind MV, Damsgaard CT. Effects of vitamin D supplementation on cardiometabolic outcomes in children and adolescents: a systematic review and meta-analysis of randomized controlled trials. *Eur J Nutr.* 2020;59(3):873-884. PMID: 32060613	Outcome
143. Hayba N, Rissel C, Allman Farinelli M. Effectiveness of lifestyle interventions in preventing harmful weight gain among adolescents: A systematic review of systematic reviews. Obes Rev. 2021;22(2):e13109.	Study Design
144. Hayes D, Contento IR, Weekly C. Position of the Academy of Nutrition and Dietetics, Society for Nutrition Education and Behavior, and School Nutrition Association: Comprehensive Nutrition Programs and Services in Schools. *J Acad Nutr Diet.* 2018;118(5):913-919. PMID: 29703342	Study Design
145. He S, Stein AD. Early-Life Nutrition Interventions and Associated Long-Term Cardiometabolic Outcomes: A Systematic Review and Meta-Analysis of Randomized Controlled Trials. Adv Nutr. 2021;12(2):461-489.	No nutrition intervention of interest
146. Heerman WJ, JaKa MM, Berge JM, et al. The dose of behavioral interventions to prevent and treat childhood obesity: a systematic review and meta-regression. *Int J Behav Nutr Phys Act.* 2017;14(1):157. PMID: 29141651	No nutrition intervention of interest
147. Hendrie GA, Lease HJ, Bowen J, Baird DL, Cox DN. Strategies to increase children's vegetable intake in home and community settings: a systematic review of literature. *Matern Child Nutr.* 2017;13(1). PMID: 26924706	Outcome
148. Henry BW, Ziegler J, Parrott JS, Handu D. Pediatric Weight Management Evidence-Based Practice Guidelines: Components and Contexts of Interventions. *J Acad Nutr Diet.* 2018;118(7):1301-1311 e1323. PMID: 29233517	Population

Articles	Reason for Exclusion
149. Hens W, Vissers D, Hansen D, et al. The effect of diet or exercise on ectopic adiposity in children and adolescents with obesity: a systematic review and meta-analysis. *Obes Rev.* 2017;18(11):1310-1322. PMID: 28913977	Population
150. Heshmati J, Sepidarkish M, Namazi N, et al. Impact of Dietary Calcium Supplement on Circulating Lipoprotein Concentrations and Atherogenic Indices in Overweight and Obese Individuals: A Systematic Review. *J Diet Suppl.* 2019;16(3):357-367. PMID:29561197	Population
151. Heyman MB, Abrams SA, Section On Gastroenterology H, Nutrition, Committee On N. Fruit Juice in Infants, Children, and Adolescents: Current Recommendations. *Pediatrics.* 2017;139(6). PMID: 28562300	Study Design
152. Hilger-Kolb J, Bosle C, Motoc I, Hoffmann K. Associations between dietary factors and obesity-related biomarkers in healthy children and adolescents - a systematic review. *Nutr J.* 2017;16(1):85. PMID: 29282082	Study Design
153. Hillier-Brown FC, Summerbell CD, Moore HJ, et al. The impact of interventions to promote healthier ready-to-eat meals (to eat in, to take away or to be delivered) sold by specific food outlets open to the general public: a systematic review. *Obes Rev.* 2017;18(2):227-246. PMID: 27899007	Population
154. Ho RS, Chan EK, Liu KK, Wong SH. Active video game on children and adolescents' physical activity and weight management: A network meta-analysis. Scand J Med Sci Sports. 2022;32(8):1268-1286.	No nutrition intervention of interest
155. Ho HVT, Jovanovski E, Zurbau A, et al. A systematic review and meta-analysis of randomized controlled trials of the effect of konjac glucomannan, a viscous soluble fiber, on LDL cholesterol and the new lipid targets non-HDL cholesterol and apolipoprotein B. *Am J Clin Nutr.* 2017;105(5):1239-1247. PMID: 28356275	Outcome
156. Hoare E, Varsamis P, Owen N, Dunstan DW, Jennings GL, Kingwell BA. Sugar- and Intense-Sweetened Drinks in Australia: A Systematic Review on Cardiometabolic Risk. *Nutrients.* 2017;9(10). PMID: 28956823	SR does not have 2 databases or ROB assessment
157. Hodder RK, O'Brien KM, Stacey FG, et al. Interventions for increasing fruit and vegetable consumption in children aged five years and under. *Cochrane Database Syst Rev.* 2019;2019(11). PMID: 31697869	Population
158. Hojsak I, Bronsky J, Campoy C, et al. Young Child Formula: A Position Paper by the ESPGHAN Committee on Nutrition. *J Pediatr Gastroenterol Nutr.* 2018;66(1):177-185. PMID: 29095351	Population

Articles	Reason for Exclusion
159. Holley CE, Mason C. A Systematic Review of the Evaluation of Interventions to Tackle Children's Food Insecurity. *Curr Nutr Rep.* 2019;8(1):11-27. PMID: 30762204	Outcome
160. Holmes WS, Moorhead SA, Coates VE, Bond RR, Zheng H. Impact of digital technologies for communicating messages on weight loss maintenance: a systematic literature review. *Eur J Public Health.* 2019;29(2):320-328. PMID: 30239699	SR does not have 2 databases or ROB assessment
161. Hombali AS, Solon JA, Venkatesh BT, Nair NS, Pena-Rosas JP. Fortification of staple foods with vitamin A for vitamin A deficiency. *Cochrane Database Syst Rev.* 2019;5:CD010068. PMID: 31074495	Other
162. Hsiao BS, Sibeko L, Troy LM. A Systematic Review of Mobile Produce Markets: Facilitators and Barriers to Use, and Associations with Reported Fruit and Vegetable Intake. *J Acad Nutr Diet.* 2019;119(1):76-97 e71. PMID: 29764767	Population
163. Hsu MSH, Rouf A, Allman-Farinelli M. Effectiveness and Behavioral Mechanisms of Social Media Interventions for Positive Nutrition Behaviors in Adolescents: A Systematic Review. *J Adolesc Health.* 2018;63(5):531-545. PMID: 30197198	Outcome
164. Iaccarino Idelson P, Scalfi L, Valerio G. Adherence to the Mediterranean Diet in children and adolescents: A systematic review. *Nutr Metab Cardiovasc Dis.* 2017;27(4):283-299. PMID: 28254269	Study Design
165. Innella N, Jameson BE. Interventions that impact weight status in Hispanic preschool children. *Public Health Nurs.* 2020;37(1):25-38. PMID: 31633235	SR does not have 2 databases or ROB assessment
166. Itkonen ST, Erkkola M, Lamberg-Allardt CJE. Vitamin D Fortification of Fluid Milk Products and Their Contribution to Vitamin D Intake and Vitamin D Status in Observational Studies-A Review. *Nutrients.* 2018;10(8). PMID: 30096919	SR does not have 2 databases or ROB assessment
167. Jacob CM, Hardy-Johnson PL, Inskip HM, et al. A systematic review and meta-analysis of school-based interventions with health education to reduce body mass index in adolescents aged 10 to 19 years. *Int J Behav Nutr Phys Act.* 2021;18(1):1. PMID: 33397403	No nutrition intervention of interest
168. Jacob CM, Newell ML, Hanson M. Narrative review of reviews of preconception interventions to prevent an increased risk of obesity and non-communicable diseases in children. *Obes Rev.* 2019;20 Suppl 1:5-17. PMID: 31419048	Population

Articles	Reason for Exclusion
169. Jebeile H, Gow ML, Baur LA, Garnett SP, Paxton SJ, Lister NB. Association of Pediatric Obesity Treatment, Including a Dietary Component, With Change in Depression and Anxiety: A Systematic Review and Meta-analysis. *JAMA Pediatr.* 2019;173(11):e192841. PMID: 31524933	Population
170. Jebeile H, Gow ML, Baur LA, Garnett SP, Paxton SJ, Lister NB. Treatment of obesity, with a dietary component, and eating disorder risk in children and adolescents: A systematic review with meta-analysis. *Obes Rev.* 2019;20(9):1287-1298. PMID: 31131531	Population
171. Ji X, Grandner MA, Liu J. The relationship between micronutrient status and sleep patterns: a systematic review. *Public Health Nutr.* 2017;20(4):687-701. PMID: 27702409	Population
172. Jia P, Luo M, Li Y, Zheng JS, Xiao Q, Luo J. Fast-food restaurant, unhealthy eating, and childhood obesity: A systematic review and meta-analysis. *Obes Rev.* 2021;22 Suppl 1:e12944. PMID: 31507064	Study Design
173. Johnson BJ, Zarnowiecki D, Hendrie GA, Mauch CE, Golley RK. How to reduce parental provision of unhealthy foods to 3- to 8-year-old children in the home environment? A systematic review utilizing the Behaviour Change Wheel framework. *Obes Rev.* 2018;19(10):1359-1370. PMID: 30092606	Outcome
174. Johnson RK, Lichtenstein AH, Anderson CAM, et al. Low-Calorie Sweetened Beverages and Cardiometabolic Health: A Science Advisory From the American Heart Association. *Circulation.* 2018;138(9):e126-e140. PMID: 30354445	Study Design
175. Juhl CR, Bergholdt HKM, Miller IM, Jemec GBE, Kanters JK, Ellervik C. Dairy Intake and Acne Vulgaris: A Systematic Review and Meta-Analysis of 78,529 Children, Adolescents, and Young Adults. *Nutrients.* 2018;10(8). PMID: 30096883	SR does not have 2 databases or ROB assessment
176. Kaakinen P, Kyngas H, Kaariainen M. Technology-based counseling in the management of weight and lifestyles of obese or overweight children and adolescents: A descriptive systematic literature review. *Inform Health Soc Care.* 2018;43(2):126-141. PMID: 28829655	Population
177. Kagie R, Lin SN, Hussain MA, Thompson SC. A Pragmatic Review to Assist Planning and Practice in Delivering Nutrition Education to Indigenous Youth. *Nutrients.* 2019;11(3). PMID: 30818853	SR does not have 2 databases or ROB assessment
178. Kang K, Sotunde OF, Weiler HA. Effects of Milk and Milk-Product Consumption on Growth among Children and Adolescents Aged 6-18 Years: A Meta-Analysis of Randomized Controlled Trials. *Adv Nutr.* 2019;10(2):250-261. PMID: 30839054	Outcome

Articles	Reason for Exclusion
179. Kastorini CM, Critselis E, Zota D, et al. National Dietary Guidelines of Greece for children and adolescents: a tool for promoting healthy eating habits. *Public Health Nutr.* 2019;22(14):2688-2699. PMID: 31111809	Study Design
180. Kemper AR, Fan T, Grossman DC, Phipps MG. Gaps in evidence regarding iron deficiency anemia in pregnant women and young children: summary of US Preventive Services Task Force recommendations. *Am J Clin Nutr.* 2017;106(Suppl 6):1555S-1558S. PMID: 29070541	Population
181. Kent K, Charlton KE, Netzel M, Fanning K. Food-based anthocyanin intake and cognitive outcomes in human intervention trials: a systematic review. *J Hum Nutr Diet.* 2017;30(3):260-274. PMID: 27730693	SR does not have 2 databases or ROB assessment
182. Khan K, Jovanovski E, Ho HVT, et al. The effect of viscous soluble fiber on blood pressure: A systematic review and meta-analysis of randomized controlled trials. *Nutr Metab Cardiovasc Dis.* 2018;28(1):3-13. PMID: 29153856	Population
183. Khanna P, Chattu VK, Aeri BT. Nutritional Aspects of Depression in Adolescents - A Systematic Review. *Int J Prev Med.* 2019;10:42. PMID: 31057727	SR does not have 2 databases or ROB assessment
184. King S, Tancredi D, Lenoir-Wijnkoop I, et al. Does probiotic consumption reduce antibiotic utilization for common acute infections? A systematic review and meta-analysis. *Eur J Public Health.* 2019;29(3):494-499. PMID: 30219897	Population
185. Kirkland L, Apatu E, Mease A, Largo-Wight E. School-based nutrition and garden programs and parental dietary changes in low-income settings: a review. *International Journal of Health Promotion and Education.* 2018;56(4-5):226-236. DOI: 10.1080/14635240.2018.1512881	SR does not have 2 databases or ROB assessment
186. Kao TA, Ling J, Hawn R, Vu C. The effects of motivational interviewing on children's body mass index and fat distributions: A systematic review and meta-analysis. Obes Rev. 2021;22(10):e13308.	No nutrition intervention of interest
187. Kemp BJ, Thompson DR, Watson CJ, McGuigan K, Woodside JV, Ski CF. Effectiveness of family-based eHealth interventions in cardiovascular disease risk reduction: A systematic review. Prev Med. 2021;149:106608.	No nutrition intervention of interest
188. Kininmonth AR, Smith AD, Llewellyn CH, Dye L, Lawton CL, Fildes A. The relationship between the home environment and child adiposity: a systematic review. International Journal of Behavioral Nutrition & Physical Activity. 2021;18(1):1-20.	No nutrition intervention of interest

Articles	Reason for Exclusion
189. Koletzko B, Godfrey KM, Poston L, et al. Nutrition During Pregnancy, Lactation and Early Childhood and its Implications for Maternal and Long-Term Child Health: The Early Nutrition Project Recommendations. *Ann Nutr Metab.* 2019;74(2):93-106. PMID: 30673669	Population
190. Kosmeri C, Siomou E, Vlahos AP, Milionis H. Review shows that lipid disorders are associated with endothelial but not renal dysfunction in children. *Acta Paediatr.* 2019;108(1):19-27. PMID: 30066344	SR does not have 2 databases or ROB assessment
191. Kraak VI, Rincon-Gallardo Patino S, Sacks G. An accountability evaluation for the International Food & Beverage Alliance's Global Policy on Marketing Communications to Children to reduce obesity: A narrative review to inform policy. *Obes Rev.* 2019;20 Suppl 2:90-106. PMID: 31034139	SR does not have 2 databases or ROB assessment
192. Kracht CL, Burkart S, Flanagan EW, Melnick E, Luecking C, Neshteruk C. Policy, system, and environmental interventions addressing obesity and diet-related outcomes in early childhood education settings: A systematic review. Obes Rev. 2023;24(4):e13547.	No nutrition intervention of interest
193. Kranz S, Pimpin L, Fawzi W, Duggan C, Webb P, Mozaffarian D. Mortality Benefits of Vitamin A Are Not Affected by Varying Frequency, Total Dose, or Duration of Supplementation. *Food Nutr Bull.* 2017;38(2):260-266. PMID: 28513263	SR does not have 2 databases or ROB assessment
194. Labonte ME, Poon T, Gladanac B, et al. Nutrient Profile Models with Applications in Government-Led Nutrition Policies Aimed at Health Promotion and Noncommunicable Disease Prevention: A Systematic Review. *Adv Nutr.* 2018;9(6):741-788. PMID: 30462178	SR does not have 2 databases or ROB assessment
195. Lamas S, Rebelo S, da Costa S, Sousa H, Zagalo N, Pinto E. The Influence of Serious Games in the Promotion of Healthy Diet and Physical Activity Health: A Systematic Review. Nutrients. 2023;15(6).	Outcome
196. Lassale C, Batty GD, Baghdadli A, et al. Healthy dietary indices and risk of depressive outcomes: a systematic review and meta-analysis of observational studies. *Mol Psychiatry.* 2019;24(7):965-986. PMID: 30254236	Population
197. Lassale C, Fitó M, Morales-Suárez-Varela M, Moya A, Gómez SF, Schröder H. Mediterranean diet and adiposity in children and adolescents: A systematic review. Obes Rev. 2022;23 Suppl 1:e13381.	SR does not have 2 databases or ROB assessment
198. Lassi ZS, Salam RA, Bhutta ZA. Recommendations on Arresting Global Health Challenges Facing Adolescents and Young Adults. *Ann Glob Health.* 2017;83(5-6):704-712. PMID: 29248085	Study Design

Articles	Reason for Exclusion
199. Lecube A, Monereo S, Rubio MA, et al. Prevention, diagnosis, and treatment of obesity. 2016 position statement of the Spanish Society for the Study of Obesity. *Endocrinol Diabetes Nutr.* 2017;64 Suppl 1:15-22. PMID: 27543006	Population
200. Lemale J, Mas E, Jung C, et al. Vegan diet in children and adolescents. Recommendations from the French-speaking Pediatric Hepatology, Gastroenterology and Nutrition Group (GFHGNP). *Arch Pediatr.* 2019;26(7):442-450. PMID: 31615715	Study Design
201. Leme ACB, Haines J, Tang L, et al. Impact of Strategies for Preventing Obesity and Risk Factors for Eating Disorders among Adolescents: A Systematic Review. *Nutrients.* 2020;12(10). PMID: 33066501	No meta-analysis or graded certainty of evidence
202. Lewis KA, Brown SA. Searching for Evidence of an Anti-Inflammatory Diet in Children: A Systematic Review of Randomized Controlled Trials for Pediatric Obesity Interventions With a Focus on Leptin, Ghrelin, and Adiponectin. *Biol Res Nurs.* 2017;19(5):511-530. PMID: 28743192	Population
203. Li B, Lv J, Wang W, Zhang D. Dietary magnesium and calcium intake and risk of depression in the general population: A meta-analysis. *Aust N Z J Psychiatry.* 2017;51(3):219-229. PMID: 27807012	SR does not have 2 databases or ROB assessment
204. Liberali R, Kupek E, Assis MAA. Dietary Patterns and Childhood Obesity Risk: A Systematic Review. *Child Obes.* 2020;16(2):70-85. PMID: 31742427	Study Design
205. Lim YM, Song S, Song WO. Prevalence and Determinants of Overweight and Obesity in Children and Adolescents from Migrant and Seasonal Farmworker Families in the United States-A Systematic Review and Qualitative Assessment. *Nutrients.* 2017;9(3). PMID: 28245565	Study Design
206. Lima do Vale MR, Farmer A, Ball GDC, Gokiert R, Maximova K, Thorlakson J. Implementation of Healthy Eating Interventions in Center-Based Childcare: The Selection, Application, and Reporting of Theories, Models, and Frameworks. *Am J Health Promot.* 2020;34(4):402-417. PMID: 31983219	SR does not have 2 databases or ROB assessment
207. Limone P, Messina G, Toto GA. Serious games and eating behaviors: A systematic review of the last 5 years (2018-2022). Front Nutr. 2022;9:978793.	Outcome
208. Lindsay AC, Mesa T, Greaney ML, Wallington SF, Wright JA. Associations Between Maternal Depressive Symptoms and Nonresponsive Feeding Styles and Practices in Mothers of Young Children: A Systematic Review. *JMIR Public Health Surveill.* 2017;3(2):e29. PMID: 28550007	No nutrition intervention of interest

Articles	Reason for Exclusion
209. Litchford A, Savoie Roskos MR, Wengreen H. Influence of fathers on the feeding practices and behaviors of children: A systematic review. *Appetite.* 2020;147:104558. PMID: 31870933	No meta-analysis or graded certainty of evidence
210. Liu E, Pimpin L, Shulkin M, et al. Effect of Zinc Supplementation on Growth Outcomes in Children under 5 Years of Age. *Nutrients.* 2018;10(3). PMID: 29558383	No nutrition intervention of interest
211. Liu YS, Wu QJ, Xia Y, et al. Carbohydrate intake and risk of metabolic syndrome: A dose-response meta-analysis of observational studies. *Nutr Metab Cardiovasc Dis.* 2019;29(12):1288-1298. PMID: 31653521	Population
212. Locke A, Stoesser K, Pippitt K. Health Maintenance in School-Aged Children: Part II. Counseling Recommendations. *Am Fam Physician.* 2019;100(4):219-226. PMID: 31414773	Study Design
213. Lopez PD, Cativo EH, Atlas SA, Rosendorff C. The Effect of Vegan Diets on Blood Pressure in Adults: A Meta-Analysis of Randomized Controlled Trials. Am J Med. 2019;132(7):875-883.e877.	Population
214. Lott M, Callahan E, Welker Duffy E, Story M, Daniels S. *Healthy Beverage Consumption in Early Childhood: Recommendations from Key National Health and Nutrition Organizations. Technical Scientific Report.* Durham, NC: Healthy Eating Research;2019.	Study Design
215. Luecking CT, Hennink-Kaminski H, Ihekweazu C, Vaughn A, Mazzucca S, Ward DS. Social marketing approaches to nutrition and physical activity interventions in early care and education centres: a systematic review. *Obes Rev.* 2017;18(12):1425-1438. PMID: 28960764	SR does not have 2 databases or ROB assessment
216. MacArthur G, Caldwell DM, Redmore J, et al. Individual-, family-, and school-level interventions targeting multiple risk behaviours in young people. *Cochrane Database Syst Rev.* 2018;10:CD009927. PMID: 30288738	No nutrition intervention of interest
217. Macedo RCO, Vieira AF, Moritz CEJ, Reischak-Oliveira A. Effects of fructose consumption on postprandial TAG: an update on systematic reviews with meta-analysis. *Br J Nutr.* 2018;120(4):364-372. PMID:29962368	Population
218. Mack I, Bayer C, Schaffeler N, et al. Chances and Limitations of Video Games in the Fight against Childhood Obesity-A Systematic Review. *Eur Eat Disord Rev.* 2017;25(4):237-267. PMID: 28467004	No meta-analysis or graded certainty of evidence
219. Maine A, Brown MJ, Ski CF, Thompson DR, Marsh L, O'Leary L. Recruitment settings, delivery contexts, intervention techniques and outcomes of health promotion programmes for young adults with intellectual and developmental disabilities: A systematic review. *Res Dev Disabil.* 2020;99:103592. PMID: 32035320	No meta-analysis or graded certainty of evidence

Articles	Reason for Exclusion
220. Manley J, Alderman H, Gentilini U. More evidence on cash transfers and child nutritional outcomes: a systematic review and meta-analysis. BMJ Glob Health. 2022;7(4).	SR does not have 2 databases or ROB assessment
221. Mann JP, Tang GY, Nobili V, Armstrong MJ. Evaluations of Lifestyle, Dietary, and Pharmacologic Treatments for Pediatric Nonalcoholic Fatty Liver Disease: A Systematic Review. *Clin Gastroenterol Hepatol.* 2019;17(8):1457-1476 e1457. PMID: 29857146	Population
222. Mansfield JL, Savaiano DA. Effect of school wellness policies and the Healthy, Hunger-Free Kids Act on food-consumption behaviors of students, 2006-2016: a systematic review. *Nutr Rev.* 2017;75(7):533-552. PMID: 28838082	Outcome
223. Marcano-Olivier MI, Horne PJ, Viktor S, Erjavec M. Using Nudges to Promote Healthy Food Choices in the School Dining Room: A Systematic Review of Previous Investigations. *J Sch Health.* 2020;90(2):143-157. PMID: 31852016	Outcome
224. Martin A, Booth JN, Laird Y, Sproule J, Reilly JJ, Saunders DH. Physical activity, diet and other behavioural interventions for improving cognition and school achievement in children and adolescents with obesity or overweight. *Cochrane Database Syst Rev.* 2018;3:CD009728. PMID: 29499084	Population
225. Martínez-Rodríguez A, Cumbicus-Jiménez Y, Cuestas-Calero BJ, Leyva-Vela B. Nutrition and Boxing Performance. *Nutrition today.* 2017;52(6):295-307. DOI: vdc.100052191563.0x000001	Population
226. Matson RI, Perry R, Hunt LP, et al. Change in obesity-related metabolic abnormalities associated with body mass index improvement through life-style intervention: A meta-regression. *Pediatr Diabetes.* 2020;21(2):173-193. PMID: 31820534	Population
227. Matsuyama M, Harb T, David M, Davies PS, Hill RJ. Effect of fortified milk on growth and nutritional status in young children: a systematic review and meta-analysis. *Public Health Nutr.* 2017;20(7):1214-1225. PMID: 27938461	Population
228. Matsuzaki M, Sanchez BN, Acosta ME, Botkin J, Sanchez-Vaznaugh EV. Food environment near schools and body weight-A systematic review of associations by race/ethnicity, gender, grade, and socio-economic factors. *Obes Rev.* 2020;21(4):e12997. PMID: 32026567	No meta-analysis or graded certainty of evidence
229. Matwiejczyk L, Mehta K, Scott J, Tonkin E, Coveney J. Characteristics of Effective Interventions Promoting Healthy Eating for Pre-Schoolers in Childcare Settings: An Umbrella Review. *Nutrients.* 2018;10(3). PMID: 29494537	Study Design

Articles	Reason for Exclusion
230. Mayer-Davis EJ, Kahkoska AR, Jefferies C, et al. ISPAD Clinical Practice Consensus Guidelines 2018: Definition, epidemiology, and classification of diabetes in children and adolescents. *Pediatr Diabetes.* 2018;19 Suppl 27:7-19. PMID: 30226024	Study Design
231. Mazur A, Caroli M, Radziewicz-Winnicki I, et al. Reviewing and addressing the link between mass media and the increase in obesity among European children: The European Academy of Paediatrics (EAP) and The European Childhood Obesity Group (ECOG) consensus statement. *Acta Paediatr.* 2018;107(4):568-576. PMID: 29164673	Study Design
232. McHugh C, Hurst A, Bethel A, Lloyd J, Logan S, Wyatt K. The impact of the World Health Organization Health Promoting Schools framework approach on diet and physical activity behaviours of adolescents in secondary schools: a systematic review. Public health. 2020;182:116-124.	No meta-analysis or graded certainty of evidence
233. Mead E, Brown T, Rees K, et al. Diet, physical activity and behavioural interventions for the treatment of overweight or obese children from the age of 6 to 11 years. *Cochrane Database Syst Rev.* 2017;6:CD012651. PMID: 28639319	Population
234. Mead LC, Hill AM, Carter S, Coates AM. The Effect of Nut Consumption on Diet Quality, Cardiometabolic and Gastrointestinal Health in Children: A Systematic Review of Randomized Controlled Trials. *Int J Environ Res Public Health.* 2021;18(2). PMID: 33430029	No nutrition intervention of interest
235. Mehrabani S, Askari G, Miraghajani M, Tavakoly R, Arab A. Effect of coenzyme Q10 supplementation on fatigue: A systematic review of interventional studies. *Complement Ther Med.* 2019;43:181-187. PMID: 30935528	Population
236. Mena-Sanchez G, Becerra-Tomas N, Babio N, Salas-Salvado J. Dairy Product Consumption in the Prevention of Metabolic Syndrome: A Systematic Review and Meta-Analysis of Prospective Cohort Studies. *Adv Nutr.* 2019;10(suppl_2):S144-S153. PMID: 31089736	Population
237. Merhej R. Dehydration and cognition: an understated relation. *International Journal of Health Governance.* 2018;24(1):19-30. DOI: 10.1108/IJHG-10-2018-0056	SR does not have 2 databases or ROB assessment
238. Micha R, Karageorgou D, Bakogianni I, et al. Effectiveness of school food environment policies on children's dietary behaviors: A systematic review and meta-analysis. *PLoS One.* 2018;13(3):e0194555. PMID: 29596440	Outcome
239. Militello LK, Kelly S, Melnyk BM, Smith L, Petosa R. A Review of Systematic Reviews Targeting the Prevention and Treatment of Overweight and Obesity in Adolescent Populations. *J Adolesc Health.* 2018;63(6):675-687. PMID: 30314864	Study Design

Articles	Reason for Exclusion
240. Montgomery SC, Donnelly M, Bhatnagar P, Carlin A, Kee F, Hunter RF. Peer social network processes and adolescent health behaviors: A systematic review. *Prev Med.* 2020;130:105900. PIMD: 31733224	Population
241. Monzani A, Ricotti R, Caputo M, et al. A Systematic Review of the Association of Skipping Breakfast with Weight and Cardiometabolic Risk Factors in Children and Adolescents. What Should We Better Investigate in the Future? *Nutrients.* 2019;11(2). PMID: 30781797	Study Design
242. Moore SG, Donnelly JK, Jones S, Cade JE. Effect of Educational Interventions on Understanding and Use of Nutrition Labels: A Systematic Review. *Nutrients.* 2018;10(10). PMID: 30287766	Population
243. Moreno JP, Vezina-Im LA, Vaughan EM, Baranowski T. Impact of child summertime obesity interventions on body mass index, and weight-related behaviours: a systematic review and meta-analysis protocol. *BMJ Open.* 2017;7(10):e017144. PMID: 29061614	Study Design
244. Morgan PJ, Young MD, Lloyd AB, et al. Involvement of Fathers in Pediatric Obesity Treatment and Prevention Trials: A Systematic Review. *Pediatrics.* 2017;139(2). PMID: 28130430	SR does not have 2 databases or ROB assessment
245. Moynihan P, Tanner LM, Holmes RD, et al. Systematic Review of Evidence Pertaining to Factors That Modify Risk of Early Childhood Caries. *JDR Clin Trans Res.* 2019;4(3):202-216. PMID: 30931717	Outcome
246. Murimi MW, Kanyi M, Mupfudze T, Amin MR, Mbogori T, Aldubayan K. Factors Influencing Efficacy of Nutrition Education Interventions: A Systematic Review. *J Nutr Educ Behav.* 2017;49(2):142-165 e141. PMID: 27814976	Population
247. Murimi MW, Moyeda-Carabaza AF, Nguyen B, Saha S, Amin R, Njike V. Factors that contribute to effective nutrition education interventions in children: a systematic review. *Nutr Rev.* 2018;76(8):553-580. PMID: 29800311	No meta-analysis or graded certainty of evidence
248. Murray M, Dordevic AL, Bonham MP. Systematic Review and Meta-Analysis: The Impact of Multicomponent Weight Management Interventions on Self-Esteem in Overweight and Obese Adolescents. *J Pediatr Psychol.* 2017;42(4):379-394. PMID:28340156	Population
249. Musa-Veloso K, Venditti C, Lee HY, et al. Systematic review and meta-analysis of controlled intervention studies on the effectiveness of long-chain omega-3 fatty acids in patients with nonalcoholic fatty liver disease. *Nutr Rev.* 2018;76(8):581-602. PMID: 29917092	Population

Articles	Reason for Exclusion
250. Muth ND, Dietz WH, Magge SN, et al. Public Policies to Reduce Sugary Drink Consumption in Children and Adolescents. *Pediatrics.* 2019;143(4). PMID: 30910915	Study Design
251. Muzaffar H, Metcalfe JJ, Fiese B. Narrative Review of Culinary Interventions with Children in Schools to Promote Healthy Eating: Directions for Future Research and Practice. *Curr Dev Nutr.* 2018;2(6):nzy016. PMID: 29955728	No meta-analysis or graded certainty of evidence
252. Narciso J, Silva AJ, Roderigues V, et al. Behavioral, contextual and biological factors associated with obesity during adolescence: A systematic review. *PLoS ONE* 2019;14(4):e0214941.	Study Design
253. Nathan N, Janssen L, Sutherland R, et al. The effectiveness of lunchbox interventions on improving the foods and beverages packed and consumed by children at centre-based care or school: a systematic review and meta-analysis. *Int J Behav Nutr Phys Act.* 2019;16(1):38. PMID: 31036038	Outcome
254. Naude CE, Visser ME, Nguyen KA, Durao S, Schoonees A. Effects of total fat intake on bodyweight in children. *Cochrane Database Syst Rev.* 2018;7:CD012960. PMID: 29974953	No nutrition intervention of interest
255. Naumann J, Biehler D, Luty T, Sadaghiani C. Prevention and Therapy of Type 2 Diabetes-What Is the Potential of Daily Water Intake and Its Mineral Nutrients? *Nutrients.* 2017;9(8). PMID: 28829398	SR does not have 2 databases or ROB assessment
256. Nekitsing C, Blundell-Birtill P, Cockroft JE, Hetherington MM. Systematic review and meta-analysis of strategies to increase vegetable consumption in preschool children aged 2-5 years. *Appetite.* 2018;127:138-154. PMID: 29702128	Outcome
257. Newberry SJ, Chung M, Anderson C A.M., et al. *Sodium and Potassium Intake: Effects on Chronic Disease Outcomes and Risks.* Rockville (MD): Agency for Healthcare Research and Quality (US); June 2018. PMID: 30125063	Outcome
258. Nikbakht E, Khalesi S, Singh I, Williams LT, West NP, Colson N. Effect of probiotics and synbiotics on blood glucose: a systematic review and meta-analysis of controlled trials. *Eur J Nutr.* 2018;57(1):95-106. PMID: 27590729	Population
259. Nissensohn M, Fuentes Lugo D, Serra-Majem L. Sugar-sweetened beverage consumption and obesity in children's meta-analyses: reaching wrong answers for right questions. *Nutr Hosp.* 2018;35(2):474-488. PMID: 29756984	Study Design
260. Nissensohn M, Fuentes Lugo D, Serra-Majem L. Sugar-sweetened beverage consumption and obesity in children's meta-analyses: reaching wrong answers for right questions. *Nutr Hosp.* 2018;35(2):474-488. PMID: 29756984	Other

Articles	Reason for Exclusion
261. Noronha JC, Braunstein CR, Blanco Mejia S, et al. The Effect of Small Doses of Fructose and Its Epimers on Glycemic Control: A Systematic Review and Meta-Analysis of Controlled Feeding Trials. *Nutrients.* 2018;10(11). PMID: 30463314	Population
262. Nour M, Yeung SH, Partridge S, Allman-Farinelli M. A Narrative Review of Social Media and Game-Based Nutrition Interventions Targeted at Young Adults. *J Acad Nutr Diet.* 2017;117(5):735-752 e710. PMID: 28238894	Population
263. Omiwole M, Richardson C, Huniewicz P, Dettmer E, Paslakis G. Review of Mindfulness-Related Interventions to Modify Eating Behaviors in Adolescents. *Nutrients.* 2019;11(12). PMID: 31810307	SR does not have 2 databases or ROB assessment
264. Ong JX, Ullah S, Magarey A, Miller J, Leslie E. Relationship between the home environment and fruit and vegetable consumption in children aged 6-12 years: a systematic review. *Public Health Nutr.* 2017;20(3):464-480. PMID: 2869044	Outcome
265. Onvani S, Haghighatdoost F, Surkan PJ, Larijani B, Azadbakht L. Adherence to the Healthy Eating Index and Alternative Healthy Eating Index dietary patterns and mortality from all causes, cardiovascular disease and cancer: a meta-analysis of observational studies. *J Hum Nutr Diet.* 2017;30(2):216-226. PMID: 27620213	Population
266. Oosterhoff M, Bosma H, van Schayck OCP, Evers S, Dirksen CD, Joore MA. A systematic review on economic evaluations of school-based lifestyle interventions targeting weight-related behaviours among 4-12year olds: Issues and ways forward. *Prev Med.* 2018;114:115-122. PMID: 29959951	SR does not have 2 databases or ROB assessment
267. Opie RS, Itsiopoulos C, Parletta N, et al. Dietary recommendations for the prevention of depression. *Nutr Neurosci.* 2017;20(3):161-171. PMID: 26317148	Study Design
268. O'Sullivan TA, Schmidt KA, Kratz M. Whole-Fat or Reduced-Fat Dairy Product Intake, Adiposity, and Cardiometabolic Health in Children: A Systematic Review. *Adv Nutr.* 2020;11(4):928-950. PMID: 32119732	SR does not have 2 databases or ROB assessment
269. Parohan M, Sadeghi A, Nasiri M, et al. Dietary acid load and risk of hypertension: A systematic review and dose-response meta-analysis of observational studies. *Nutr Metab Cardiovasc Dis.* 2019;29(7):665-675. PMID: 31153745	Population
270. Patel AI, Moghadam SD, Freedman M, Hazari A, Fang ML, Allen IE. The association of flavored milk consumption with milk and energy intake, and obesity: A systematic review. *Prev Med.* 2018;111:151-162. PMID: 29501475	Outcome

Articles	Reason for Exclusion
271. Patel C, Karasouli E, Shuttlewood E, Meyer C. Food Parenting Practices among Parents with Overweight and Obesity: A Systematic Review. *Nutrients.* 2018;10(12). PMID: 30545102	Population
272. Patseadou M, Haller DM. Vitamin D in Adolescents: A Systematic Review and Narrative Synthesis of Available Recommendations. *J Adolesc Health.* 2020;66(4):388-407. PMID: 31685374	SR does not have 2 databases or ROB assessment
273. Paula Bricarello L, Poltronieri F, Fernandes R, Retondario A, de Moraes Trindade EBS, de Vasconcelos FAG. Effects of the Dietary Approach to Stop Hypertension (DASH) diet on blood pressure, overweight and obesity in adolescents: A systematic review. *Clin Nutr ESPEN.* 2018;28:1-11. PMID: 30390863	Study Design
274. Pawlak R, Bell K. Iron Status of Vegetarian Children: A Review of Literature. *Ann Nutr Metab.* 2017;70(2):88-99. PMID: 28319940	SR does not have 2 databases or ROB assessment
275. Payne JE, Turk MT, Kalarchian MA, Pellegrini CA. Defining Adherence to Dietary Self-Monitoring Using a Mobile App: A Narrative Review. *J Acad Nutr Diet.* 2018;118(11):2094-2119. PMID: 30115555	Population
276. Pena-Rosas JP, Mithra P, Unnikrishnan B, et al. Fortification of rice with vitamins and minerals for addressing micronutrient malnutrition. *Cochrane Database Syst Rev.* 2019;2019(10). PMID: 31684687	Other
277. Perez-Cueto FJA. An Umbrella Review of Systematic Reviews on Food Choice and Nutrition Published between 2017 and-2019. *Nutrients.* 2019;11(10). PMID: 31591373	SR does not have 2 databases or ROB assessment
278. Pfeiffle S, Pellegrino F, Kruseman M, et al. Current Recommendations for Nutritional Management of Overweight and Obesity in Children and Adolescents: A Structured Framework. *Nutrients.* 2019;11(2). PMID: 30744122	Population
279. Pfinder M, Heise TL, Hilton Boon M, et al. Taxation of unprocessed sugar or sugar-added foods for reducing their consumption and preventing obesity or other adverse health outcomes. *Cochrane Database Syst Rev.* 2020;4:CD012333. PMID: 32270494	Population
280. Piccoli de Mello P, Eifer DA, Daniel de Mello E. Use of fibers in childhood constipation treatment: systematic review with meta-analysis. *J Pediatr (Rio J).* 2018;94(5):460-470. PMID: 29474804	Outcome

Articles	Reason for Exclusion
281. Pihoker C, Forsander G, Fantahun B, et al. ISPAD Clinical Practice Consensus Guidelines 2018: The delivery of ambulatory diabetes care to children and adolescents with diabetes. *Pediatr Diabetes.* 2018;19 Suppl 27:84-104. PMID: 30144259	Study Design
282. Pinto de Jesus MC, Souza Braga VA, da Silva Pinheiro AP, et al. ACTIONS OF NURSES DIRECTED AT OBESE TEENS IN PRIMARY CARE. *Journal of Nursing UFPE.* 2019;13:729-739. DOI: 10.5205/1981-8963.2019.240871	SR does not have 2 databases or ROB assessment
283. Policy on Dietary Recommendations for Infants, Children, and Adolescents. *Pediatr Dent.* 2018;40(6):65-67. PMID: 32074854	Study Design
284. Policy on Snacks and Beverages Sold in Schools. *Pediatr Dent.* 2018;40(6):68-69. PMID: 32074855	Study Design
285. Policy on Use of Fluoride. *Pediatr Dent.* 2018;40(6):49-50. PMID: 32074848	Study Design
286. Pongutta S, Ajetunmobi O, Davey C, Ferguson E, Lin L. Impacts of School Nutrition Interventions on the Nutritional Status of School-Aged Children in Asia: A Systematic Review and Meta-Analysis. Nutrients. 2022;14(3).	Population
287. Porter A, Kipping R, Summerbell C, Dobrescu A, Johnson L. What guidance is there on portion size for feeding preschool-aged children (1 to 5 years) in the United Kingdom and Ireland? A systematic grey literature review. *Obes Rev.* 2020;21(7):e13021. PMID: 32219990	Study Design
288. Pourabbasi A, Ebrahimnegad Shirvani MS, Shams AH. Does Islamic fasting affect cognitive functions in adolescents? A systematic review. *J Pak Med Assoc.* 2019;69(8):1164-1169. PMID: 31431772	Outcome
289. Pratt CA, Loria CM, Arteaga SS, et al. A Systematic Review of Obesity Disparities Research. *Am J Prev Med.* 2017;53(1):113-122. PMID: 28341221	SR does not have 2 databases or ROB assessment
290. Prescott MP, Cleary R, Bonanno A, Costanigro M, Jablonski BBR, Long AB. Farm to School Activities and Student Outcomes: A Systematic Review. *Adv Nutr.* 2020;11(2):357-374. PMID: 32173754	No meta-analysis or graded certainty of evidence

Articles	Reason for Exclusion
291. Price C, Cohen D, Pribis P, Cerami J. Nutrition Education and Body Mass Index in Grades K-12: A Systematic Review. *J Sch Health.* 2017;87(9):715-720. PMID: 28766320	SR does not have 2 databases or ROB assessment
292. Psaltopoulou T, Tzanninis S, Ntanasis-Stathopoulos I, et al. Prevention and treatment of childhood and adolescent obesity: a systematic review of meta-analyses. *World J Pediatr.* 2019;15(4):350-381. PMID: 31313240	SR does not have 2 databases or ROB assessment
293. Quelly SB. Helping With Meal Preparation and Children's Dietary Intake: A Literature Review. *J Sch Nurs.* 2019;35(1):51-60. PMID: 29895188	SR does not have 2 databases or ROB assessment
294. Qutteina Y, De Backer C, Smits T. Media food marketing and eating outcomes among pre-adolescents and adolescents: A systematic review and meta-analysis. *Obes Rev.* 2019;20(12):1708-1719. PMID: 31468652	Outcome
295. Rageliene T, Gronhoj A. The influence of peers' and siblings' on children's and adolescents' healthy eating behavior. A systematic literature review. *Appetite.* 2020;148:104592. PMID: 31927070	No nutrition intervention of interest
296. Raine KD, Atkey K, Olstad DL, et al. Healthy food procurement and nutrition standards in public facilities: evidence synthesis and consensus policy recommendations. *Health Promot Chronic Dis Prev Can.* 2018;38(1):6-17. PMID: 29323862	Study Design
297. Rajjo T, Mohammed K, Alsawas M, et al. Treatment of Pediatric Obesity: An Umbrella Systematic Review. *J Clin Endocrinol Metab.* 2017;102(3):763-775. PMID: 28359101	Population
298. Ranchordas MK, Rogerson D, Soltani H, Costello JT. Antioxidants for preventing and reducing muscle soreness after exercise. *Cochrane Database Syst Rev.* 2017;12:CD009789. PMID: 29238948	Population
299. Rangel-Huerta OD, Gil A. Effect of omega-3 fatty acids on cognition: an updated systematic review of randomized clinical trials. *Nutr Rev.* 2018;76(1):1-20. PMID: 29240924	SR does not have 2 databases or ROB assessment
300. Reale S, Hamilton J, Akparibo R, Hetherington MM, Cecil JE, Caton SJ. The effect of food type on the portion size effect in children aged 2-12 years: A systematic review and meta-analysis. *Appetite.* 2019;137:47-61. PMID: 30779929	Outcome
301. Redondo M, Hernandez-Aguado I, Lumbreras B. The impact of the tax on sweetened beverages: a systematic review. *Am J Clin Nutr.* 2018;108(3):548-563. PMID: 30535085	Population

Articles	Reason for Exclusion
302. Rhee EJ, Kim HC, Kim JH, et al. 2018 Guidelines for the management of dyslipidemia. *Korean J Intern Med.* 2019;34(4):723-771. PMID: 31272142	Study Design
303. Ribeiro de Vasconcelos CM, Ribeiro de Vasconcelos EM, Gorete Lucena de Vasconcelos M, et al. EDUCATIONAL INTERVENTIONS IN THE PROMOTION OF HEALTHY EATING IN SCHOOLS. *Journal of Nursing UFPE.* 2018;12(10):2803-2815. DOI: 10.5205/1981-8963-v12i10a237682p2803-2815-2018	SR does not have 2 databases or ROB assessment
304. Roberts JL, Stein AD. The Impact of Nutritional Interventions beyond the First 2 Years of Life on Linear Growth: A Systematic Review and Meta-Analysis. *Adv Nutr.* 2017;8(2):323-336. PMID: 28298275	Outcome
305. Robson SM, McCullough MB, Rex S, Munafo MR, Taylor G. Family Meal Frequency, Diet, and Family Functioning: A Systematic Review With Meta-analyses. *J Nutr Educ Behav.* 2020;52(5):553-564. PMID: 31982371	Study Design
306. Rocha NP, Milagres LC, Longo GZ, Ribeiro AQ, Novaes JF. Association between dietary pattern and cardiometabolic risk in children and adolescents: a systematic review. *J Pediatr (Rio J).* 2017;93(3):214-222. PMID: 28238682	SR does not have 2 databases or ROB assessment
307. Rodriguez Rocha NP, Kim H. eHealth Interventions for Fruit and Vegetable Intake: A Meta-Analysis of Effectiveness. *Health Educ Behav.* 2019;46(6):947-959. PMID: 31347403	Outcome
308. Rogers MA, Lemmen K, Kramer R, Mann J, Chopra V. Internet-Delivered Health Interventions That Work: Systematic Review of Meta-Analyses and Evaluation of Website Availability. *J Med Internet Res.* 2017;19(3):e90. PMID: 28341617	SR does not have 2 databases or ROB assessment
309. Rose T, Barker M, Maria Jacob C, et al. A Systematic Review of Digital Interventions for Improving the Diet and Physical Activity Behaviors of Adolescents. *J Adolesc Health.* 2017;61(6):669-677. PMID: 28822682	Outcome
310. Rosettie KL, Micha R, Cudhea F, et al. Comparative risk assessment of school food environment policies and childhood diets, childhood obesity, and future cardiometabolic mortality in the United States. *PLoS One.* 2018;13(7):e0200378. PMID: 29979761	Study Design
311. Rouhani MH, Rashidi-Pourfard N, Salehi-Abargouei A, Karimi M, Haghighatdoost F. Effects of Egg Consumption on Blood Lipids: A Systematic Review and Meta-Analysis of Randomized Clinical Trials. *J Am Coll Nutr.* 2018;37(2):99-110. PMID: 29111915	Population

Articles	Reason for Exclusion
312. Russell SJ, Croker H, Viner RM. The effect of screen advertising on children's dietary intake: A systematic review and meta-analysis. *Obes Rev.* 2019;20(4):554-568. PMID: 30576057	Outcome
313. Saavedra Dias R, Barros AN, Silva AJ, et al. The effect of school intervention programs on the body mass index of adolescents: a systematic review with meta-analysis. *Health Educ Res.* 2020;35(5):396-406. PMID: 32772070	No nutrition intervention of interest
314. Sacco J, Lillico HG, Chen E, Hobin E. The influence of menu labelling on food choices among children and adolescents: a systematic review of the literature. *Perspect Public Health.* 2017;137(3):173-181. PMID: 27436235	Outcome
315. Saini V, Jessel J, Iannaccone JA, Agnew C. Efficacy of functional analysis for informing behavioral treatment of inappropriate mealtime behavior: A systematic review and meta-analysis. *Behavioral interventions.* 2019;34(2):231-247. DOI: vdc.100083009162.0x000001	SR does not have 2 databases or ROB assessment
316. Santos JAR, Christoforou A, Trieu K, et al. Iodine fortification of foods and condiments, other than salt, for preventing iodine deficiency disorders. *Cochrane Database Syst Rev.* 2019;2:CD010734. PMID: 30746700	No nutrition intervention of interest
317. Savoie-Roskos MR, Wengreen H, Durward C. Increasing Fruit and Vegetable Intake among Children and Youth through Gardening-Based Interventions: A Systematic Review. *J Acad Nutr Diet.* 2017;117(2):240-250. PMID: 27964852	Outcome
318. Schaap R, Bessems K, Otten R, Kremers S, van Nassau F. Measuring implementation fidelity of school-based obesity prevention programmes: a systematic review. *Int J Behav Nutr Phys Act.* 2018;15(1):75. PMID: 30103764	No nutrition intervention of interest
319. Schneider BC, Dumith SC, Orlandi SP, Assuncao MCF. Diet and body fat in adolescence and early adulthood: a systematic review of longitudinal studies. *Cien Saude Colet.* 2017;22(5):1539-1552. PMID: 28538925	SR does not have 2 databases or ROB assessment
320. Schoeppe S, Alley S, Rebar AL, et al. Apps to improve diet, physical activity and sedentary behaviour in children and adolescents: a review of quality, features and behaviour change techniques. *Int J Behav Nutr Phys Act.* 2017;14(1):83. PMID: 28646889	No nutrition intervention of interest
321. Schurmann S, Kersting M, Alexy U. Vegetarian diets in children: a systematic review. *Eur J Nutr.* 2017;56(5):1797-1817. PMID: 28299420	SR does not have 2 databases or ROB assessment
322. Scott AM, Clark J, Julien B, et al. Probiotics for preventing acute otitis media in children. *Cochrane Database Syst Rev.* 2019;6:CD012941. PMID: 31210358	Outcome

Articles	Reason for Exclusion
323. Selvendran SS, Penney NC, Aggarwal N, Darzi AW, Purkayastha S. Treatment of Obesity in Young People-a Systematic Review and Meta-analysis. *Obes Surg.* 2018;28(8):2537-2549. PMID: 29796922	Population
324. Serra-Majem L, Raposo A, Aranceta-Bartrina J, et al. Ibero(-)American Consensus on Low- and No-Calorie Sweeteners: Safety, Nutritional Aspects and Benefits in Food and Beverages. *Nutrients.* 2018;10(7). PMID: 29941818	Other
325. Seward K, Finch M, Yoong SL, et al. Factors that influence the implementation of dietary guidelines regarding food provision in centre based childcare services: A systematic review. *Prev Med.* 2017;105:197-205. PMID: 28965755	SR does not have 2 databases or ROB assessment
326. Shahmirzadi MS, Barati L. Treatment of Nonalcoholic Fatty Liver Disease in Children: A Systematic Review. *Journal of Pediatrics Review.* 2018;6(2):21-28. DOI: 10.5812/jpr.11635	SR does not have 2 databases or ROB assessment
327. Shin Y, Kim SK, Lee M. Mobile phone interventions to improve adolescents' physical health: A systematic review and meta-analysis. Public Health Nurs. 2019;36(6):787-799.	No nutrition intervention of interest
328. Shirazi MG, Kazemi A, Kelishadi R, Mostafavi F. A Review on Determinants of Nutritional Behavior in Teenagers. *IRANIAN JOURNAL OF PEDIATRICS.* 2017;27(3). DOI: 10.5812/ijp.6454	SR does not have 2 databases or ROB assessment
329. Silden KE. Impact of competitive foods in public schools on child nutrition: effects on adolescent obesity in the United States an integrative systematic literature review. *Glob Health Action.* 2018;11(1):1477492. PMID: 29893188	Study Design
330. Silva Neto LGR, Santos Neto JED, Bueno NB, de Oliveira SL, Ataide TDR. Effects of iron supplementation versus dietary iron on the nutritional iron status: Systematic review with meta-analysis of randomized controlled trials. *Crit Rev Food Sci Nutr.* 2019;59(16):2553-2561. PMID: 29611716	Outcome
331. Singh A, Bassi S, Nazar GP, et al. Impact of school policies on non-communicable disease risk factors - a systematic review. BMC Public Health. 2017;17(1):292.	No meta-analysis or graded certainty of evidence
332. Smart CE, Annan F, Higgins LA, Jelleryd E, Lopez M, Acerini CL. ISPAD Clinical Practice Consensus Guidelines 2018: Nutritional management in children and adolescents with diabetes. *Pediatr Diabetes.* 2018;19 Suppl 27:136-154. PMID: 30062718	Study Design

Articles	Reason for Exclusion
333. Smith R, Kelly B, Yeatman H, Boyland E. Food Marketing Influences Children's Attitudes, Preferences and Consumption: A Systematic Critical Review. *Nutrients.* 2019;11(4). PMID: 31003489	Outcome
334. Snuggs S, Houston-Price C, Harvey K. Healthy eating interventions delivered in the family home: A systematic review. *Appetite.* 2019;140:114-133. PMID: 31091432	No meta-analysis or graded certainty of evidence
335. Soares MM, Silva MA, Garcia PPC, et al. Efect of vitamin A suplementation: a systematic review. *Cien Saude Colet.* 2019;24(3):827-838. PMID: 30892504	Population
336. Spill MK, Callahan EH, Shapiro MJ, et al. Caregiver feeding practices and child weight outcomes: a systematic review. *Am J Clin Nutr.* 2019;109(Suppl_7):990S-1002S. PMID: 30982865	Population
337. Srbely V, Janjua I, Buchholz AC, Newton G. Interventions Aimed at Increasing Dairy and/or Calcium Consumption of Preschool-Aged Children: A Systematic Literature Review. *Nutrients.* 2019;11(4). PMID: 30934755	Outcome
338. Stead M, Angus K, Langley T, et al. Mass media to communicate public health messages in six health topic areas: a systematic review and other reviews of the evidence. In: *Public Health Research.* Vol 7.8. Southampton (UK): NIHR Journals Library; 2019. PMID: 31046212	Population
339. St George SM, Kobayashi MA, Noriega Esquives BS, Ocasio MA, Wagstaff RG, Dorcius DP. Pediatric Obesity Prevention and Treatment Among Hispanics: A Systematic Review and Meta-Analysis. Am J Prev Med. 2022;62(3):438-449.	Population
340. Styne DM, Arslanian SA, Connor EL, et al. Pediatric Obesity-Assessment, Treatment, and Prevention: An Endocrine Society Clinical Practice Guideline. *J Clin Endocrinol Metab.* 2017;102(3):709-757. PMID: 28359099	Study Design
341. Suh H, Kavouras SA. Water intake and hydration state in children. *Eur J Nutr.* 2019;58(2):475-496. PMID: 30506317	Outcome
342. Suhett LG, Hermsdorff HHM, Cota BC, et al. Dietary inflammatory potential, cardiometabolic risk and inflammation in children and adolescents: a systematic review. *Crit Rev Food Sci Nutr.* 2021;61(3):407-416. PMID: 32156146	Study Design

Articles	Reason for Exclusion
343. Suthers R, Broom M, Beck E. Key Characteristics of Public Health Interventions Aimed at Increasing Whole Grain Intake: A Systematic Review. *J Nutr Educ Behav.* 2018;50(8):813-823. PMID: 30076022	Population
344. Sutter C, Metcalfe JJ, Tucker L, et al. Defining Food Education Standards through Consensus: The Pilot Light Food Education Summit. *J Sch Health.* 2019;89(12):994-1003. PMID: 31612490	Study Design
345. Swartz H. Produce Rx Programs for Diet-Based Chronic Disease Prevention. *AMA J Ethics.* 2018;20(10):E960-973. PMID: 30346924	SR does not have 2 databases or ROB assessment
346. Tabatabaei-Malazy O, Ardeshirlarijani E, Namazi N, Nikfar S, Jalili RB, Larijani B. Dietary antioxidative supplements and diabetic retinopathy; a systematic review. *J Diabetes Metab Disord.* 2019;18(2):705-716. PMID: 31890694	Population
347. Taghizadeh S, Farhangi MA. The effectiveness of pediatric obesity prevention policies: a comprehensive systematic review and dose-response meta-analysis of controlled clinical trials. *J Transl Med.* 2020;18(1):480. PMID: 33317542	No nutrition intervention of interest
348. Tahmassebi JF, BaniHani A. Impact of soft drinks to health and economy: a critical review. *Eur Arch Paediatr Dent.* 2020;21(1):109-117. PMID: 31177478	SR does not have 2 databases or ROB assessment
349. Tam R, Beck KL, Manore MM, Gifford J, Flood VM, O'Connor H. Effectiveness of Education Interventions Designed to Improve Nutrition Knowledge in Athletes: A Systematic Review. *Sports Med.* 2019;49(11):1769-1786. PMID: 31372860	Population
350. Tamayo MC, Dobbs PD, Pincu Y. Family-Centered Interventions for Treatment and Prevention of Childhood Obesity in Hispanic Families: A Systematic Review. *J Community Health.* 2021;46(3):635-643. PMID: 32734580	No meta-analysis or graded certainty of evidence
351. Te Morenga L, Montez JM. Health effects of saturated and trans-fatty acid intake in children and adolescents: Systematic review and meta-analysis. *PLoS One.* 2017;12(11):e0186672. PMID: 29149184	Population
352. Temiz G, Isil O. Factors Affecting Healthy Life Style Behaviors in Adolescents; Eating Disorders: A Systematic Review. *INT J CARING SCI.* 2018;11(3):1352-1361.	SR does not have 2 databases or ROB assessment

Articles	Reason for Exclusion
353. Teng M, Zhao YJ, Khoo AL, Yeo TC, Yong QW, Lim BP. Impact of coconut oil consumption on cardiovascular health: a systematic review and meta-analysis. *Nutr Rev.* 2020;78(3):249-259. PMID: 31769848	Population
354. Teoh SL, Lai NM, Vanichkulpitak P, Vuksan V, Ho H, Chaiyakunapruk N. Clinical evidence on dietary supplementation with chia seed (Salvia hispanica L.): a systematic review and meta-analysis. *Nutr Rev.* 2018;76(4):219-242. PMID: 29452425	Population
355. Thompson KL, Chung M, Handu D, et al. The Effectiveness of Nutrition Specialists on Pediatric Weight Management Outcomes in Multicomponent Pediatric Weight Management Interventions: A Systematic Review and Exploratory Meta-Analysis. *J Acad Nutr Diet.* 2019;119(5):799-817 e743. PMID: 30833172	Population
356. Tian Y, Su L, Wang J, Duan X, Jiang X. Fruit and vegetable consumption and risk of the metabolic syndrome: a meta-analysis. *Public Health Nutr.* 2018;21(4):756-765. PMID: 29151369	Outcome
357. Toews I, Lohner S, Kullenberg de Gaudry D, Sommer H, Meerpohl JJ. Association between intake of non-sugar sweeteners and health outcomes: systematic review and meta-analyses of randomised and non-randomised controlled trials and observational studies. *BMJ.* 2019;364:k4718. PMID: 30602577	No nutrition intervention of interest
358. Touyz LM, Wakefield CE, Grech AM, et al. Parent-targeted home-based interventions for increasing fruit and vegetable intake in children: a systematic review and meta-analysis. *Nutr Rev.* 2018;76(3):154-173. PMID: 29319789	Outcome
359. Trasande L, Shaffer RM, Sathyanarayana S, Council On Environmental H. Food Additives and Child Health. *Pediatrics.* 2018;142(2). PMID: 30037972	Study Design
360. Trieu K, McMahon E, Santos JA, et al. Review of behaviour change interventions to reduce population salt intake. *Int J Behav Nutr Phys Act.* 2017;14(1):17. PMID: 28178990	Population
361. Turbutt C, Richardson J, Pettinger C. The impact of hot food takeaways near schools in the UK on childhood obesity: a systematic review of the evidence. *J Public Health (Oxf).* 2019;41(2):231-239. PMID: 29590382	No meta-analysis or graded certainty of evidence
362. Utz-Melere M, Targa-Ferreira C, Lessa-Horta B, Epifanio M, Mouzaki M, Mattos AA. Non-Alcoholic Fatty Liver Disease in Children and Adolescents: Lifestyle Change - a Systematic Review and Meta-Analysis. *Ann Hepatol.* 2018;17(3):345-354. PMID: 29735796	SR does not have 2 databases or ROB assessment

Articles	Reason for Exclusion
363. Valerio G, Maffeis C, Saggese G, et al. Diagnosis, treatment and prevention of pediatric obesity: consensus position statement of the Italian Society for Pediatric Endocrinology and Diabetology and the Italian Society of Pediatrics. *Ital J Pediatr.* 2018;44(1):88. pMID: 30064525	Study Design
364. Vallabhan MK, Jimenez EY, Nash JL, et al. Motivational Interviewing to Treat Adolescents With Obesity: A Meta-analysis. *Pediatrics.* 2018;142(5). PMID: 30348753	Population
365. van Baak MA, Mariman ECM. Dietary Strategies for Weight Loss Maintenance. *Nutrients.* 2019;11(8). PMID: 31443231	SR does not have 2 databases or ROB assessment
366. van Hoek E, Bouwman LI, Koelen MA, Lutt MAJ, Feskens EJM, Janse AJ. Development of a Dutch intervention for obese young children. *Health Promot Int.* 2017;32(4):624-635. PMID: 26755807	SR does not have 2 databases or ROB assessment
367. Vander Wyst KB, Whisner CM, Reifsnider E, Petrov ME. The Combined Impact of Sleep and Diet on Adiposity in Infants, Toddlers, and Young Children: A Systematic Review. *J Dev Behav Pediatr.* 2019;40(3):224-236. PMID: 30741778	No nutrition intervention of interest
368. Vanderhout SM, Aglipay M, Torabi N, et al. Whole milk compared with reduced-fat milk and childhood overweight: a systematic review and meta-analysis. *Am J Clin Nutr.* 2020;111(2):266-279. PMID: 31851302	No nutrition intervention of interest
369. Velazquez CE, Black JL, Potvin Kent M. Food and Beverage Marketing in Schools: A Review of the Evidence. *Int J Environ Res Public Health.* 2017;14(9). PMID: 28895921	SR does not have 2 databases or ROB assessment
370. Vercammen KA, Frelier JM, Lowery CM, McGlone ME, Ebbeling CB, Bleich SN. A systematic review of strategies to reduce sugar-sweetened beverage consumption among 0-year to 5-year olds. *Obes Rev.* 2018;19(11):1504-1524. PMID: 3009442	Outcome
371. Verduci E, Martelli A, Miniello VL, et al. Nutrition in the first 1000 days and respiratory health: A descriptive review of the last five years' literature. *Allergol Immunopathol (Madr).* 2017;45(4):405-413. PMID: 28411961	Population
372. Verhage CL, Gillebaart M, van der Veek SMC, Vereijken C. The relation between family meals and health of infants and toddlers: A review. *Appetite.* 2018;127:97-109. PMID: 29654851	Population

Articles	Reason for Exclusion
373. Verjans-Janssen SRB, van de Kolk I, Van Kann DHH, Kremers SPJ, Gerards S. Effectiveness of school-based physical activity and nutrition interventions with direct parental involvement on children's BMI and energy balance-related behaviors - A systematic review. *PLoS One.* 2018;13(9):e0204560. PMID: 30261057	No meta-analysis or graded certainty of evidence
374. Vezina-Im LA, Beaulieu D, Belanger-Gravel A, et al. Efficacy of school-based interventions aimed at decreasing sugar-sweetened beverage consumption among adolescents: a systematic review. *Public Health Nutr.* 2017;20(13):2416-2431. PMID: 28173882	Outcome
375. Vilallonga R, Moreno Villares JM, Yeste Fernandez D, et al. Initial Approach to Childhood Obesity in Spain. A Multisociety Expert Panel Assessment. *Obes Surg.* 2017;27(4):997-1006. PMID: 27752806	Study Design
376. Villasana MV, Pires IM, Sa J, et al. Promotion of Healthy Nutrition and Physical Activity Lifestyles for Teenagers: A Systematic Literature Review of The Current Methodologies. *J Pers Med.* 2020;10(1). PMID: 30298750	SR does not have 2 databases or ROB assessment
377. Villegas-Navas V, Montero-Simo MJ, Araque-Padilla RA. The Effects of Foods Embedded in Entertainment Media on Children's Food Choices and Food Intake: A Systematic Review and Meta-Analyses. *Nutrients.* 2020;12(4). PMID: 32244299	Outcome
378. Visser J, McLachlan MH, Maayan N, Garner P. Community-based supplementary feeding for food insecure, vulnerable and malnourished populations - an overview of systematic reviews. *Cochrane Database Syst Rev.* 2018;11:CD010578. PMID: 30480324	Other
379. Viswanathan M, Treiman KA, Doto JK, Middleton JC, Coker-Schwimmer EJ, Nicholson WK. *Folic Acid Supplementation: An Evidence Review for the U.S. Preventive Services Task Force.* Rockville (MD): Agency for Healthcare Research and Quality (US; Jan 2017. Report No.: 14-05214-EF-1. PMID: 28151610	Population
380. Volpe M, Battistoni A, Gallo G, et al. Executive Summary of the 2018 Joint Consensus Document on Cardiovascular Disease Prevention in Italy. *High Blood Press Cardiovasc Prev.* 2018;25(3):327-341. PMID: 30232768	Study Design
381. von Philipsborn P, Stratil JM, Burns J, et al. Environmental interventions to reduce the consumption of sugar-sweetened beverages and their effects on health. *Cochrane Database Syst Rev.* 2019;6:CD012292. PMID: 31194900	Study Design
382. Vos MB, Abrams SH, Barlow SE, et al. NASPGHAN Clinical Practice Guideline for the Diagnosis and Treatment of Nonalcoholic Fatty Liver Disease in Children: Recommendations from the Expert Committee on NAFLD (ECON) and the North American Society of Pediatric Gastroenterology, Hepatology and Nutrition (NASPGHAN). *J Pediatr Gastroenterol Nutr.* 2017;64(2):319-334. PMID: 28107283	Study Design

Articles	Reason for Exclusion
383. Wahi G, de Souza RJ, Hartmann K, Giglia L, Jack SM, Anand SS. Effectiveness of programs aimed at obesity prevention among Indigenous children: A systematic review. Prev Med Rep. 2021;22:101347.	No nutrition intervention of interest
384. Walker M, McPherson AC. Weight management services for an underserved population: a rapid review of the literature. *Disabil Rehabil.* 2020;42(2):274-282. PMID: 30298750	SR does not have 2 databases or ROB assessment
385. Wang AY, Dhaliwal J, Mouzaki M. Lean non-alcoholic fatty liver disease. *Clin Nutr.* 2019;38(3):975-981. PMID: 30466956	SR does not have 2 databases or ROB assessment
386. Wang Halpern L. Updated Pediatric Blood Pressure Guidelines: Simplified guidance may ease management of high blood pressure in children. *AM J NURS.* 2017;117(12):17. DOI: 10.1097/01.NAJ.0000527474.33127.0e	Study Design
387. Ward AL, Reynolds AN, Kuroko S, Fangupo LJ, Galland BC, Taylor RW. Bidirectional associations between sleep and dietary intake in 0-5 year old children: A systematic review with evidence mapping. *Sleep Med Rev.* 2020;49:101231. PMID: 31783229	Population
388. Ward DS, Welker E, Choate A, et al. Strength of obesity prevention interventions in early care and education settings: A systematic review. *Prev Med.* 2017;95 Suppl:S37-S52. PMID: 27693295	Outcome
389. Wei B, Liu Y, Lin X, Fang Y, Cui J, Wan J. Dietary fiber intake and risk of metabolic syndrome: A meta-analysis of observational studies. *Clin Nutr.* 2018;37(6 Pt A):1935-1942. PMID: 29137803	SR does not have 2 databases or ROB assessment
390. Welbourn R, Hopkins J, Dixon JB, et al. Commissioning guidance for weight assessment and management in adults and children with severe complex obesity. *Obes Rev.* 2018;19(1):14-27.PMID: 209024367	Population
391. Wethington HR, Finnie RKC, Buchanan LR, et al. Healthier Food and Beverage Interventions in Schools: Four Community Guide Systematic Reviews. American journal of preventive medicine. 2020;59(1):e15-e26.	No meta-analysis or graded certainty of evidence
392. Wickham CA, Carbone ET. What's technology cooking up? A systematic review of the use of technology in adolescent food literacy programs. *Appetite.* 2018;125:333-344. PMID: 29471069	Outcome

Articles	Reason for Exclusion
393. Winkler MR, Bennett GG, Brandon DH. Factors related to obesity and overweight among Black adolescent girls in the United States. *Women Health.* 2017;57(2):208-248. PMID: 26933972	SR does not have 2 databases or ROB assessment
394. Wolf VLW, Samur-San-Martin JE, Sousa SF, et al. Effectiveness of Obesity Intervention Programs Based on Guidelines for Adolescent Students: Systematic Review. *Rev Paul Pediatr.* 2019;37(1):110-120. PMID: 30110114	SR does not have 2 databases or ROB assessment
395. Wong MM, Arcand J, Leung AA, Thout SR, Campbell NR, Webster J. The science of salt: A regularly updated systematic review of salt and health outcomes (December 2015-March 2016). *J Clin Hypertens (Greenwich).* 2017;19(3):322-332. PMID: 28266792	SR does not have 2 databases or ROB assessment
396. World Health Organization. *Guideline: Assessing and Managing Children at Primary Health-Care Facilities to Prevent Overweight and Obesity in the Context of the Double Burden of Malnutrition: Updates for the Integrated Management of Childhood Illness (IMCI).* Geneva: World Health Organization;2017. PMID: 29578661	Population
397. Wolfenden L, Nathan NK, Sutherland R, et al. Strategies for enhancing the implementation of school-based policies or practices targeting risk factors for chronic disease. Cochrane Database Syst Rev. 2017;11(11):Cd011677.	No nutrition intervention of interest
398. Wolfenden L, Barnes C, Jones J, et al. Strategies to improve the implementation of healthy eating, physical activity and obesity prevention policies, practices or programmes within childcare services. Cochrane Database Syst Rev. 2020;2(2):Cd011779.	No nutrition intervention of interest
399. World Health Organization. *Guideline: implementing effective actions for improving adolescent nutrition.* Geneva: World Health Organization; 2018.	Study Design
400. Wu XY, Zhuang LH, Li W, et al. The influence of diet quality and dietary behavior on health-related quality of life in the general population of children and adolescents: a systematic review and meta-analysis. *Qual Life Res.* 2019;28(8):1989-2015. PMID: 30875010	Outcome
401. Wylie-Rosett J, Hu FB. Nutritional Strategies for Prevention and Management of Diabetes: Consensus and Uncertainties. *Diabetes Care.* 2019;42(5):727-730. PMID: 31010943	Study Design
402. Xin J, Zhao L, Wu T, et al. Association between access to convenience stores and childhood obesity: A systematic review. *Obes Rev.* 2021;22 Suppl 1:e12908. PMID: 31274248	Study Design

Articles	Reason for Exclusion
403. Yang S, Zhang X, Feng P, et al. Access to fruit and vegetable markets and childhood obesity: A systematic review. *Obes Rev.* 2021;22 Suppl 1:e12980. PMID: 31943666	Study Design
404. Yau KW, Tang TS, Görges M, et al. Effectiveness of Mobile Apps in Promoting Healthy Behavior Changes and Preventing Obesity in Children: Systematic Review. JMIR Pediatr Parent. 2022;5(1):e34967.	No nutrition intervention of interest
405. Yee AZ, Lwin MO, Ho SS. The influence of parental practices on child promotive and preventive food consumption behaviors: a systematic review and meta-analysis. *Int J Behav Nutr Phys Act.* 2017;14(1):47. PMID: 28399881	SR does not have 2 databases or ROB assessment
406. Yi DY, Kim SC, Lee JH, et al. Clinical Practice Guideline for the Diagnosis and Treatment of Pediatric Obesity: Recommendations from the Committee on Pediatric Obesity of the Korean Society of Pediatric Gastroenterology Hepatology and Nutrition. *Pediatr Gastroenterol Hepatol Nutr.* 2019;22(1):1-27. PMID: 30671370	Other
407. Young KG, Duncanson K, Burrows T. Influence of grandparents on the dietary intake of their 2-12-year-old grandchildren: A systematic review. *Nutr Diet.* 2018;75(3):291-306. PMID: 29446218	No meta-analysis or graded certainty of evidence
408. Zafar MI, Mills KE, Zheng J, et al. Low-glycemic index diets as an intervention for diabetes: a systematic review and meta-analysis. *Am J Clin Nutr.* 2019;110(4):891-902. PMID: 31374573	Population
409. Zalewski BM, Patro B, Veldhorst M, et al. Nutrition of infants and young children (one to three years) and its effect on later health: A systematic review of current recommendations (EarlyNutrition project). *Crit Rev Food Sci Nutr.* 2017;57(3):489-500. PMID: 25751102	Population
410. Zarnowiecki D, Mauch CE, Middleton G, et al. A systematic evaluation of digital nutrition promotion websites and apps for supporting parents to influence children's nutrition. *Int J Behav Nutr Phys Act.* 2020;17(1):17. PMID: 32041640	Outcome
411. Zeitler P, Arslanian S, Fu J, et al. ISPAD Clinical Practice Consensus Guidelines 2018: Type 2 diabetes mellitus in youth. *Pediatr Diabetes.* 2018;19 Suppl 27:28-46. PMID: 29999228	Study Design
412. Zhang Q, Liu R, Diggs LA, Wang Y, Ling L. Does acculturation affect the dietary intakes and body weight status of children of immigrants in the U.S. and other developed countries? A systematic review. *Ethn Health.* 2019;24(1):73-93. PMID: 28406037	SR does not have 2 databases or ROB assessment

Articles	Reason for Exclusion
413. Zhang S, de la Haye K, Ji M, An R. Applications of social network analysis to obesity: a systematic review. *Obes Rev.* 2018;19(7):976-988. PMID: 29676508	No nutrition intervention of interest
414. Zhao L, Dong X, Gao Y, et al. Effects of exercise combined with diet intervention on body composition and serum biochemical markers in adolescents with obesity: a systematic review and meta-analysis. J Pediatr Endocrinol Metab. 2022;35(11):1319-1336.	Population

Supplementary Table S7. AMSTAR2 Ratings in Critical Systematic Review Domains and Overall Certainty in Results for Systematic Reviews included in the Pediatric Overweight and Obesity Prevention Position Paper

Systematic Review	2. Explicit statement of a priori review methods	4. Comprehensive literature search strategy	7. List of excluded studies with justification	9. Satisfactory technique for assessing risk of bias	11. If meta-analysis, appropriate methods of combining results	13. Account for risk of bias of included studies when interpreting result	15. If quantitative analysis, investigation and discussion of impact of publication bias	Overall Confidence in Results of Review
Abdel Rahman et al 2018	N	Y	N	Y	Y	Y	Y	Critically Low
Ananthapavan et al 2018	N	Y	N	Y	Y	N	N	Critically Low
Bleich et al 2018	N	Y	N	Y	No Meta	Y	No Meta	Critically Low
Brown et al 2019	Y	Y	Y	Y	Y	Y	Y	High
Buchanan et al 2023	Y	Y	N	Y	N	N	N	Critically Low
Caulfield et al 2022	Y	Y	Y	Y	No Meta	Y	No Meta	Moderate
Cerrato-Carretero et al 2021	N	Y	N	Y	Y	N	Y	Critically Low
Cohen et al 2021	Y	Y	N	Y	No Meta	Y	No Meta	Low
Dabravolskaj et al 2020	N	Y	N	Y	Y	N	Y	Critically Low
Durão et al 2023	Y	Y	Y	Y	Y	Y	Y	Moderate
Gonclaves et al 2021	Y	Y	Y	Y	Y	Y	Y	Moderate

Systematic Review	2. Explicit statement of a priori review methods	4. Comprehensive literature search strategy	7. List of excluded studies with justification	9. Satisfactory technique for assessing risk of bias	11. If meta-analysis, appropriate methods of combining results	13. Account for risk of bias of included studies when interpreting result	15. If quantitative analysis, investigation and discussion of impact of publication bias	Overall Confidence in Results of Review
Hayba et al 2020	Y	Y	Y	Y	No Meta	Y	No Meta	High
Hudak et al 2019	N	Y	N	N	No Meta	Y	No Meta	Critically Low
Kenny et al 2022	Y	Y	N	Y	No Meta	N	No Meta	Critically Low
Kornet-van der Aa et al 2017	Y	Y	N	Y	No Meta	Y	No Meta	Moderate
Long et al 2021	N	Y	N	Y	Y	Y	Y	Critically Low
McHugh et al 2020	Y	Y	N	Y	No Meta	N	No Meta	Moderate
Morgan et al 2020	Y	Y	Y	Y	No Meta	Y	No Meta	High
Nally et al 2021	Y	Y	N	Y	Y	N	Y	Critically Low
Narzisi et al 2020	N	Y	N	Y	No Meta	N	No Meta	Critically Low
Nury et al 2021	Y	Y	Y	Y	Y	Y	Y	High
Olstad et al 2017	Y	Y	N	Y	No Meta	Y	No Meta	Moderate
Pineda et al 2021	Y	N	N	Y	Y	N	Y	Critically Low

Systematic Review	2. Explicit statement of a priori review methods	4. Comprehensive literature search strategy	7. List of excluded studies with justification	9. Satisfactory technique for assessing risk of bias	11. If meta-analysis, appropriate methods of combining results	13. Account for risk of bias of included studies when interpreting result	15. If quantitative analysis, investigation and discussion of impact of publication bias	Overall Confidence in Results of Review
Qi et al 2021	Y	N	N	Y	Y	N	Y	Critically Low
Rochira et al 2020	N	Y	N	Y	Y	N	N	Critically Low
Salam et al 2020	Y	Y	N	Y	Y	Y	N	Low
Serel-Cortes et al 2021	Y	Y	N	Y	Y	Y	N	Critically Low
Silva et al 2022	Y	Y	N	N	Y	Y	Y	Critically Low
Singh et al 2017	N	Y	N	Y	No Meta	Y	No Meta	Critically Low
Smit et al 2023	Y	Y	Y	Y	Y	Y	Y	High
Specchia et al 2018	N	Y	N	Y	Y	Y	Y	Low
Suleiman-Martos et al 2021	N	Y	N	Y	Y	N	Y	Critically Low
Tissot et al 2021	Y	Y	N	Y	No Meta	Y	No Meta	Moderate
Wethington et al 2020	N	Y	N	N	No Meta	N	No Meta	Critically Low
Whitehead et al 2021	Y	Y	Y	Y	No Meta	N	No Meta	Low

Supplementary Table S8. Summary of Findings Table Describing Systematic Reviews Examining the Effects of Nutrition Interventions for Pediatric Obesity Prevention in Home & Family Settings in individuals 2-17 years of age.

Outcome<br>№ of participants<br>(studies)	**Anticipated absolute effects (95% CI)**	Certainty	What happens
	**Difference**		
**Combined Age Groups**			
BMI[1]<br>№ of participants: 1861<br>(4 RCTs)	SMD **0.05** higher<br>(0.04 lower to 0.15 higher)	⊕⊕◯◯<br>LOW [a,b]	Interventions involving a caregiver in nutrition and physical activity interventions may result in little to no difference in BMI compared to interventions with a caregiver for children and adolescents 2-18 years of age.
Prevalence of overweight/obesity[1]<br>№ of participants: 1866<br>(3 RCTs)	RR: 1.02 (0.89, 1.17)	⊕⊕◯◯<br>LOW [a,b]	Interventions involving a caregiver in nutrition and physical activity interventions may result in little to no difference in overweight or obesity prevalence compared to interventions with a caregiver for children and adolescents 2-18 years of age.
**0-5 years**			
BMI z-score[2]<br>№ of participants: 595<br>(3 RCTs)	MD **0.13 lower**<br>(0.35 lower to 0.09 higher)	⊕⊕◯◯<br>LOW [a,b]	Nutrition and physical activity interventions in the home setting may have no affect on BMI z-score in children 0-5 years of age.
BMI[2]<br>№ of participants: 778<br>(2 RCTs)	MD **0.33 kg/m² lower**<br>(0.55 lower to 0.1 lower)	⊕⊕⊕⊕<br>HIGH	Nutrition and physical activity interventions in the home setting reduce BMI in children 0-5 years of age.
**6-12 years**			
BMI z-score[2]<br>№ of participants: 134<br>(1 RCT)	MD **0 0.03**<br>(0.04 lower to 0.1 higher)	⊕⊕◯◯<br>LOW [ab]	One study described a nutrition and physical activity intervention in the home setting may result in little to no difference in BMI z-score in children 6-12 years of age.
**13-17 years**			

Outcome<br>№ of participants<br>(studies)	Anticipated absolute effects (95% CI)	Certainty	What happens
	**Difference**		
BMI z-score[2]<br>№ of participants: 75<br>(1 RCT)	MD **0.06 higher**<br>(0.13 lower to 0.26 higher)	⊕◯◯◯<br>VERY LOW [ac]	The evidence is very uncertain about the effect of nutrition and physical activity interventions in the home setting on BMI z-score for adolescents 13-18 years of age.

Explanations

a. Risk of bias in included studies.
b. Small sample size or wide confidence interval, especially crossing the line of no effect.
c. Marked down two levels for imprecision due to very low sample size and wide confidence interval crossing the line of no effect.

Supplementary Table S9. Summary of Findings Table Describing Systematic Reviews Examining the Effects of Nutrition Interventions for Pediatric Obesity Prevention in Healthcare Settings in individuals 2-17 years of age.

Outcome<br>№ of participants<br>(studies)	Anticipated absolute effects (95% CI)<br>Difference	Certainty	What happens
**Age Groups Combined**			
BMI z-score and percentile[3]<br>№ of participants: 1750<br>(9 RCTs)	not pooled	⨁◯◯◯<br>VERY LOW<br>abc	The effect of nutrition and physical activity interventions in the healthcare setting on BMI z-scores and percentiles is uncertain.
**0-5 years**			
BMI z-score[2]<br>№ of participants: 121<br>(1 RCT)	MD **0.24 lower**<br>(0.46 lower to 0.02 lower)	⨁⨁◯◯<br>LOW a,c	Nutrition and physical activity interventions in the healthcare setting may reduce BMI z-score in children 0-5 years of age.
Prevalence of overweight[4]<br>№ of participants: Unclear<br>(1 RCT)	NR	⨁◯◯◯<br>VERY LOW<br>acd	The effect of nutrition and physical activity interventions on prevalence of overweight in children ≤5 years of age in the healthcare setting is uncertain.

Explanations

a. Risk of bias in included studies.
b. Inconsistent results between studies.
c. Small sample size
d. No effect size described.

Supplementary Table S10. Summary of Findings Table Describing Systematic Reviews Examining the Effects of Nutrition Interventions for Pediatric Obesity Prevention in School Settings in individuals 2-17 years of age.

Outcome<br>№ of participants<br>(studies)	**Anticipated absolute effects (95% CI)**<br>**Difference**	Certainty	What happens
**Age Groups Combined**			
BMI z-score[5]<br>№ of participants: 8174<br>(8 RCTs)	MD (95% CI): -0.09 (-0.18, 0.00)	⊕⊕◯◯<br>LOW[ab]	In all age groups of children and adolescents combined, nutritional interventions to prevent pediatric obesity in the school setting may not affect BMI z-scores.
BMI[5]<br>№ of participants: 12067<br>(10 RCTs)	MD (95% CI): [0.03 (-0.10, 0.16)]	⊕⊕◯◯<br>LOW[ab]	In all age groups of children and adolescents combined, nutritional interventions to prevent pediatric obesity in the school setting may not affect BMI.
Overweight and Obesity Prevalence[5]<br><br>№ of participants: 901<br>(3 RCTs)	OR (95% CI): 1.19 (0.95, 1.49)	⊕◯◯◯<br>VERY LOW[aab]	In all age groups of children and adolescents combined, nutritional interventions to prevent pediatric obesity in the school setting may not affect prevalence of overweight and obesity.
**0-5 years**			
BMI z-score[2]<br>№ of participants: 4913<br>(10 RCTs)	MD **0.04 lower**<br>(0.09 lower to 0.01 higher)	⊕⊕◯◯<br>LOW [ab]	In children ≤ 5 years of age, nutrition and physical activity interventions in the childcare and preschool settings may not affect BMI z-score.
BMI[2]<br>№ of participants: 4683<br>(9 RCTs)	MD **0.05 kg/m2 lower**<br>(0.14 lower to 0.05 higher)	⊕⊕◯◯<br>LOW [a,b]	In children ≤ 5 years of age, nutrition and physical activity interventions in the childcare and preschool settings may not affect BMI.
**6-12 years**			

Outcome<br>№ of participants<br>(studies)	Anticipated absolute effects (95% CI)	Certainty	What happens
	**Difference**		
BMI z-score[2]<br>№ of participants: 22879<br>(15 RCTs)	MD **0.05 lower**<br>(0.1 lower to 0.01 lower)	⊕⊕⊕◯<br>MODERATE [a]	Nutrition and physical activity interventions in the school setting likely reduces BMI z-score in children 6-12 years of age.
BMI[2]<br>№ of participants: 18488<br>(16 RCTs)	MD **0.04 lower**<br>(0.1 lower to 0.02 higher)	⊕⊕◯◯<br>LOW [a,b]	Nutrition and physical activity interventions in the school setting may not affect BMI in children 6-12 years of age.
Overweight Prevalence[6]<br>№ of participants: 7059<br>(9 RCTs)	Not pooled	⊕⊕◯◯<br>LOW [a,b]	Nutrition and physical activity interventions in the school setting may not affect overweight prevalence in children 6-12 years of age.
**13-17 years**			
BMI z-score<br>№ of participants: 16173<br>(5 RCTs)	**0**<br>(0.06 lower to 0.06 higher)	⊕◯◯◯<br>VERY LOW [a,b,c]	Nutrition and physical activity interventions in the school setting may not affect BMI z-score in adolescents 13-18 years of age, but evidence is uncertain.
BMI<br>№ of participants: 16347<br>(8 RCTs)	MD **0.02 lower**<br>(0.1 lower to 0.05 higher)	⊕⊕◯◯<br>LOW [a,c]	Nutrition and physical activity interventions in the school setting may not affect BMI in adolescents 13-18 years of age.

Explanations

a. Risk of bias in included primary studies.
b. Small sample size or wide confidence interval, especially one that crosses the line of no effect.
c. Inconsistency in results between studies

Supplementary Table S11. Summary of Findings Table Describing Systematic Reviews Examining the Effects of Nutrition Interventions for Pediatric Obesity Prevention in Community Settings in individuals 2-17 years of age.

Outcome<br>№ of participants<br>(studies)	Anticipated absolute effects (95% CI)	Certainty	What happens
	**Difference**		
**All Age Groups**			
BMI z-score[7]<br>№ of participants: Unclear<br>(9 Trials)	MD -0.07 (-0.13, -0.01)	⊕⊕◯◯<br>LOW[ab]	Nutrition and physical activity interventions in the community setting may reduce BMI z-score in children 5-18 years of age.
Cost-effectiveness[7]<br>№ of participants: Unclear<br>(6 RCTs)	ICER $8155AUD ($237 to $81,021) per HALY	⊕⊕◯◯<br>LOW[ac]	Nutrition and physical activity interventions to prevent obesity in Australian community-based interventions may be cost-effective in participants 2-18 years of age.
**0-5 years**			
BMI z-score[2]<br>№ of participants: 632<br>(2 RCTs)	MD **0.02 lower**<br>(0.13 lower to 0.09 higher)	⊕⊕◯◯<br>LOW[ac]	Nutrition and physical activity interventions in the community setting may have no effect on BMI z-score children 0-5 years of age.
BMI<br>№ of participants: 75<br>(1 RCT)	MD **0.59 kg/m² lower**<br>(0.94 lower to 0.24 lower)	⊕◯◯◯<br>Very LOW<br>[a,c,c]	Nutrition and physical activity interventions in the community setting may reduce BMI in children 0-5 years, but evidence is uncertain.
**6-12 years**			
BMI z-score<br>№ of participants: 657<br>(4 RCTs)	MD **0.04 lower**<br>(0.39 lower to 0.31 higher)	⊕◯◯◯<br>VERY LOW<br>[a,c]	The evidence is very uncertain and heterogeneous in regards to the effect of nutrition and physical interventions in the community setting on BMI z-score in children 6-12 years of age.

Outcome<br>№ of participants<br>(studies)	Anticipated absolute effects (95% CI)	Certainty	What happens
	**Difference**		
BMI<br>№ of participants: 751<br>(9 RCTs)	MD **0.08 kg/m2 lower**<br>(0.29 lower to 0.13 higher)	⊕⊕◯◯<br>LOW [d]	Nutrition and physical activity interventions in the community may result in little to no difference in BMI in children 6-12 years of age.
**13-17 years**			
BMI z-score<br>№ of participants: Unclear<br>(4 RCTs)	MD **0.02 lower**<br>(0.07 lower to 0.03 higher)	⊕◯◯◯<br>VERY LOW [a,c,d]	The evidence is very uncertain and heterogeneous in regards to the effect of nutrition and physical interventions in the community setting on BMI z-score in children 6-12 years of age.

Explanations

a. Risk of bias in included primary studies.
b. Inconsistency in results between studies
c. Small sample size or wide confidence interval, especially one that crosses the line of no effect.
d. Sample size unclear

Supplementary Table S12. Summary of Findings Table Describing Systematic Reviews Examining the Effects of Federal Food Assistance Programs for Pediatric Obesity Prevention in Individuals 2-17 years of age.

Outcome<br>№ of participants<br>(studies)	Difference	Certainty	What happens
SNAP Program[8]<br>№ of participants: Unclear<br>9 observational studies	not pooled	⊕◯◯◯<br>VERY LOW [a,b,c]	The evidence is very uncertain about the effect of the SNAP program on weight outcomes in pediatric participants 2-18 years of age, with some evidence suggesting increased risk of overweight or obesity for certain sub-populations, including girls 5-18 years of age.
USDA Fresh Fruits and Vegetables Program[9]<br>№ of participants: 335<br>(1 observational study)	not pooled	⊕◯◯◯<br>VERY LOW[b,d]	The evidence is very uncertain about the effect of fresh fruit and vegetable programs on anthropometrics in children elementary school age but outside of the school setting. However, limited results suggest that 2 years of policy exposure resulted in decreased BMI z-scores and BMI.
CACFP Program[10]<br>№ of participants: 4050<br>(1 observational study)	Single study	⊕◯◯◯<br>VERY LOW[e]	The evidence is very uncertain about the impact of the impact of the CACFP program on pediatric obesity prevention, but one study described no association with prevalence of overweight or obesity.
Universal School Meals[11]<br>№ of participants: Unclear<br>(5 controlled trials, 2 non-controlled trials)	not pooled	⊕◯◯◯<br>VERY LOW[ce]	The evidence is very uncertain about the impact of the impact of universal school meals on pediatric obesity prevention, but limited evidence described there may be no association.
2009 WIC Food Package Revision[12]<br>№ of participants: >16 million<br>(3 observational studies)	not pooled	⊕◯◯◯<br>LOW[e]	The evidence is very uncertain about the impact of the 2009 revised WIC package, but the revised package may have decreased prevalence of overweight and obesity in children ≤5 years of age.

Explanations

a. Inconsistency in results between studies.

b. No quantitative results available

c. Sample sizes not provided.

d. Small sample size

e. Risk of bias in included studies

Supplementary Table S13. Summary of Findings Table Describing Systematic Reviews Examining the Effects of Nutrition Interventions for Pediatric Obesity Prevention in Electronic Interventions in individuals 2-17 years of age.

Outcome<br>№ of participants<br>(studies)	Anticipated absolute effects (95% CI)	Certainty	What happens
	Difference		
BMI z-score[13]<br>№ of participants:<br>571<br>(2 RCTs)	MD **0.05 SD higher**<br>(0.21 lower to 0.11 higher)	⨁⨁◯◯<br>LOW [ab]	Gamification including nutrition may have no effect on BMI z-score in children and adolescents.
BMI[14]<br>№ of participants:<br>3542<br>(9 RCTs)	MD **0.02 lower**<br>(0.18 lower to 0.14 higher)	⨁⨁⨁◯<br>MODERATE[c]	Mobile phone interventions did not affect BMI for adolescents 10-19 years of age.

Explanations

a. Small sample size
b. Wide confidence interval, especially if crossing the line of no effect.
c. Risk of bias in included studies.

References

1. Morgan EH, Schoonees A, Sriram U, Faure M, Seguin-Fowler RA. Caregiver involvement in interventions for improving children's dietary intake and physical activity behaviors. *Cochrane Database Syst Rev.* 2020;1(1):Cd012547.
2. Brown T, Moore TH, Hooper L, et al. Interventions for preventing obesity in children. *Cochrane Database Syst Rev.* 2019;7(7):Cd001871.
3. Tissot H, Pfarrwaller E, Haller DM. Primary care prevention of cardiovascular risk behaviors in adolescents: A systematic review. *Preventive medicine.* 2021;142:106346.
4. Narzisi K, Simons J. Interventions that prevent or reduce obesity in children from birth to five years of age: A systematic review. *Journal of child health care : for professionals working with children in the hospital and community.* 2020:1367493520917863.
5. Nury E, Stadelmaier J, Morze J, et al. Effects of nutritional intervention strategies in the primary prevention of overweight and obesity in school settings: systematic review and network meta-analysis. *BMJ Med.* 2022;1(1):e000346.
6. Smit MS, Boelens M, Mölenberg FJM, Raat H, Jansen W. The long-term effects of primary school-based obesity prevention interventions in children: A systematic review and meta-analysis. *Pediatr Obes.* 2023;18(3):e12997.
7. Ananthapavan J, Nguyen PK, Bowe SJ, et al. Cost-effectiveness of community-based childhood obesity prevention interventions in Australia. *Int J Obes (Lond).* 2019;43(5):1102-1112.
8. Hudak KM, Racine EF. The Supplemental Nutrition Assistance Program and Child Weight Status: A Review. *Am J Prev Med.* 2019;56(6):882-893.
9. Olstad DL, Ancilotto R, Teychenne M, et al. Can targeted policies reduce obesity and improve obesity-related behaviours in socioeconomically disadvantaged populations? A systematic review. *Obes Rev.* 2017;18(7):791-807.
10. Kenney EL, Tucker K, Plummer RS, Mita C, Andreyeva T. The Child and Adult Care Food Program and young children's health: a systematic review. *Nutr Rev.* 2023.
11. Cohen JFW, Hecht AA, McLoughlin GM, Turner L, Schwartz MB. Universal School Meals and Associations with Student Participation, Attendance, Academic Performance, Diet Quality, Food Security, and Body Mass Index: A Systematic Review. *Nutrients.* 2021;13(3).
12. Caulfield LE, Bennett WL, Gross SM, et al. AHRQ Comparative Effectiveness Reviews. In: *Maternal and Child Outcomes Associated With the Special Supplemental Nutrition Program for Women, Infants, and Children (WIC).* Rockville (MD): Agency for Healthcare Research and Quality (US); 2022.
13. Suleiman-Martos N, García-Lara RA, Martos-Cabrera MB, et al. Gamification for the Improvement of Diet, Nutritional Habits, and Body Composition in Children and Adolescents: A Systematic Review and Meta-Analysis. *Nutrients.* 2021;13(7).
14. Beck Silva KB, Miranda Pereira E, Santana MLP, Costa PRF, Silva RCR. Effects of computer-based interventions on food consumption and anthropometric parameters of adolescents: A systematic review and metanalysis. *Crit Rev Food Sci Nutr.* 2022:1-13.
